# Identification of Novel *Drosophila melanogaster* MicroRNAs

**DOI:** 10.1371/journal.pone.0001265

**Published:** 2007-11-28

**Authors:** Thomas Sandmann, Stephen M. Cohen

**Affiliations:** 1 Temasek Lifesciences Laboratory, Department of Biological Sciences, National University of Singapore, Singapore, Singapore; 2 European Molecular Biology Laboratory, Heidelberg, Germany; Institute for Research in Biomedicine, Spain

## Abstract

MicroRNAs (miRNAs) are small non-coding RNAs with important regulatory roles in post-transcriptional regulation of metazoan development, homeostasis and disease. The full set of miRNAs is not known for any species and it is believed that many await discovery. The recent assembly of 15 insect genomes has provided the opportunity to identify novel miRNAs in the fruit fly, *Drosophila melanogaster.* We have performed a computational screen for novel microRNAs in *Drosophila melanogaster* by searching for phylogenetically conserved putative pre-miRNA structures. The ability of predicted novel miRNA precursors to be processed to produce miRNAs was experimentally verified in S2 cells and in several cases their endogenous expression at was validated by Northern blots. After experimental validation, the predictions were cross-checked with reference to a newly released set of small RNA sequences. Combining both datasets allowed us to identify 53 novel miRNA loci in the fruit fly genome 22 of which we had predicted computationally. This significantly expands the set of known miRNAs in *Drosophila melanogaster*. Most novel miRNAs contain unique seed sequences not found in other *Drosophila* miRNAs and are therefore expected to regulate novel sets of target genes. This data provides the basis for future genetic analysis of miRNA function and will aid the discovery of orthologous sequences in other species.

## Introduction

MicroRNAs (miRNAs) are small non-coding RNAs, typically 21–23 nt long, which serve as post-transcriptional regulators of gene expression. The founding members of this class, *lin-4* and *let-7* [1]–[3], were discovered using forward genetic screens in *C. elegans*. Subsequently large numbers of miRNAs have been identified by cloning and sequencing of short RNAs isolated from animals, plants and even the unicellular green *alga Chlamydomonas reinhardtii* [4]. To date, evidence for 533 human, 442 mouse and 93 *Drosophila* miRNAs has been deposited in mirBase (version 10.0) [5]. Based on comparison of miRNA predictions and sequence data it has been suggested that these genomes might contain many more, yet undiscovered, miRNA loci, with estimates of ∼1000 miRNAs in the human and ∼120 in the *Drosophila melanogaster* genome [6], [7].

MicroRNA genes have been found as independent transcription units as well as within introns of protein coding genes. More than half of the known loci are part of tandem arrays within operon-like clusters [8], containing up to 40 individual miRNAs [9]. Each primary miRNA transcript (pri-miRNA) contains an extended stem-loop structure, from which a precursor pre-miRNA is released through the action of the RNAseIII enzyme Drosha and the double-stranded RNA-binding domain (dsRBD) protein DGCR8/Pasha (reviewed in [10]). Additionally, a novel mechanism of pre-miRNA generation from very short introns through the splicing process itself has been reported recently [11], [12]. Animal pre-miRNAs are exported from the nucleus to the cytoplasm and processed further by the Dicer enzyme to yield a characteristic ∼22-nt miRNA duplex. The strand with the lower base-pairing energy at its 5′ end is then loaded onto the RNA-induced silencing complex (RISC) and guides it towards its regulatory targets [13], [14].

miRNAs mediate post-transcriptional inhibition by base-pairing with their cognate target mRNAs. Extended complementarity, as is typically found in plants, leads to cleavage of the target. Limited pairing, including but not limited to the 5′ seed region of the microRNA causes translational inhibition, and in some cases increased RNA turnover [15]–[18]. Evidence from microarray studies suggests that single miRNAs can target hundreds of messenger RNAs [19] and recent *in vivo* studies of loss-of-function phenotypes demonstrate the important regulatory role of miRNAs e.g. in development [20]–[23], homeostasis [24] or disease [25].

The majority of miRNAs known to date has been identified through cloning and sequencing of small RNA libraries. Additionally, complementary computational tools for predicting miRNAs have been developed. Two basic strategies have been described to predict novel microRNAs: Sequence similarity searches led to the identification of novel members of previously established miRNA families, many of which are conserved across the animal kingdom [26], [27]. Other approaches have successfully taken advantage of the well-defined secondary-structure constraints of pre-miRNA sequences as well as evolutionary conservation in related species to detect members of completely novel miRNA families [28], [29].

The publication of many newly sequenced and assembled genomes presents us with an unprecedented wealth of information about evolutionary conservation of coding and non-coding sequences. Based on the observation that the evolution of many non-coding RNAs (ncRNAs) is constrained by functionally important secondary structure elements, novel approaches have been developed to scan whole metazoan genomes for putative ncRNAs. The RNAz program employs a support vector machine (SVM) to evaluate both thermodynamic stability of candidate sequences as well as the conservation of the predicted secondary structure [30], [31]. Evofold uses stochastic context free grammars (SCFG) to identify conservation patterns indicating folded RNA structures [32]. Both programs are not limited to any particular class of ncRNA and have been used to predict several tens of thousands of putative ncRNAs in the human genome. To identify novel miRNAs in *Drosophila melanogaster*, we have combined a computational screen for generic ncRNAs using RNAz with stringent filtering criteria specifically targeting miRNA precursors. We have validated 22 of the newly predicted loci experimentally. 31 additional loci were identified from recently published small RNA sequence libraries.

## Results and Discussion

The fruit fly *Drosophila melanogaster* is a powerful model system to study metazoan development. Its compact genome is well annotated, owing to many years of genetic experimentation [33], and has been mined extensively for coding as well as non-coding gene loci [28], [34], [35]. With the assemblies of a range of additional insect genomes, information about twelve *Drosophila* species, the honeybee (*A. mellifera*), the flour beetle (*T. castaneum*) and the mosquito (*A. gambiae*) has recently become available. To identify novel microRNAs, we took advantage of the whole-genome alignments of 9 and 15 insect species provided by the UCSC genome browser [36]. Multiple sequence alignments for all “most conserved” regions, as identified by phastcons analysis [37], were extracted and scanned with RNAz for putative conserved non-coding sequences and several thousand candidate regions were recovered at a p-value cutoff of >0.9 (4003 with multiz9 and 17727 with multiz15 input alignments). At this threshold, 51 of the 65 previously known Drosophila miRNAs covered by the multiz9 and 51 of the 76 covered by the multiz15 input alignments were recovered (37/51 miRNAs were detected in both inputs).

**Figure 1:**
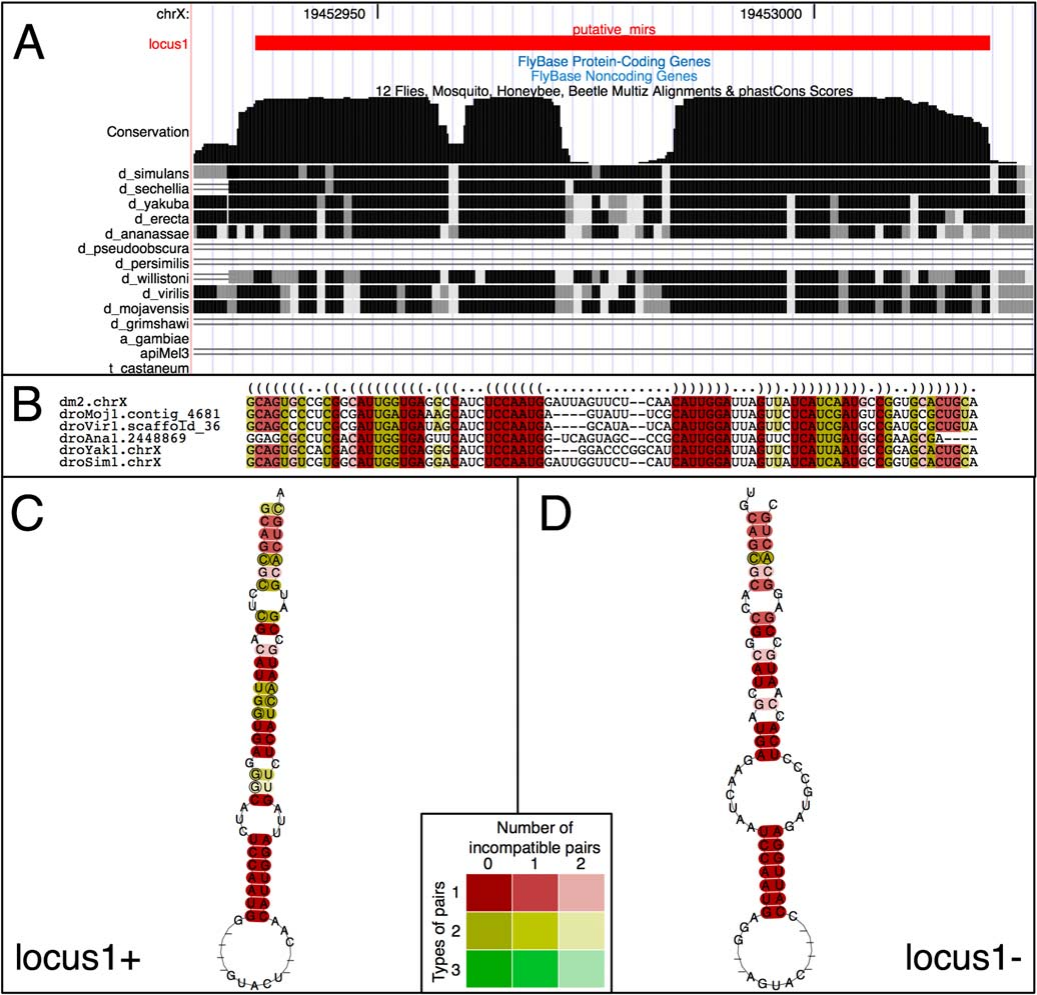
Example of a pre-miRNA predicted by RNAz. A) Locus1 shows the bimodal conservation pattern typical of a conserved miRNA hairpin structure in the phastcons track of the UCSC genome browser. Sequences from Drosophilids as distant as *D. mojavensis* contribute to the multiz alignment at this genomic position. B) Sequences from six species were chosen as input for RNAz and lend different levels of support to stabilizing selection of the predicted secondary structure prediction (first row). The color code indicates the number of different base-pairs (green = 3 pairs to red = 1 pair) and the number of pair incompatible with the predicted structure (dark color = 0 to faint color = 2) at each position. C, D) Locus1 is predicted to fold into symmetrical hairpins in both possible directions of transcription (color code as in B).

To identify novel miRNA precursor structures, we applied a stringent filtering strategy [38] to the RNAz output, selecting only RNAz predictions with i) a stem-loop structure exceeding 20 nt, and ii) high predicted thermodynamic stability, as indicated by a z-score of less than −3.5. To enrich further for true miRNA precursors, we selected sequences displaying the bimodal conservation pattern typical of microRNAs [6] by visually inspecting the “conservation” track in the UCSC genome browser (Figure 1). As a number of the known miRNA loci in the *Drosophila melanogaster* genome show high conservation across the full sequence of the pre-miRNA, we also included regions of constantly high conservation (Data S1, e.g. locus19, manual3). This strategy yielded 47 novel miRNA candidates, an example prediction is shown in Figure 1.

**Figure 2:**
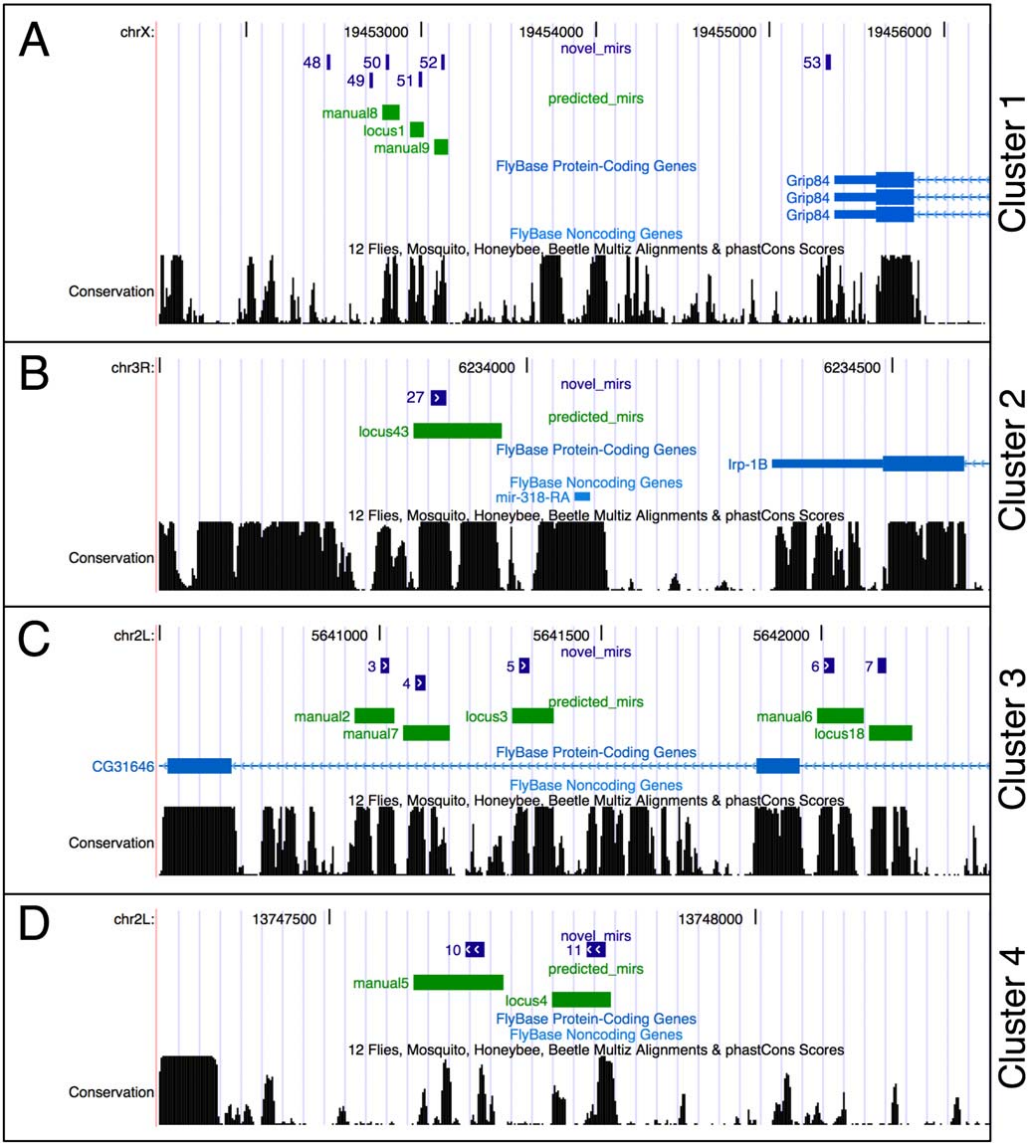
Prediction of four novel miRNA clusters. A) locus1, manual8, manual9 form a tight cluster downstream of the *Grip84* locus (green). Small RNAs detected by sequencing map to each of the novel miRNA loci (matches 50–52, blue). In addition, sequences map to two loci in the vicinity, giving rise to identical mature miRNAs (matches 48,49). Finally, a sixth locus is detected immediately downstream of *Grip84* (match 53). B) *mir-318*, located downstream of the *Irp1B* locus, clusters together with novel locus41 (green and small RNA sequence match 28, blue) C) Five novel miRNAs were predicted (green) and validated (blue) on either side of an exon of the CG31646 locus. D) Novel locus4 and manual5 (green) give rise to mature miRNAs (blue) and form cluster4.

In addition to the RNAz predicted stem-loop structures, we noticed the presence of several additional bimodally-conserved sequences close to some of the identified candidates. As many known miRNAs are found in clusters in animal genomes, we tested these sequences for their potential to form stable hairpin structures using RNAalifold [39]. In this way, nine additional sequences were added as “manual” predictions to the list of candidates, giving rise to several predicted clusters of miRNAs (Figure 2 and Data S1). In one of them, intriguingly miRNAs are predicted in two groups flanking an exon of the protein-coding gene CG31646 (Figure 2C, cluster 3).

### Experimental validation of predictions

Like protein-coding genes, miRNAs are often expressed only at specific developmental stages or in a subset of tissues. To test if mature microRNAs can be processed from primary transcripts originating from the predicted genomic sequences in a single experimental system, we cloned the genomic regions covering a number of putative novel miRNA loci and expressed them under them control of the actin 5C promoter by transfection into *Drosophila* S2 cells. The majority of candidate sequences had significant RNAz scores (>0.9) in both reading directions (53/56). The predicted hairpins were often symmetrical. These candidates were therefore expressed in both orientations to direct production of primary transcripts from both strands. Processing of these long primary transcripts was assayed by Northern blotting, and detected by hybridization with oligonucleotide probes directed against each arm of the predicted hairpin. Out of 33 candidates tested, 16 gave rise to a distinct small RNA (Table 1). Five of these proved to be endogenously expressed in S2 cells and could therefore be detected in untransfected cells.

**Table 1:** Validation of predicted miRNAs

ID	Chr	Start	Stop	Strand	processed in S2 cells	validated by sequencing
locus1	X	19452936	19453020	+	Y	Y
locus2	2L	6830211	6830286		N	N
locus3	2L	5641300	5641395	+	endogenous	Y
locus4	2L	13747762	13747830	−	Y	Y
locus6	3L	10312110	10312214		N	N
locus7	3R	24628381	24628452	−	endogenous	N
locus8	3R	6842228	6842321		N	N
locus9	3R	10816276	10816378		N	N
locus11	X	174206	174307	−	Y	Y
locus12	X	1682883	1682986	−	Y	Y
locus13	3R	21414577	21414677	−	Y	Y
locus14	3R	20313365	20313451		N	N
locus15	X	15859994	15860084	−	Y	Y
locus17	2L	243033	243125	−	N	Y
locus18	2L	5642109	5642206	+	Y	Y
locus20	2L	6902058	6902149	+	Y	Y
locus23	2L	15479411	15479482		N	N
locus26	2R	1705689	1705773	+	endogenous	N
locus27	2R	3456100	3456167		N	N
locus39	3R	11559905	11559991		N	N
locus40	3L	10153900	10153966		N	N
locus43	3R	6233847	6233967	+	Y	Y
locus45	3L	17699260	17699368		N	N
locus46	3R	4357258	4357332		N	N
locus47	3R	121092	121163	+	n.a.	Y
locus48	3R	2602056	2602170	+	n.a.	Y
manual1	X	16362566	16362654		N	N
manual2	2L	5640941	5641032	+	N	Y
manual3	2L	6829891	6829990	+	endogenous	N
manual4	X	16362736	16362841		N	N
manual5	2L	13747600	13747705	−	Y	Y
manual6	2L	5641992	5642096	+	N	Y
manual7	2L	5641054	5641158	+	endogenous	Y
manual8	X	19452776	19452876	+	N	Y
manual9	X	19453074	19453153	+	Y	Y

The processing of primary transcripts ectopically expressed in S2 cells under the control of the actin 5C promoter was analyzed by Northern blot using probes designed against both arms of the predicted hairpins. In 16 cases, mature sequences could be detected (column 6, Y), five of which were present in untransfected S2 cells. For the remaining predictions, no processing was observed (column 6, N). In most cases, small RNAs corresponding to the probed sequences could also be detected in published small RNA libraries (13/16). Six miRNAs either not assayed (colum 5, n.a.) or not detected by Northern blot could be validated, too. No matching sequences were found in the libraries for locus7, locus25 and manual3, which can be detected as endogenous transcripts (column 6, endogenous) by Northern blot.

**Figure 3:**
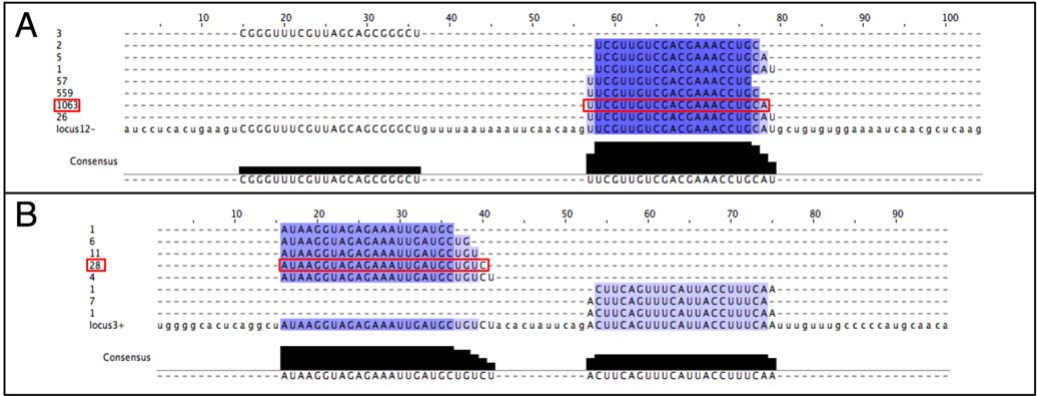
Examples of small RNA sequences mapping to predicted miRNA loci. Small RNA sequences recovered by sequencing [12] were mapped to the predicted loci using Megablast. A) locus12 is transcribed from the ‘–’ strand and multiple overlapping sequences map to its coordinates. Numbers on the left indicate how often each sequence was identified in the small RNA libraries. The most abundant species (red box) most likely represents the mature miRNA. B) locus3 maps to the ‘+’ strand. Details for all predicted loci is available as supplemental data.

While this experimental analysis was nearing completion, high-throughput sequencing data was published for small RNA libraries cloned from ten different samples, including the major developmental stages of *Drosophila melanogaster* development [12] [GEO:GSE7448]. This allowed us to compare our experimental data with an independent unbiased global expression analysis. We mapped these small RNAs to the non-repetitive part of the *Drosophila* genome and used RNAfold [40] to screen for sequenced RNAs falling into regions predicted to fold into an extended hairpin structure. This way, 53 novel miRNA loci were identified. 19 of these corresponded to one of our 56 microRNA predictions (Table 1). 13 of these were miRNAs independently validated by our Northern blot analysis. In each case small RNA sequences mapped only to a single strand, corresponding to the previously identified direction of transcription. In agreement with the stepwise processing of pre-miRNA precursors, 21–25 long oligonucleotides often originated from both arms of the hairpin, albeit at different frequencies: the mature miRNA outnumbers its cognate miRNA* sequence with a ratio up to 1713:3 (locus12, Figure 3A).

**Figure 4:**
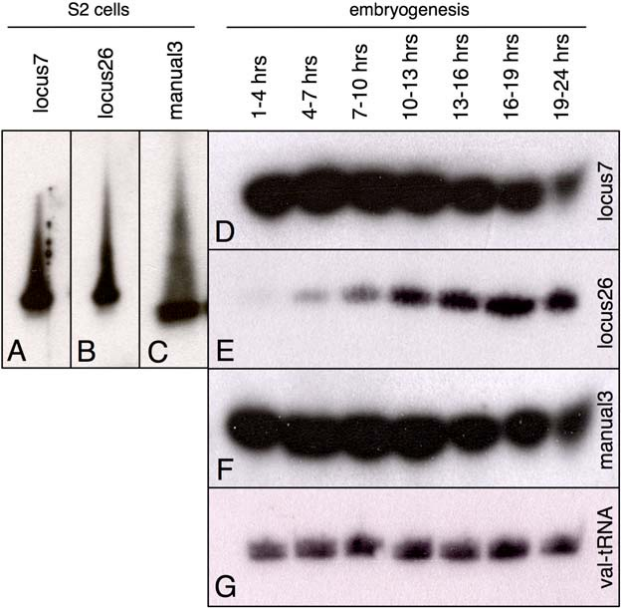
Northern blot validation of three miRNAs not found in small RNA libraries. Three predicted miRNAs were not represented in the small RNA libraries, yet could be detected by Northern blot analysis. A–C) Endogenous small RNAs were detected with probes directed against locus7, locus25 and manual3 in total RNA extracted from untransfected S2 cells. D–F) All three transcripts were also detected in RNA from different embryonic stages (time after egg-laying). F) A probe against valine tRNA was used as a loading control.

We have used available sequence data from ten different anatomies or developmental stages to validate and complement our predictions, pushing the total number of annotated miRNAs to 146 - beyond the previous expectation of ∼120. Although there is considerable overlap between the prediction/validation and sequence data sets, comparing them showed that neither set is complete. 19/53 of the novel candidates (38.2%) were identified both computationally and by sequencing. In addition, the small RNA libraries provided evidence for 31 additional loci that we had not predicted. Three predicted miRNAs (locus26, locus7 and manual3) could be detected in untransfected S2 cells and in developing embryos by Northern blot analysis (Figure 4), but were not found in the sequencing data. Locus7 and manual3 are expressed strongly in early stages and decline in abundance toward the end of embryogenesis, whereas locus26 expression begins low and increases during mid- to late embryogenesis (Figure 4E–G). In aggregate, this illustrates that any single approach aimed at identifying miRNAs continues to underestimate the miRNA complement of the *Drosophila melanogaster* genome.

### Mature miRNA sequences

**Figure 5:**
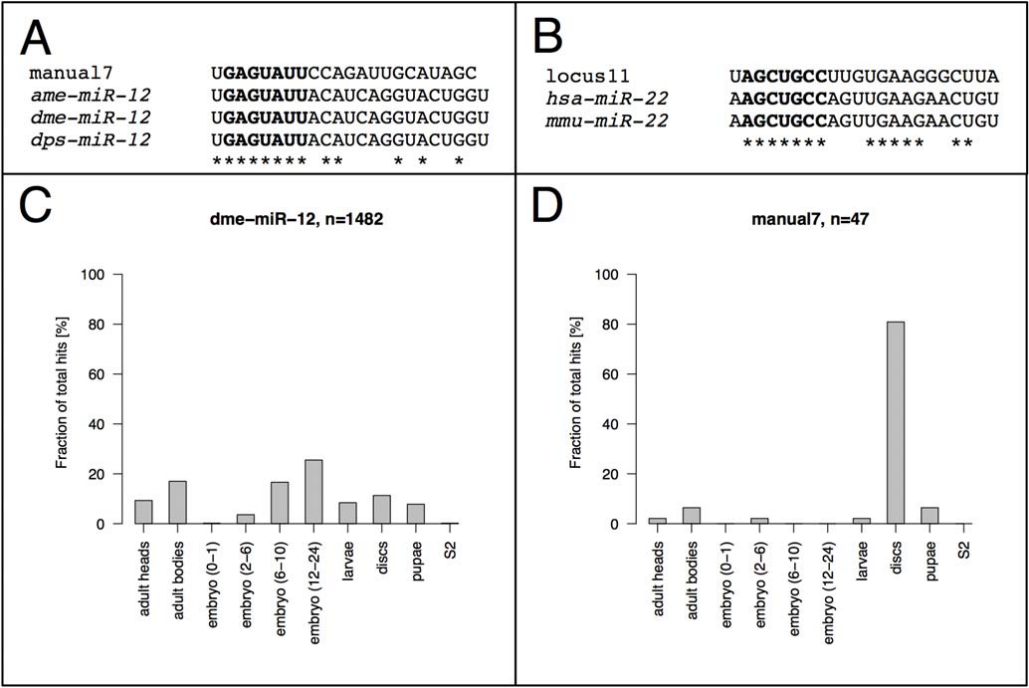
miRNAs sharing seed sequences. A) The novel miRNA manual7 contains the same seed sequence as the D. melanogaster *mir-12* miRNA and its orthologs. B) Sequence similarity between the novel locus11 and vertebrate *miR-22* extends beyond the seed region, hinting at a possible common ancestry of these miRNAs. C) The mature sequence of *Drosophila mir-12* was detected more than 1400 times in the small RNA libraries in samples from all developmental stages/tissues with the exception of the early embryo. D) The most abundant sequence mapping to the novel manual7 locus is distributed differently over the 10 sequenced samples, with RNA from discs contributing >80% of hits.

By identifying the small RNA sequences detected most frequently in the small RNA libraries, we could pinpoint the mature microRNAs. Interestingly, eight of the novel miRNAs share identical seed sequences with known miRNAs (Table S1). The first eight bases of the mature manual7 sequence correspond to that of another *Drosophila melanogaster* miRNA, *mir-12*, suggesting that both miRNAs could regulate a similar set of targets (Figure 5A). While *mir-12* was detected in samples from all developmental stages/tissues with the exception of the early embryo (Figure 5B), mature manual7 is highly enriched in imaginal disks (80.9% of all sequence hits, Figure 5C), where it might tighten the repression of the common set of targets specifically at this stage of development. Similarly, “seed paralogs” were found for *Drosophila mir-279, mir-285* and *mir-286*. Also, several of the newly identified miRNAs have unique seeds in this model organism, but similar seeds are found in miRNAs of other species, including vertebrates (Table S1).

In some cases, the alignment between novel and previously described miRNAs extends well beyond the seed sequences, suggesting a common evolutionary ancestry. Locus11 shares e.g. 14 out of 22 bases with vertebrate *mir-22*, a microRNA highly conserved in vertebrates but until now lacking a *Drosophila* ortholog (Figure 5D, Table S1).

### Clusters

**Figure 6:**
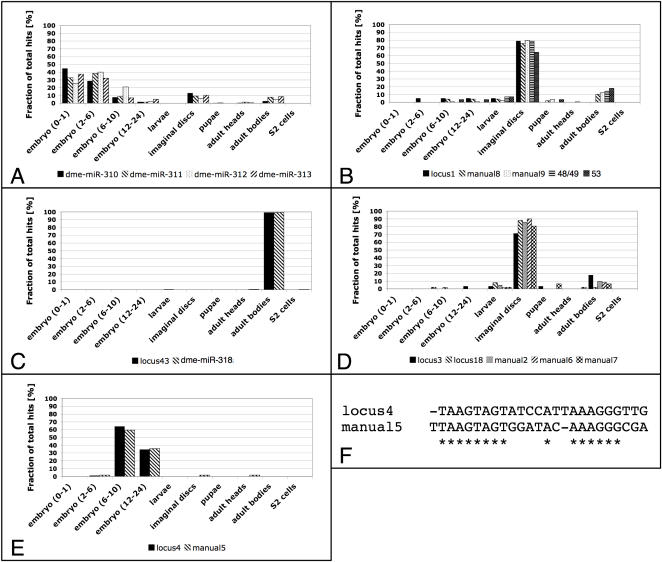
Four novel clusters encoding tightly co-expressed miRNAs. Though the individual number of sequence hits recovered in the small RNA libraries differs greatly between different miRNAs, the relative abundance in the different sequenced samples is highly correlated for miRNAs originating from the same cluster. A) Similar fractions of the four members of the Drosophila *miR-310-313* cluster are recovered in embryonic, imaginal disc and adult body samples, but virtually absent from the other samples. B–E) Members of the four novel miRNA clusters are distributed equally tightly over the small RNA libraries, demonstrating tight co-regulation. F) The mature sequences of locus4 and manual5 are highly similar and map to neighboring genomic coordinates (E), suggesting a duplication event that gave rise to two independent loci.

Several of the predicted miRNAs were found in distinct experimentally validated genomic clusters (Figure 2). To investigate whether miRNAs from each genomic cluster are likely to be processed from the same primary transcript, we compared the relative frequencies of the mature miRNA from these validated clusters in different RNA libraries (Figure 6B–E). Although the absolute number of sequence hits varies between the different members of the same cluster (e.g. 19 hits for locus1 and 162 hits for manual9 in cluster1), their relative abundance in different sequenced tissues is remarkably similar. Comparable results were obtained with a known miRNA cluster (*mir-310, mir-311, mir-312 and mir-313*, Figure 6A), suggesting that the novel clusters may also encode polycistronic transcripts. While the miRNAs of cluster1, cluster2 and cluster3 seem to be unrelated in sequence, cluster4 gives rise to two very similar mature sequences (Figure 6F). Manual5 carries an additional U at its 5′ end compared to locus4, giving rise to a novel putative seed sequence. As both mature sequences clearly outnumber any other matching oligonucleotides mapped to the same locus (Table 1), locus4 and manual5 most likely represent distinct, recently diverged microRNAs.

### Conclusions

By combining RNAz, a “generic” tool to predict non-coding RNAs, with specific filters to identify miRNA precursors, we have taken advantage of the recent sequencing of a large number of insect species. The fact that 22 out of our 56 predictions (39%) could be verified by Northern blot analysis or sequencing, but were missed in previous studies, demonstrates the power of this approach.

The degree of sequence conservation of miRNA precursors between related species varies widely (compare e.g. the phastcons tracks for manual5 and locus48, Data S1). The genome-wide alignments of 9 or 15 insects enabled us to select suitable sequences for each candidate region separately, allowing for sequence variation of rapidly as well as slowly diverging miRNAs. This approach is likely to prove useful for the analysis of other animal genomes, as more related genomes are sequenced.

## Materials and Methods

### Computational miRNA predictions

Genome-wide alignments of 9 (multiz9) and 15 (multiz15) insect species were downloaded from the UCSC genome browser. Starting from the “most conserved” track for each alignment generated by the Phastcons program, we padded the genomic coordinates by 25 bps on both sides. Neighboring sequences closer than 50 bps to each other were combined into a single region. All sequences overlapping annotated genes according to the “Flybase genes” and “Refseq genes” tracks of the UCSC genome browser were removed.

Input windows were prepared with the rnazWindow.pl script of the RNAz package with default settings. This resulted in alignments with a maximum length of 200 bps (longer alignments were split into overlapping windows of 120 bps with a step size of 40) and a minimum number of aligned sequences (>50% identity) from at least 4 different species. RNAz (version 1.0) was used to score the alignments and hits with a probability score p>0.9 were stored. Overlapping predictions were joined using the rnazCluster.pl script of the RNAz package and filtered for hairpin length (>20 bps, total length of the prediction >65 bps), thermodynamic stability (z-score <−3.5). The remaining predictions were uploaded to a local installation of the UCSC genome browser and their multiz15 phastcons conservation profile inspected manually.

### Cloning and expression of putative miRNAs in S2 cells

Amplicons of ca. 0.5 kb length, centered on the predicted pre-miRNA were generated by PCR and cloned into vector pAc5.1B (Invitrogen). S2 cells were grown in the absence of serum, transfected using Cellfectin (Invitrogen) and incubated for 3 days at 25°C. Total RNA was extracted from transfected or control cells, as well as from staged collections of *Drosophila* embryos using Trizol (Invitrogen) and precipitated with isopropoanol overnight at −20°C. 15 µg of total RNA was separated on a 15% denaturing polyacrylamide gel and blotted using a chemical cross-linking procedure [41]. Oligonucleotide probes were designed separately against each full arms of the predicted hairpin and end-labeled with ^32^P-ATP using T4 polynucleotide kinase (NEB).

### Mapping small RNA sequencing data to predicted loci

Sequences detected in Drosophila small RNA libraries [12] were obtained from GEO [GEO:GSE7448] and mapped to the putative pre-miRNA sequences using megablast [42] with wordsize 14 and a score cutoff of 16. Only perfectly matched sequence matches were retained. The resulting alignments were displayed with Jalview [43].

miRNA loci were identified from the small RNA libraries *de novo* by moving a 110 bp window (stepsize 5) over all bases recovered at least 10 times. RNAfold [40] was used to predict the secondary structure of each sequence window and hairpins with more than 30 base-paired nucleotides and bulges of less than 15 nucleotides were retained. Repetitive regions or sites overlapping with annotated loci were removed.

## Supporting Information

Data S1 Pdf document containing information about all validated novel miRNAs, including RNAz alignment and structure models.
(2.17 MB PDF)

Table S1 Genome data on 53 new miRNAs. Information about all 53 validated novel miRNAs, including their genomic coordinates (genome version 5) and their mature sequences.
(0.02 MB PDF)
