# Supplementary material for: Identification of Novel Drosophila melanogaster MicroRNAs
Source: PLoS One. 2007 Nov 28;2(11):e1265. doi: 10.1371/journal.pone.0001265 (PMC2082411; doi:10.1371/journal.pone.0001265)

miRNA 1

chr2L : 243055 - 243076 Transcribed strand: -

predicted as: locus17

Validated: detected in small RNA sequencing data

Mature sequence: UAAGCGUAUAGCUUUUCCCCUU

conservation profile

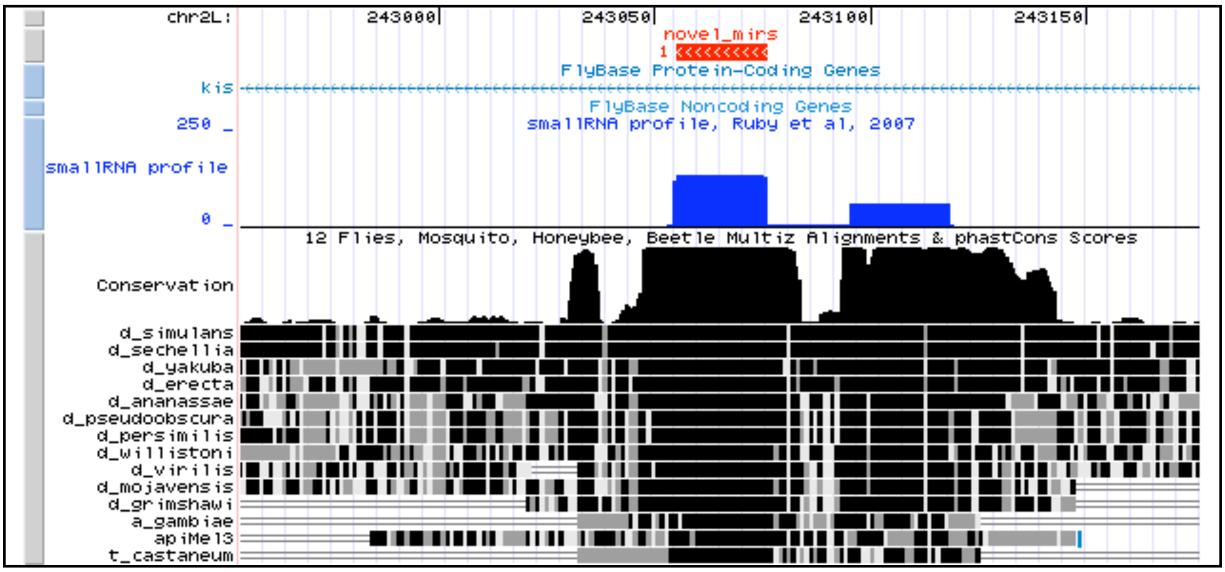

genomic context

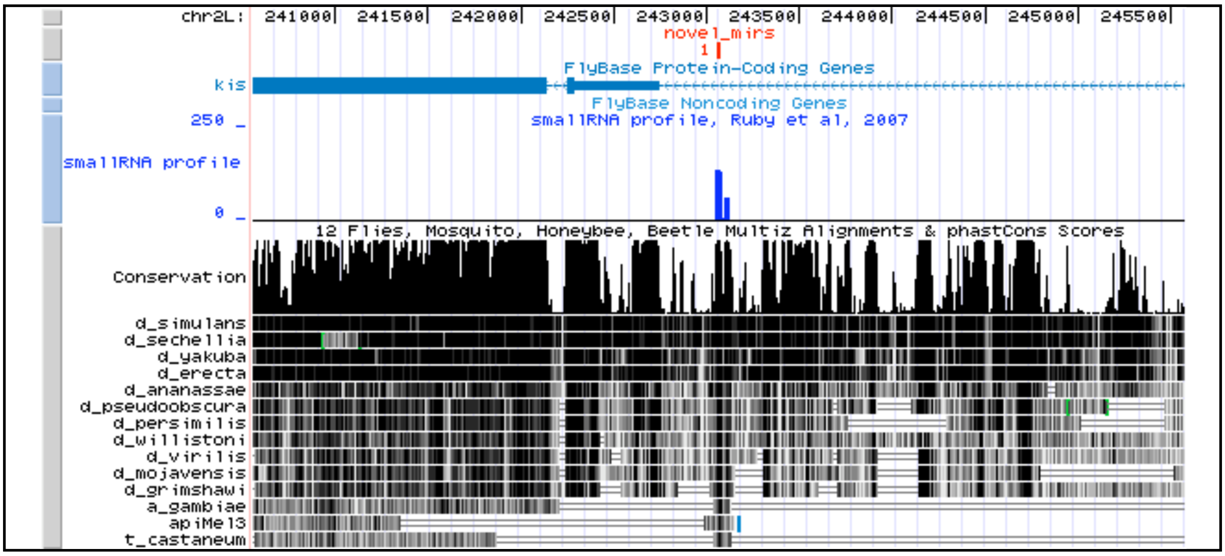

rnafold / alifold prediction

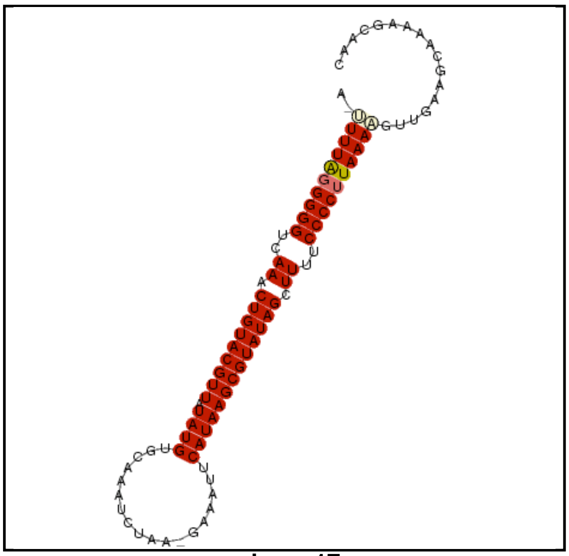

locus17-

Hits in RNA libraries

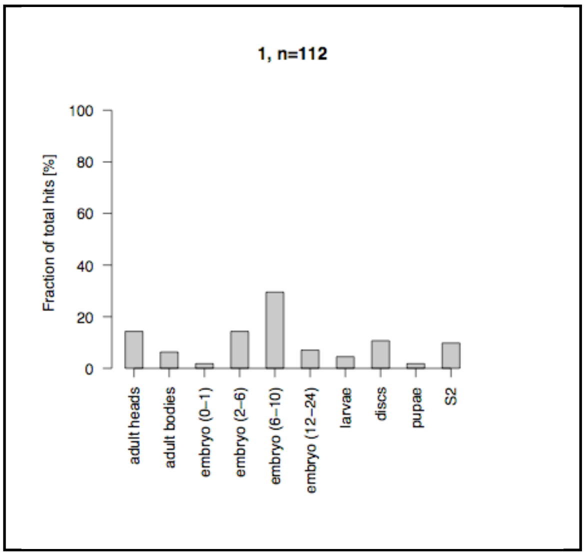

miRNA 2

chr2L : 857596 - 857617 Transcribed strand: +

predicted as:

Validated: detected in small RNA sequencing data

Mature sequence: UUUGUUCGUUUGGCUUAAGUUA

conservation profile

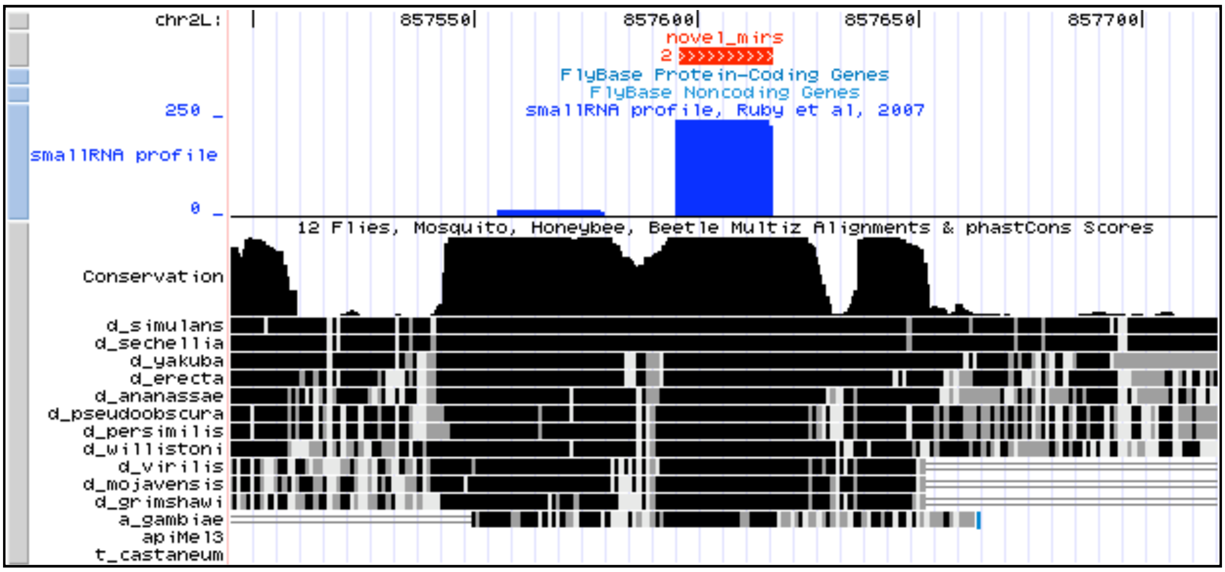

genomic context

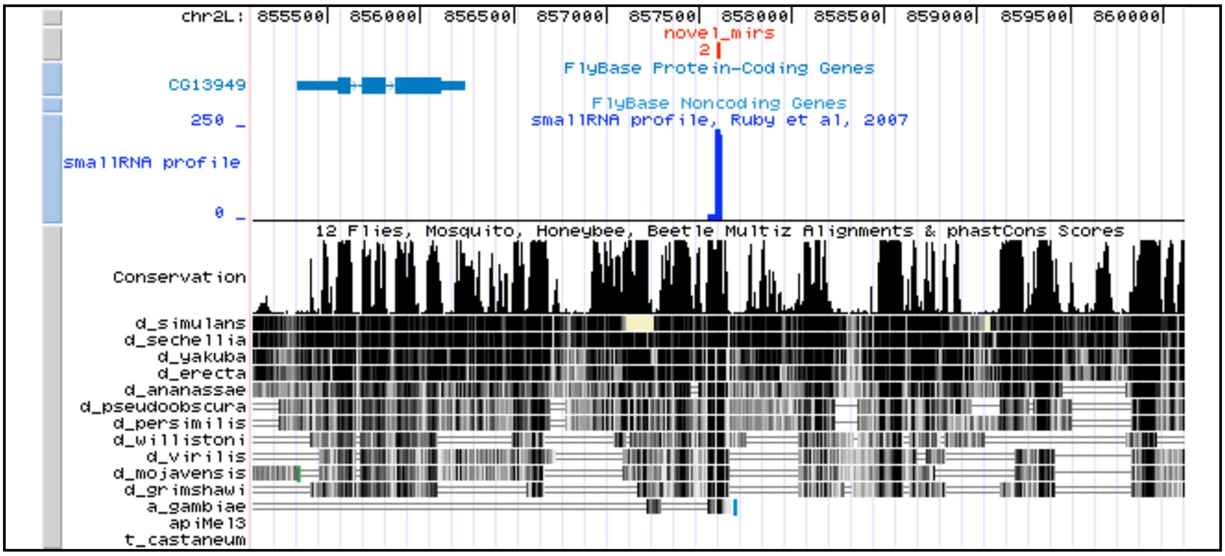

rnafold / alifold prediction

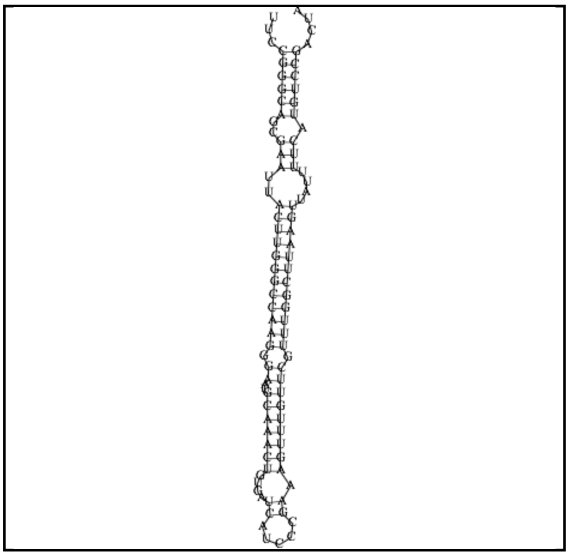

Hits in RNA libraries

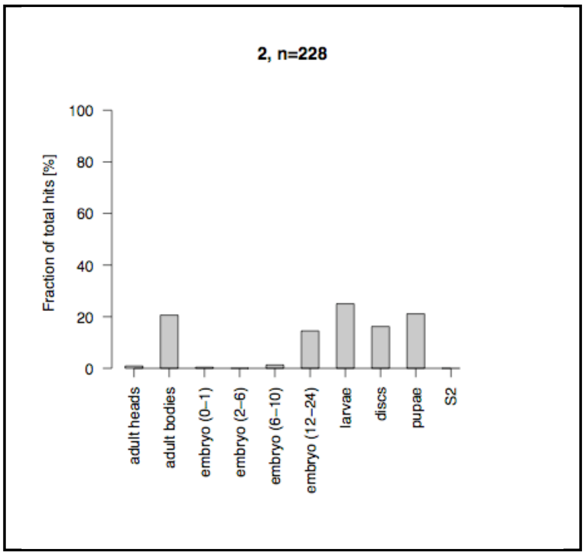

miRNA 3 chr2L : 5641000 - 5641021 Transcribed strand: + **cluster3**

predicted as: manual2

Validated: detected in small RNA sequencing data

Mature sequence: UUGUCAUCGGGGGUAUUAUGAA

conservation profile

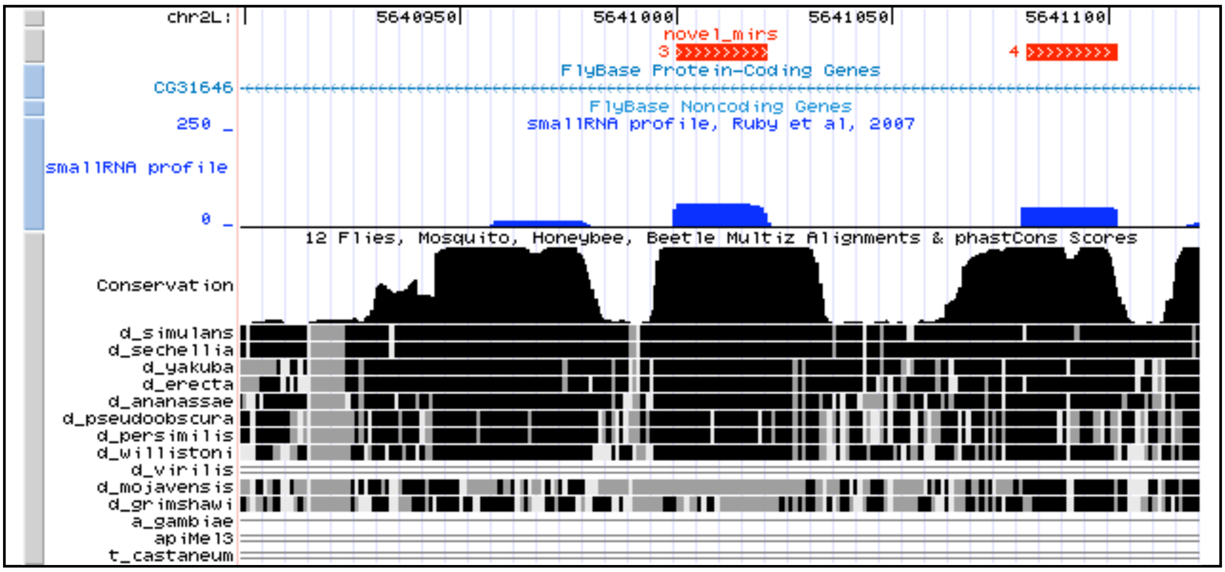

genomic context

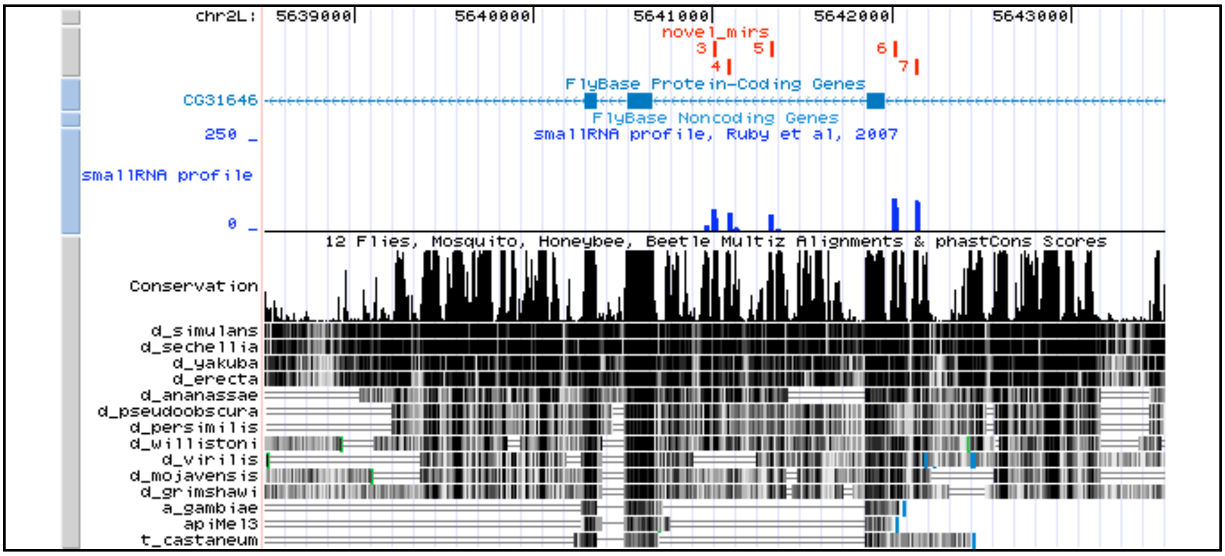

rnafold / alifold prediction

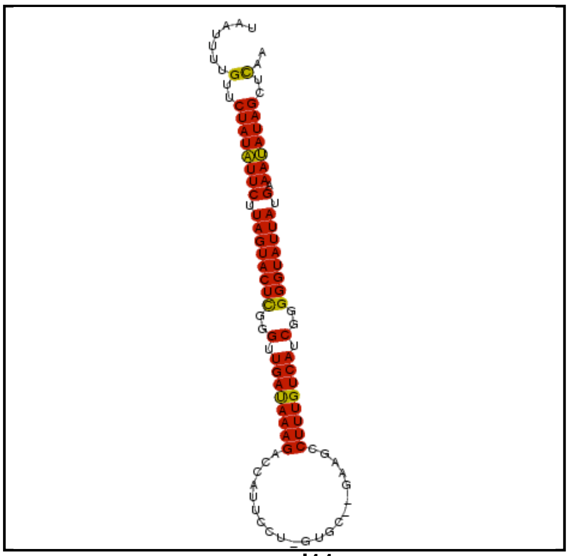

manual11+

Hits in RNA libraries

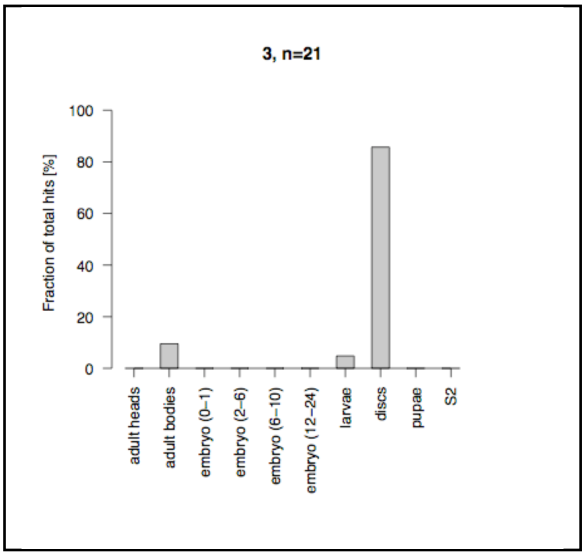

miRNA 4 chr2L : 5641081 - 5641102 Transcribed strand: + cluster3

predicted as: manual7

Validated: detected in small RNA sequencing data

Mature sequence: UGAGUAUUCCAGAUUGCAUAGC

conservation profile

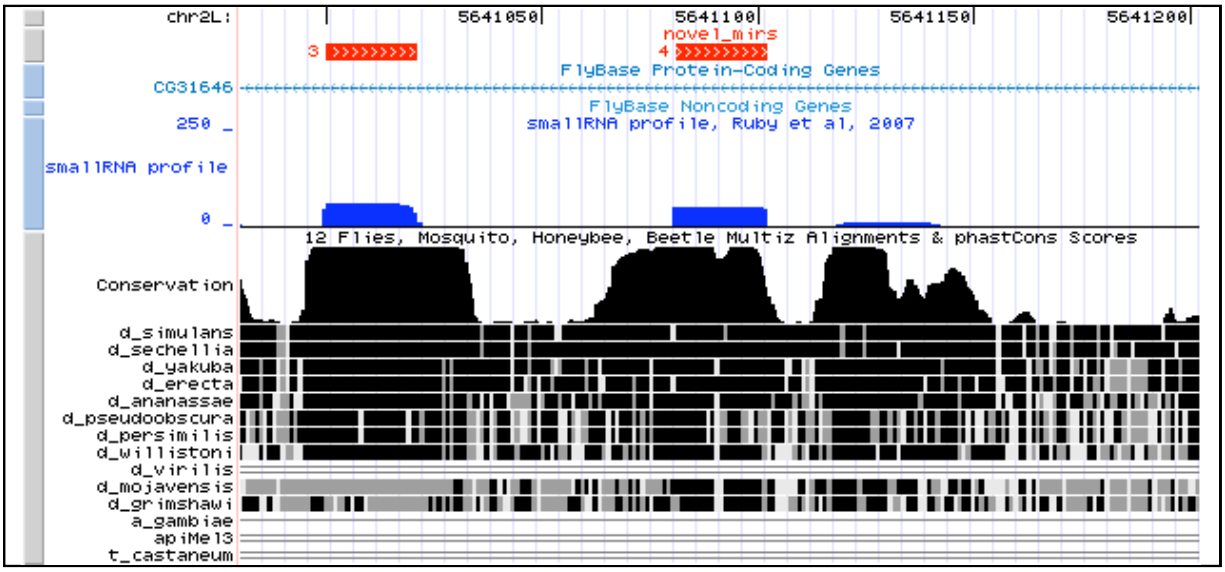

genomic context

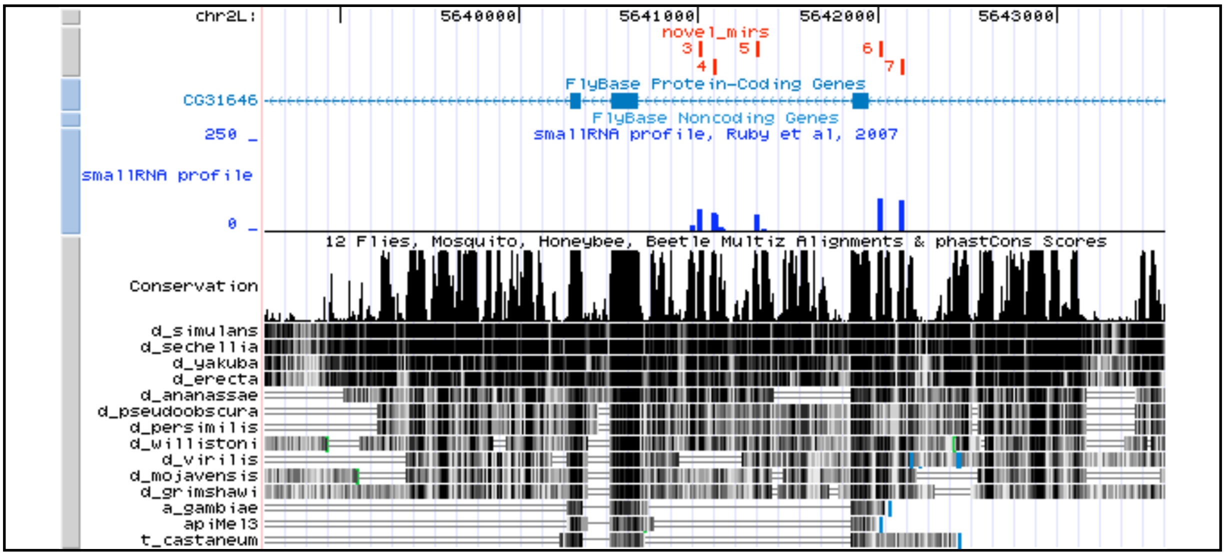

rnafold / alifold prediction

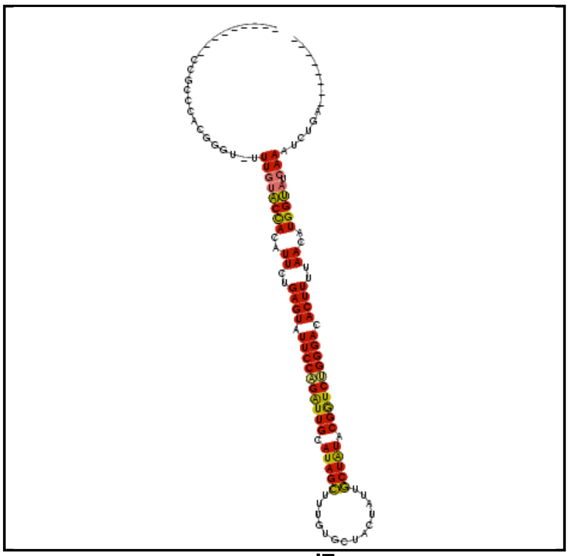

manual7+

Hits in RNA libraries

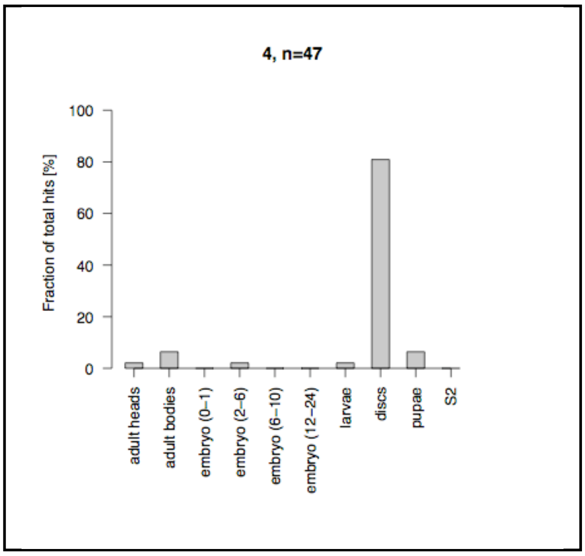

miRNA 5 chr2L : 5641316 - 5641339 Transcribed strand: + cluster3

predicted as: locus3

Validated: detected in small RNA sequencing data, validated by Northern blot

Mature sequence: AUAAGGUAGAGAAAUUGAUGCUGUC

conservation profile

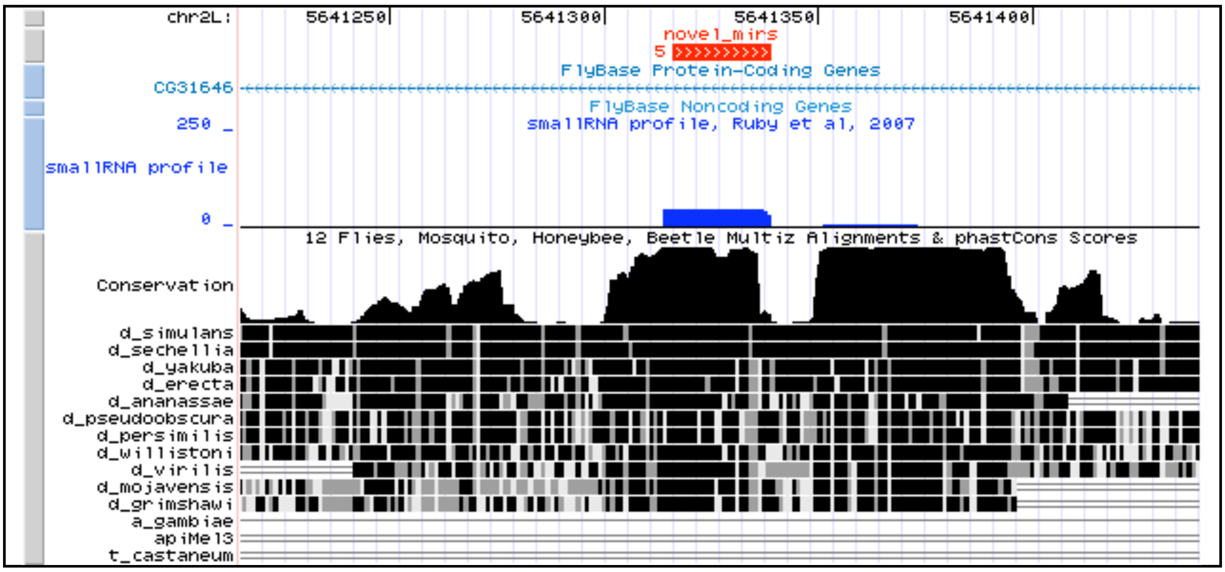

genomic context

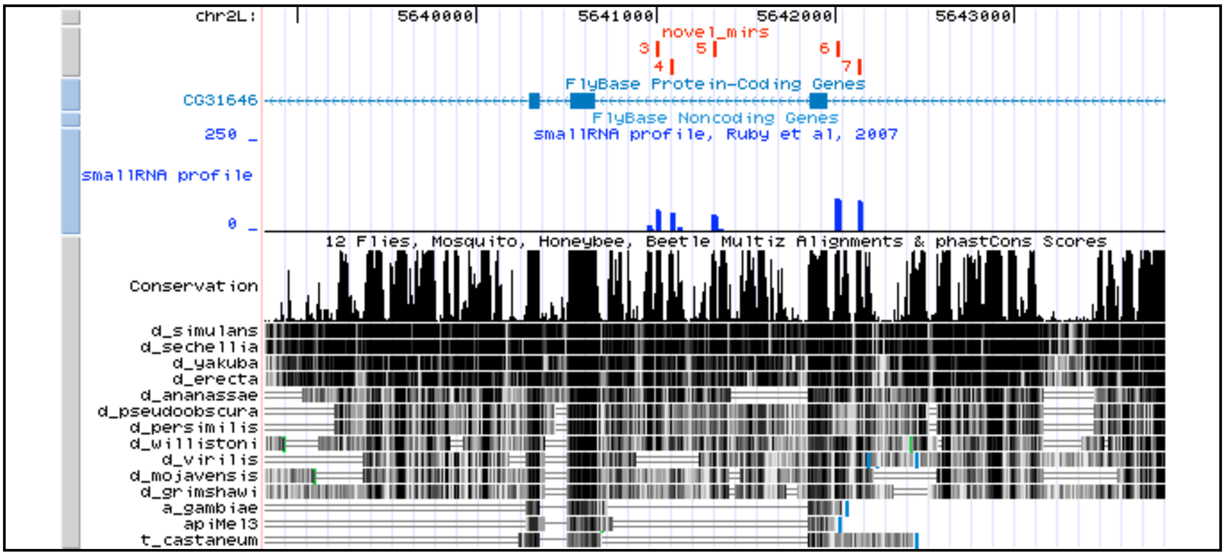

rnafold / alifold prediction

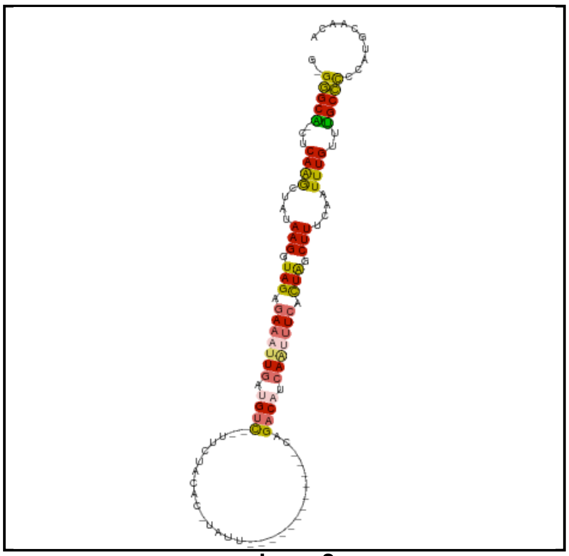

locus3+

Hits in RNA libraries

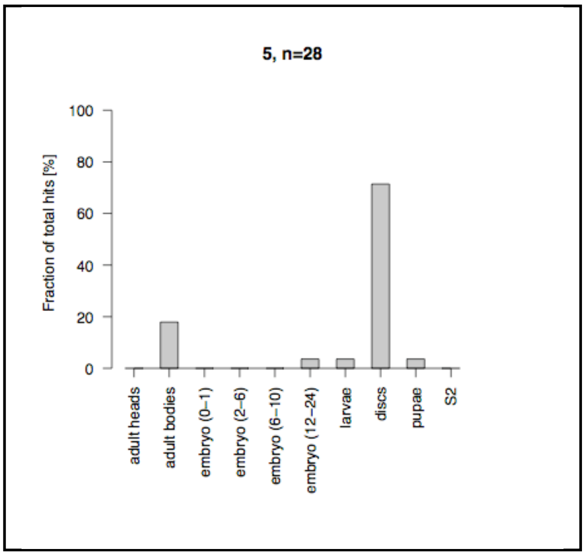

miRNA 6 chr2L : 5642005 - 5642028 Transcribed strand: + cluster3

predicted as: manual6

Validated: detected in small RNA sequencing data

Mature sequence: ACAAGGUAAAUAUCAGGUUGUUUC

conservation profile

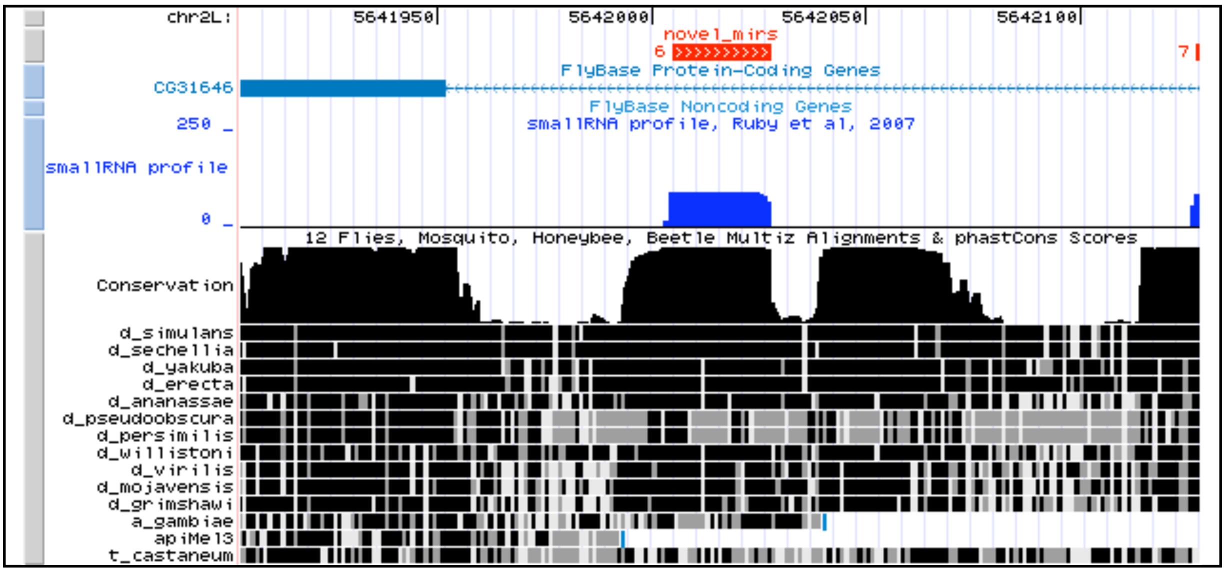

genomic context

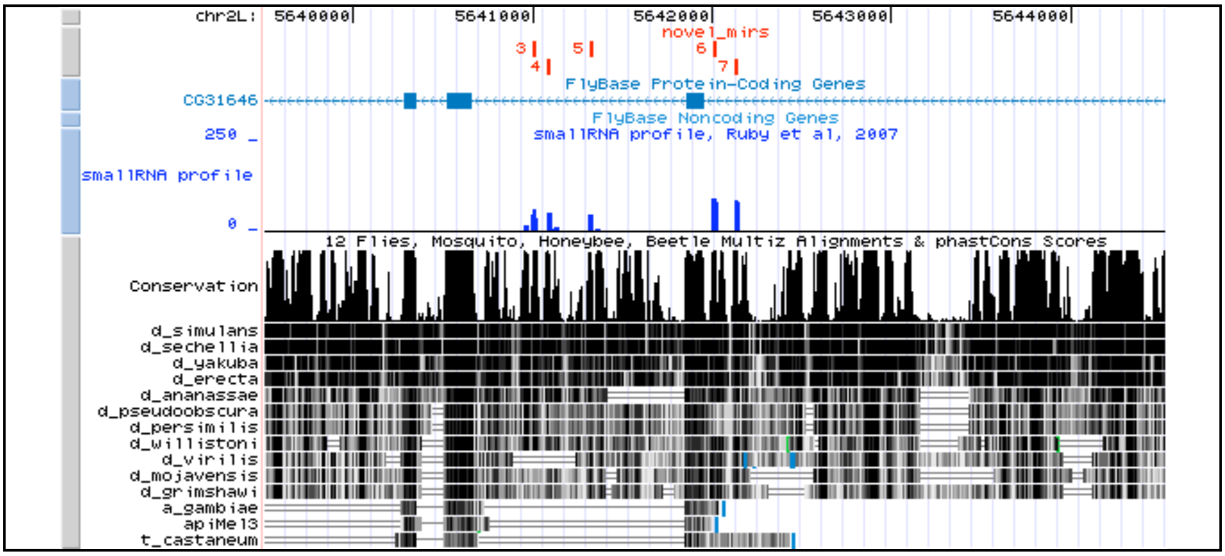

rnafold / alifold prediction

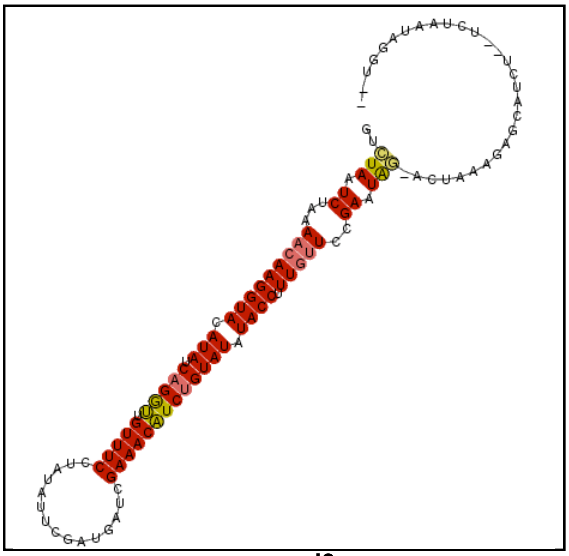

manual6+

Hits in RNA libraries

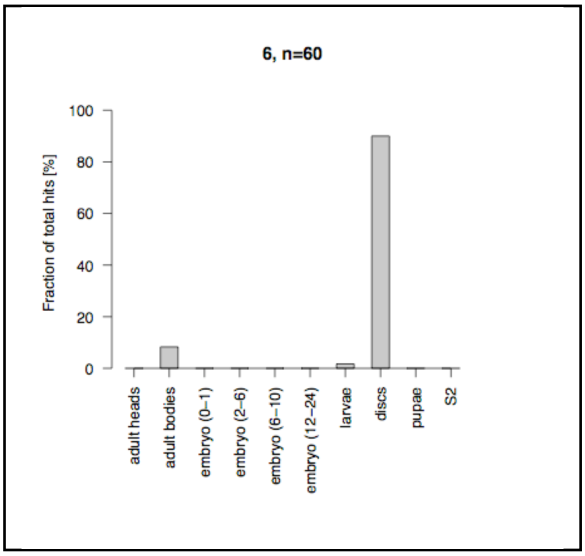

miRNA 7 chr2L : 5642127 - 5642148 Transcribed strand: + cluster3

predicted as: locus18

Validated: detected in small RNA sequencing data, validated by Northern blot

Mature sequence: UUAGAAUAGGGGAGCUUAACUU

conservation profile

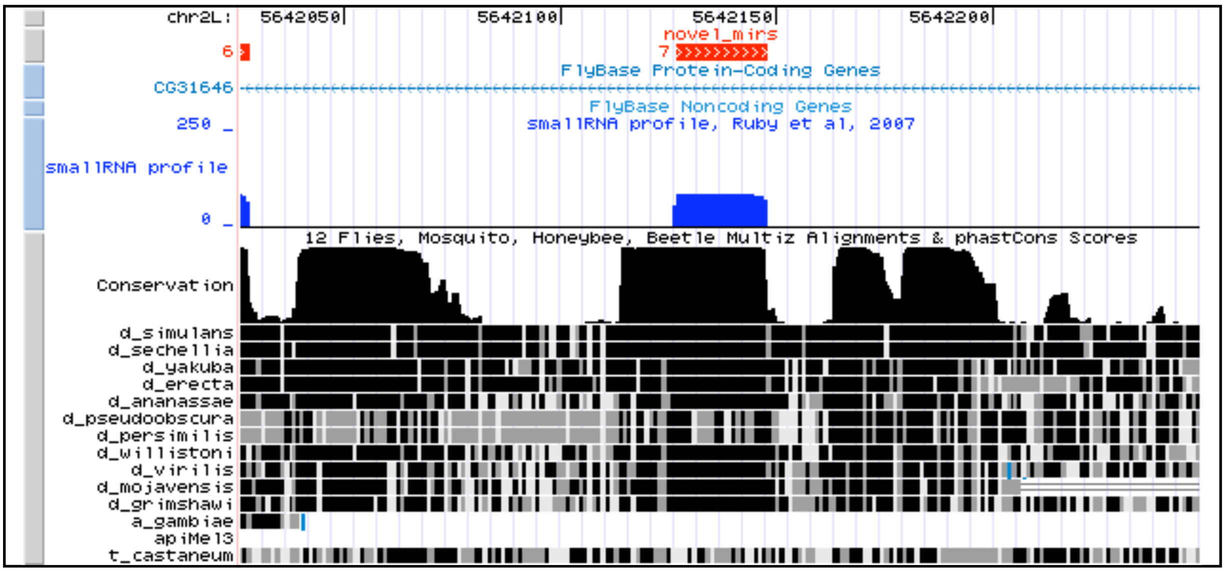

genomic context

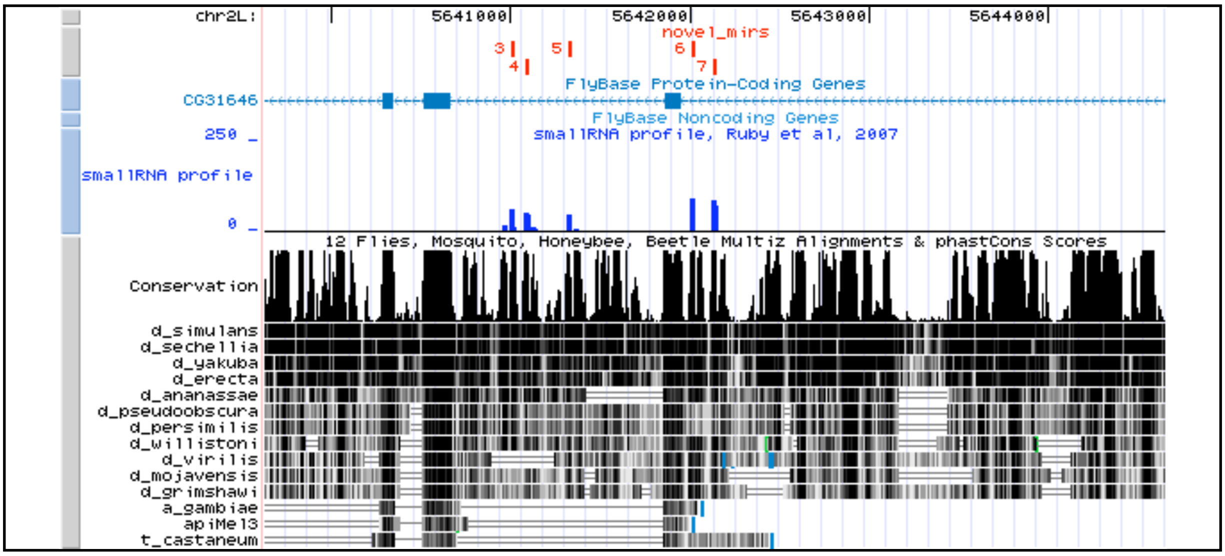

rnafold / alifold prediction

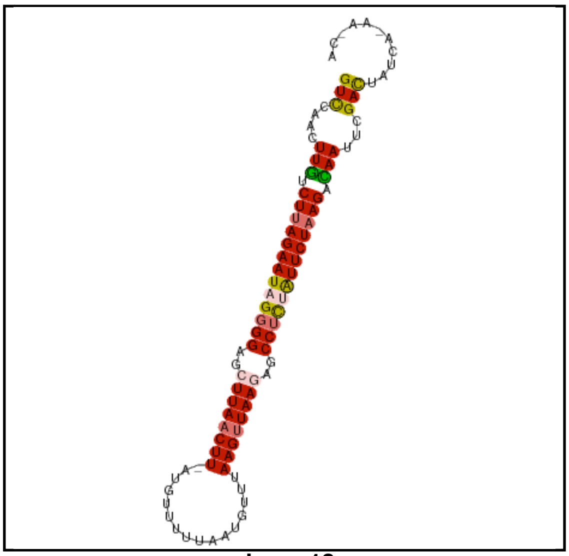

locus18+

Hits in RNA libraries

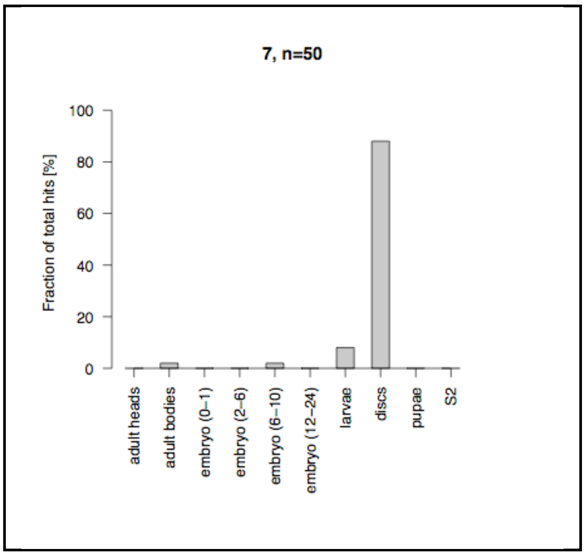

miRNA 8

chr2L : 6829891 - 6829990 Transcribed strand: +

predicted as: manual3

Validated: validated by Northern blot only

Mature sequence: n.d., sequence matched by Northern probe: GCGUGGCGAAUUAGAAUGUUGUCAAAUUGUUAU

conservation profile

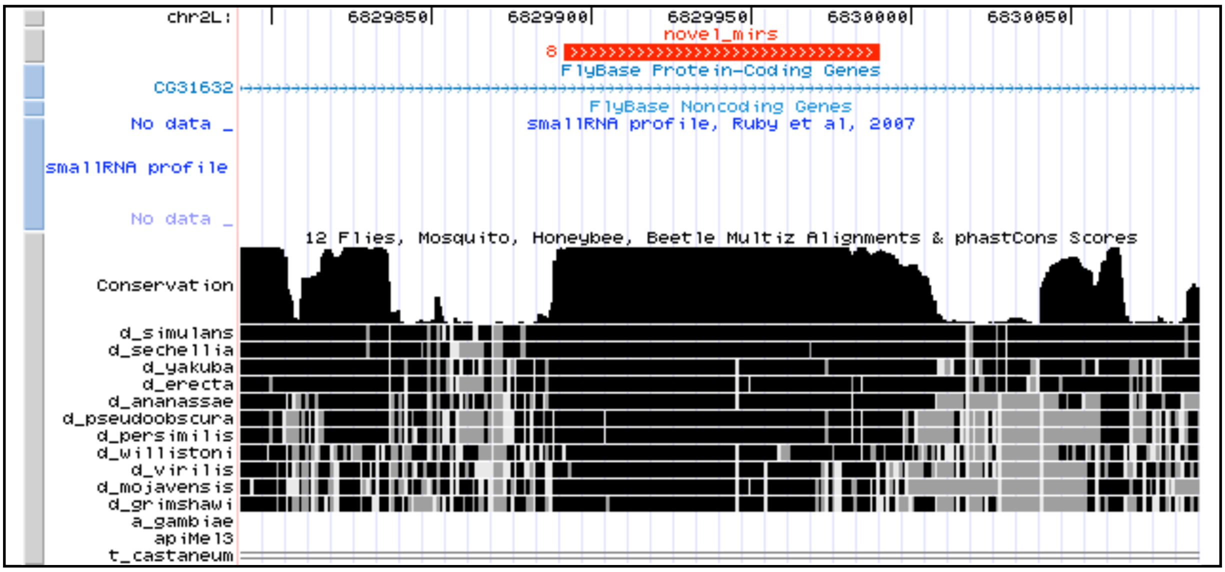

genomic context

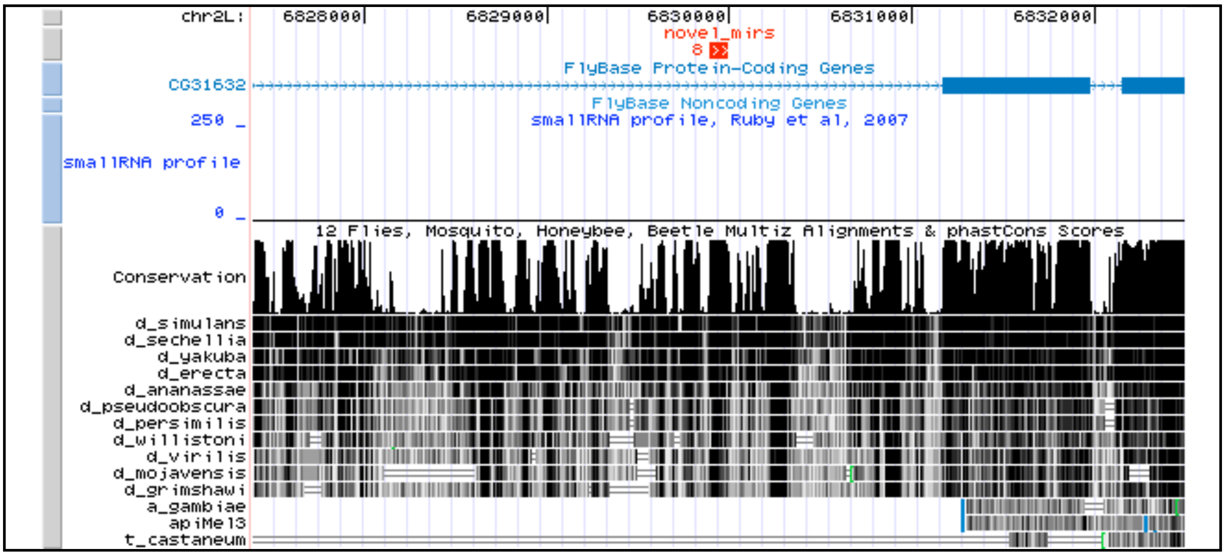

rnafold / alifold prediction

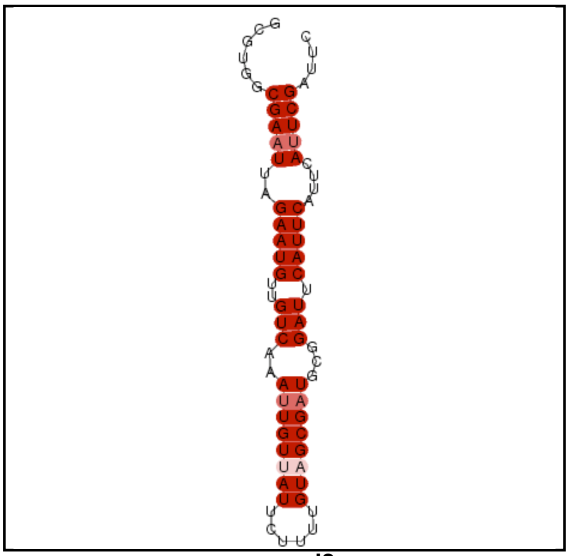

manual3+

Hits in RNA libraries

miRNA 9 chr2L : 6902077 - 6902099 Transcribed strand: +

predicted as: locus20

Validated: detected in small RNA sequencing data, validated by Northern blot

Mature sequence: UCAAUUCCGUAGUGCAUUGCAG

conservation profile

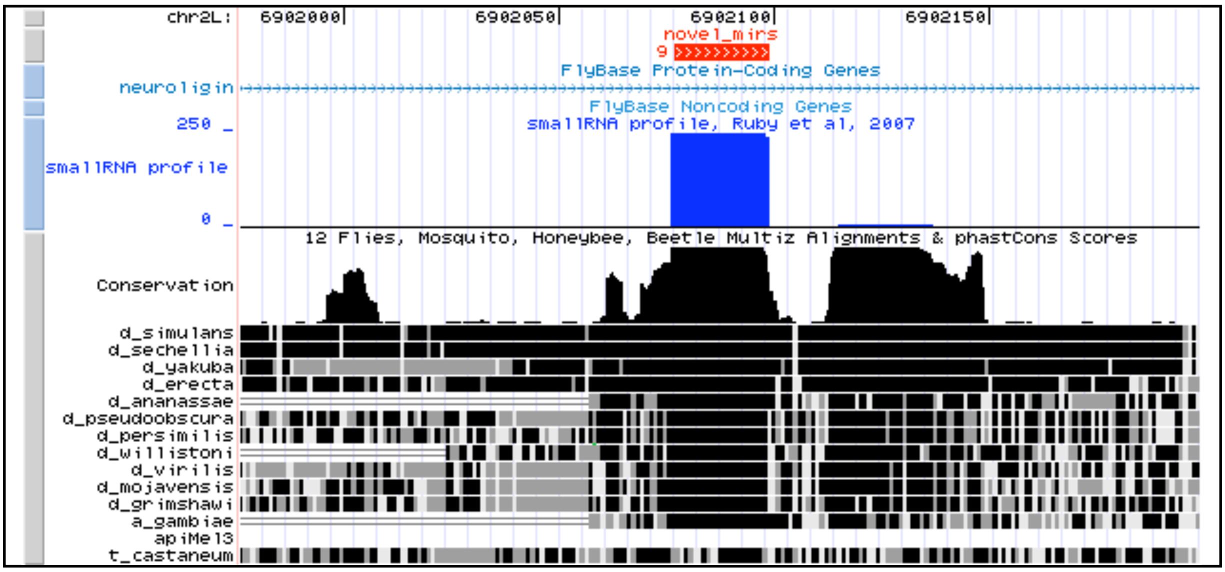

genomic context

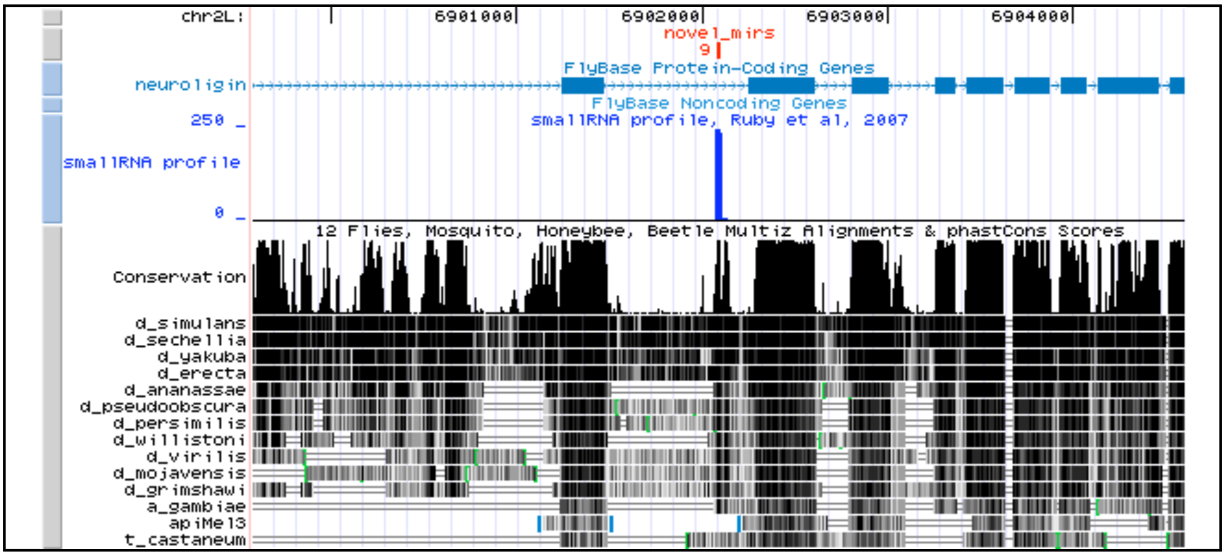

rnafold / alifold prediction

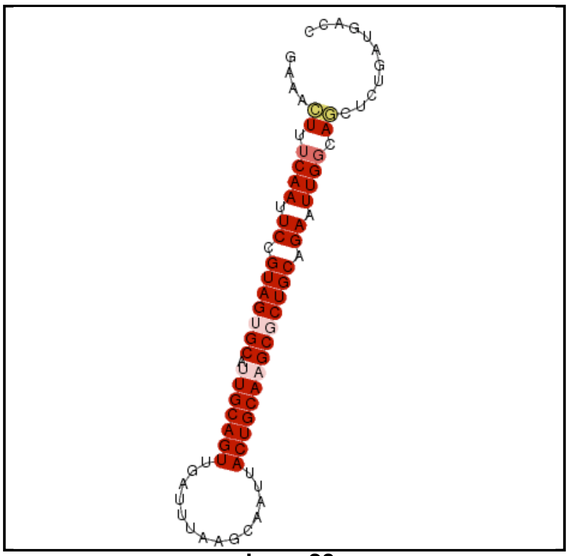

locus20+

Hits in RNA libraries

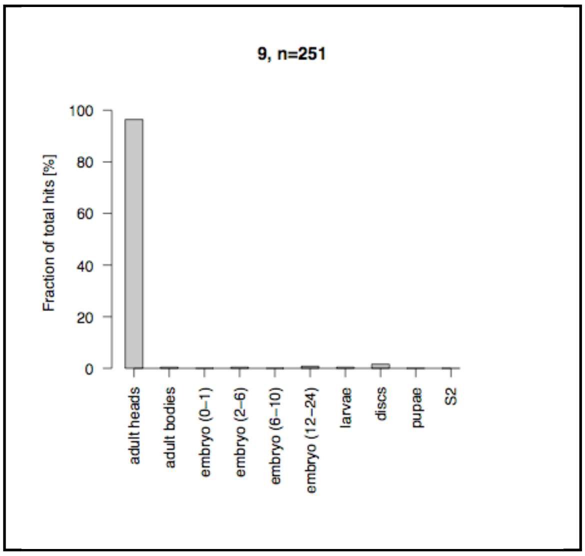

miRNA 10

chr2L : 13747660 - 13747683 Transcribed strand: - cluster4

predicted as: manual5

Validated: detected in small RNA sequencing data, validated by Northern blot

Mature sequence: UUAAGUAGUGGAUACAAAGGGCGA

conservation profile

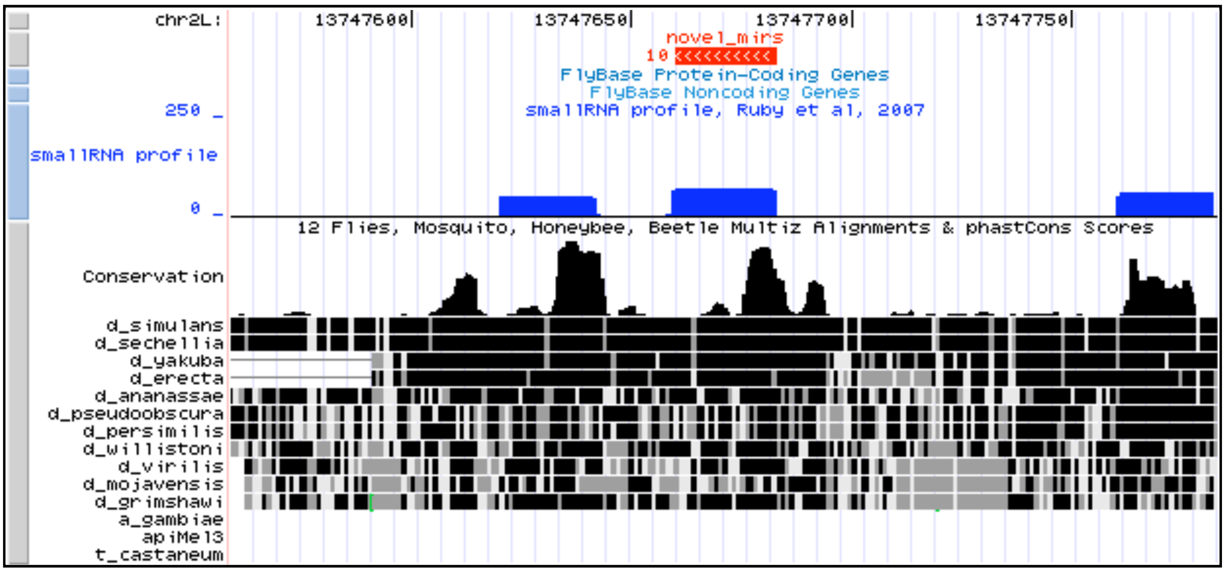

genomic context

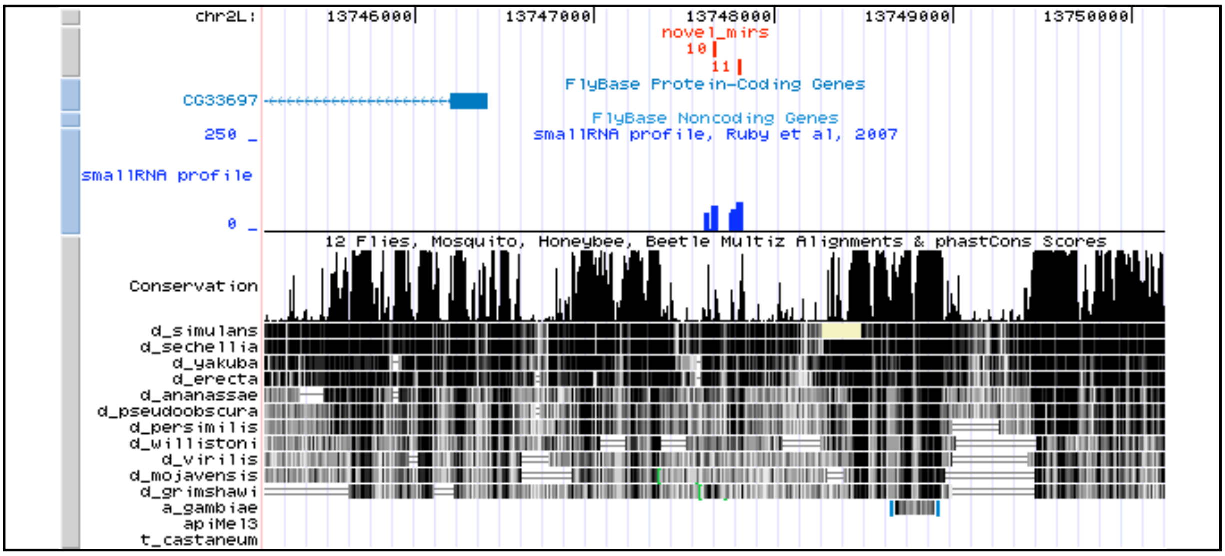

rnafold / alifold prediction

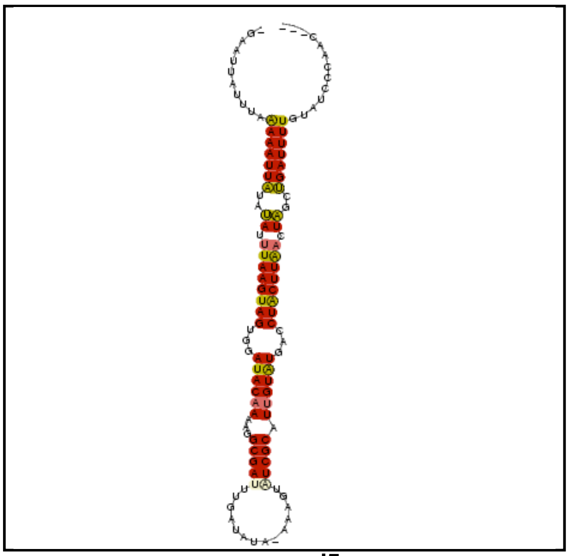

manual5-

Hits in RNA libraries

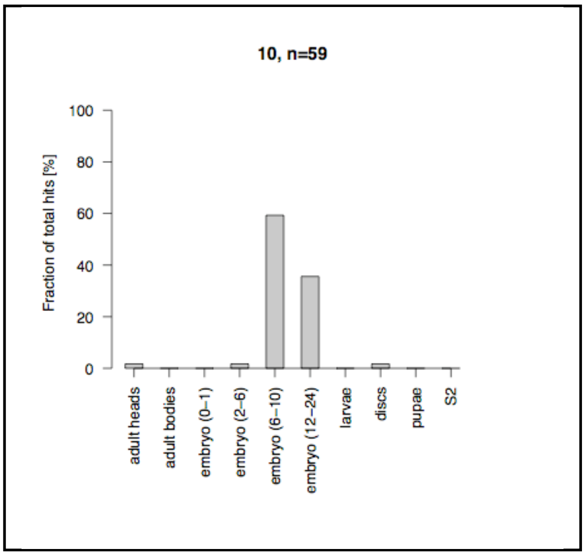

miRNA 11 chr2L : 13747801 - 13747824 Transcribed strand: - cluster4

predicted as: locus4

Validated: detected in small RNA sequencing data, validated by Northern blot

Mature sequence: UAAGUAGUAUCCAUUAAAGGGUUG

conservation profile

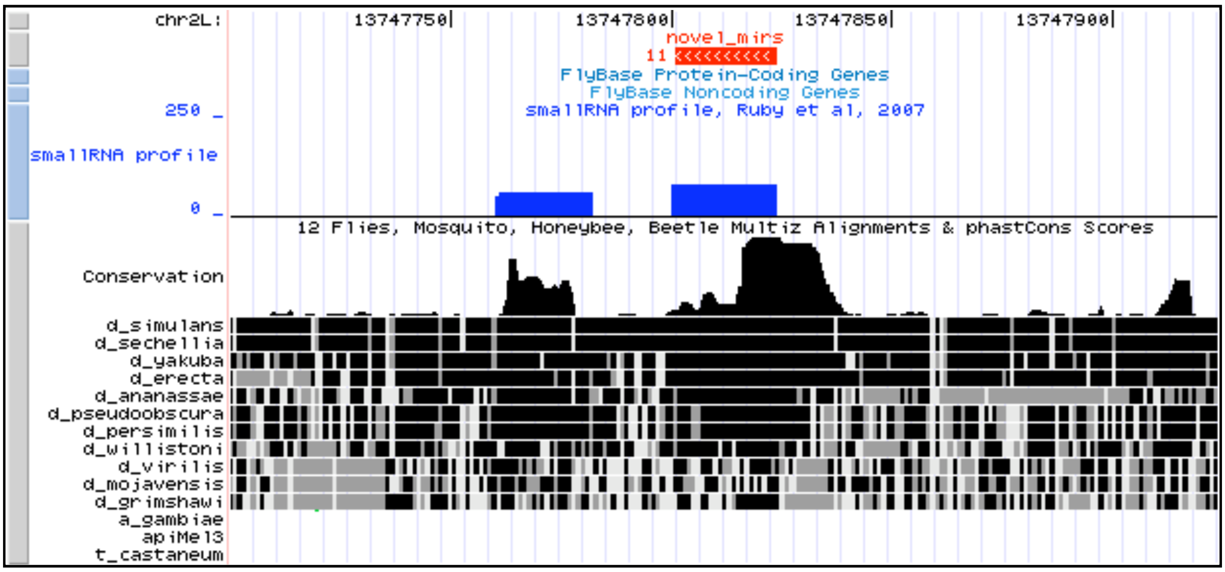

genomic context

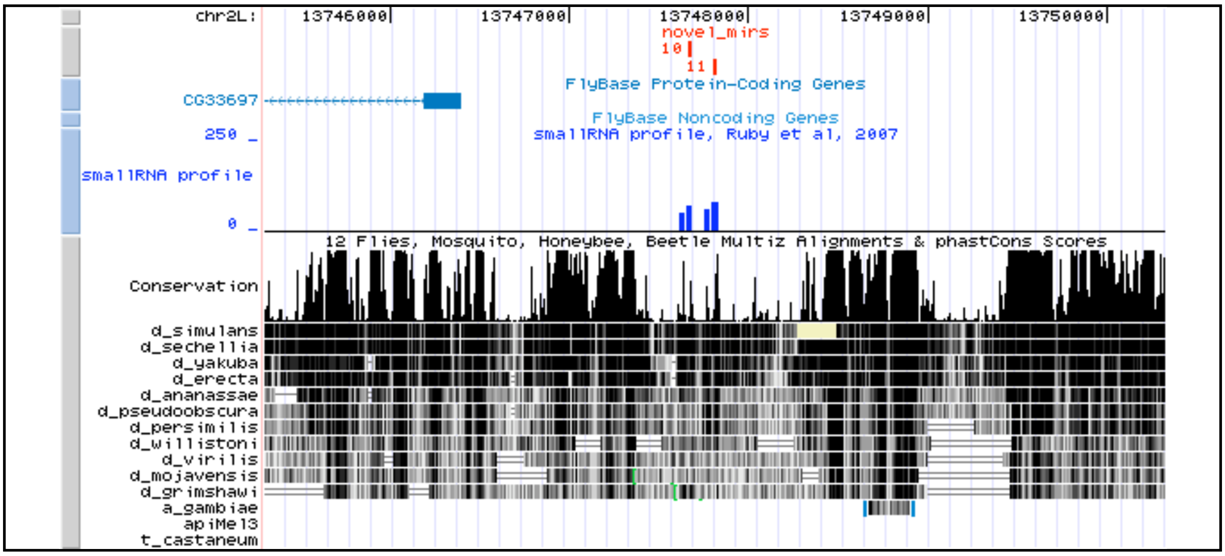

rnafold / alifold prediction

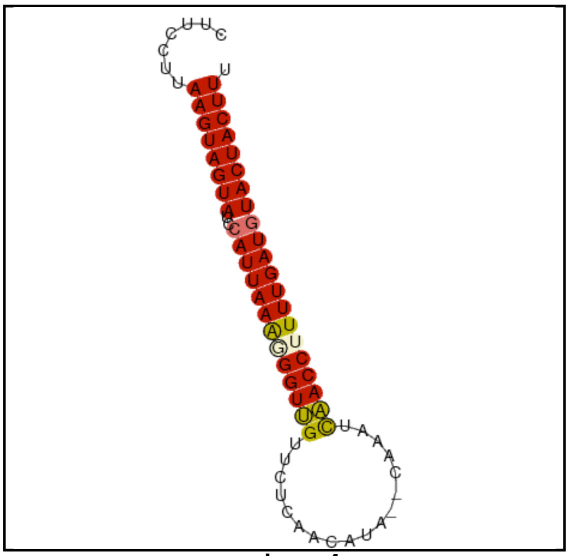

locus4-

Hits in RNA libraries

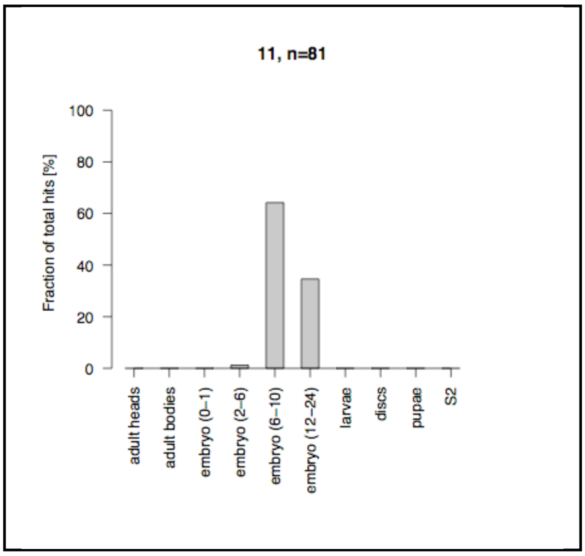

miRNA 12

chr2R : 1705689 - 1705773 Transcribed strand: +

predicted as: locus26

Validated: validated by Northern blot only

Mature sequence: n.d.,sequence matched by Northern probe: GGCUGCUGUUUGUGUUUUGAU

conservation profile

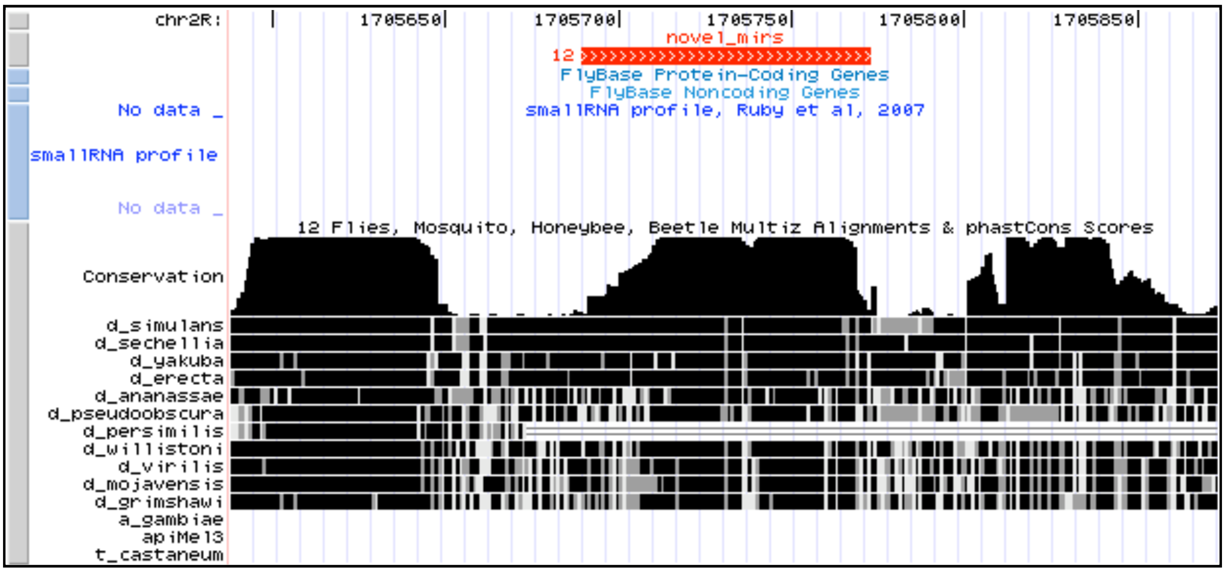

genomic context

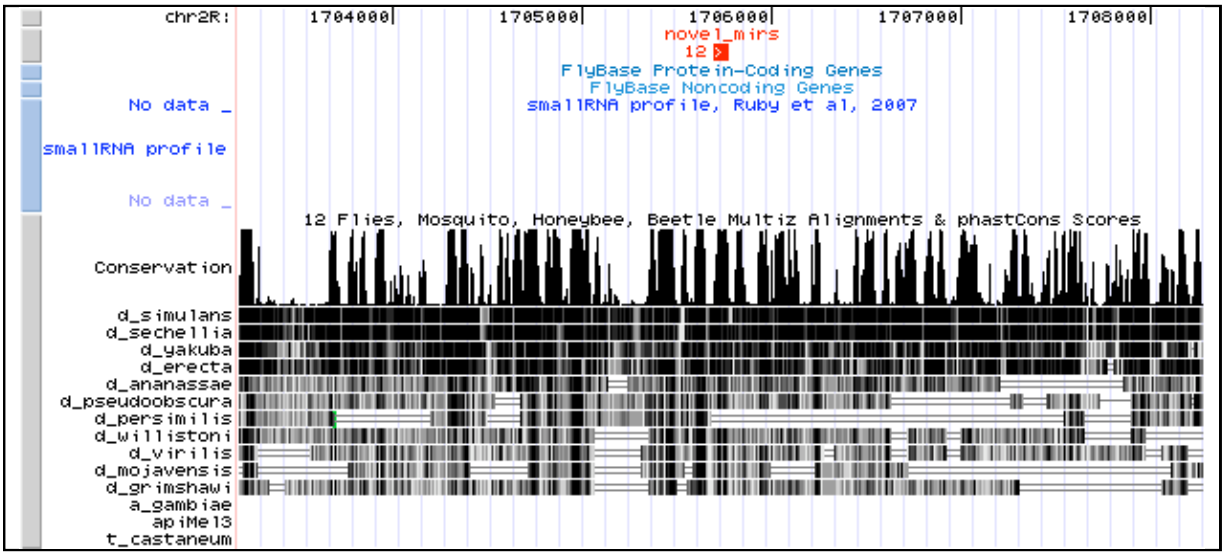

rnafold / alifold prediction

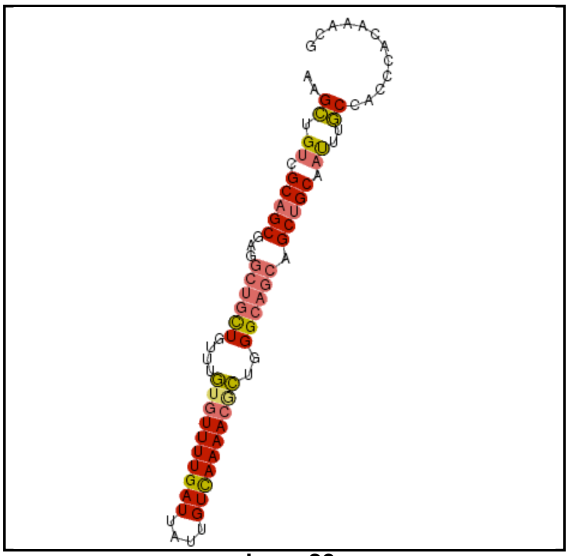

locus26+

Hits in RNA libraries

miRNA 13

chr2R : 4332505 - 4332526 Transcribed strand: +

predicted as:

Validated: detected in small RNA sequencing data

Mature sequence: UCUCGAAUAGCGUUGUGACUGA

conservation profile

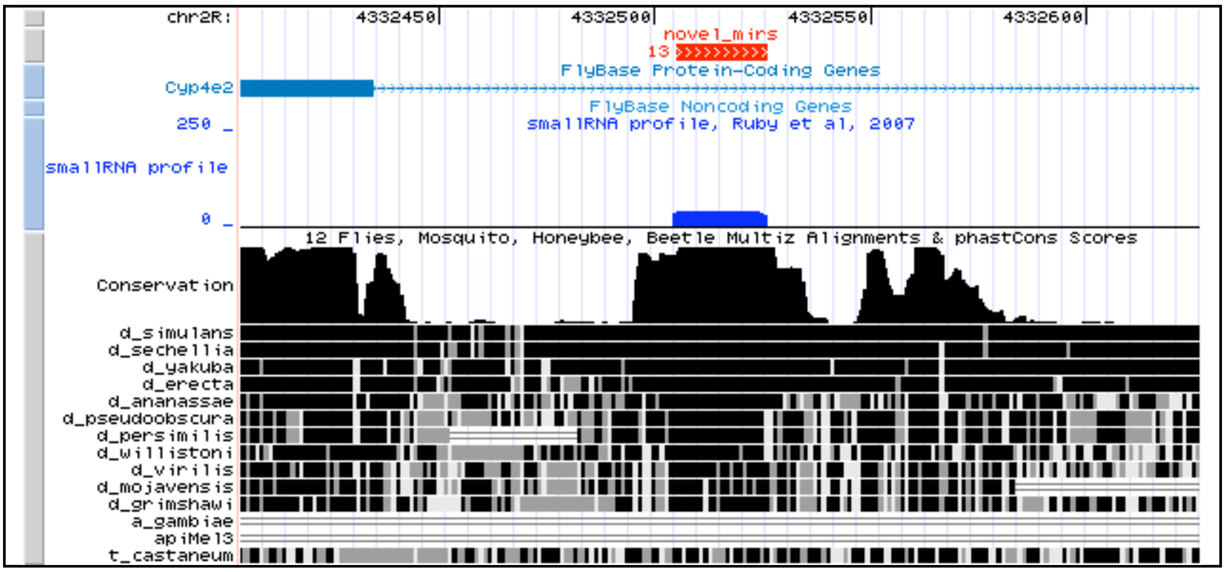

genomic context

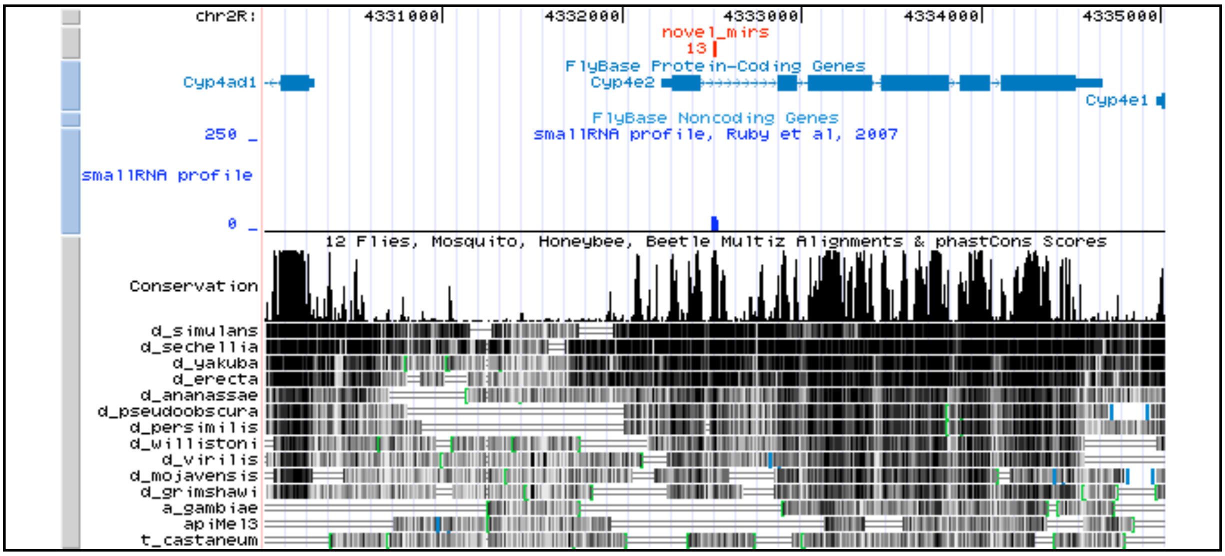

rnafold / alifold prediction

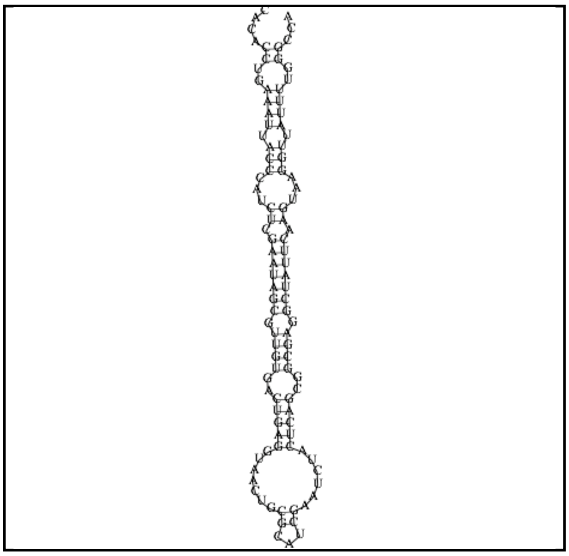

Hits in RNA libraries

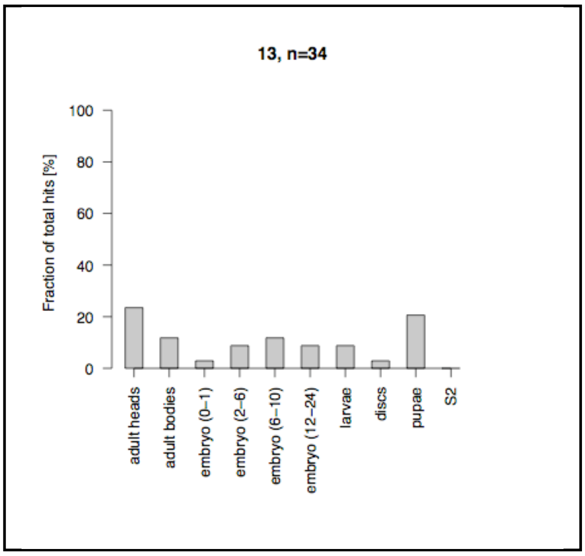

miRNA 14

chr2R : 5200583 - 5200607 Transcribed strand: -

predicted as:

Validated: detected in small RNA sequencing data

Mature sequence: UAAAGUAAAUAGUCUGGAUUGAUG

conservation profile

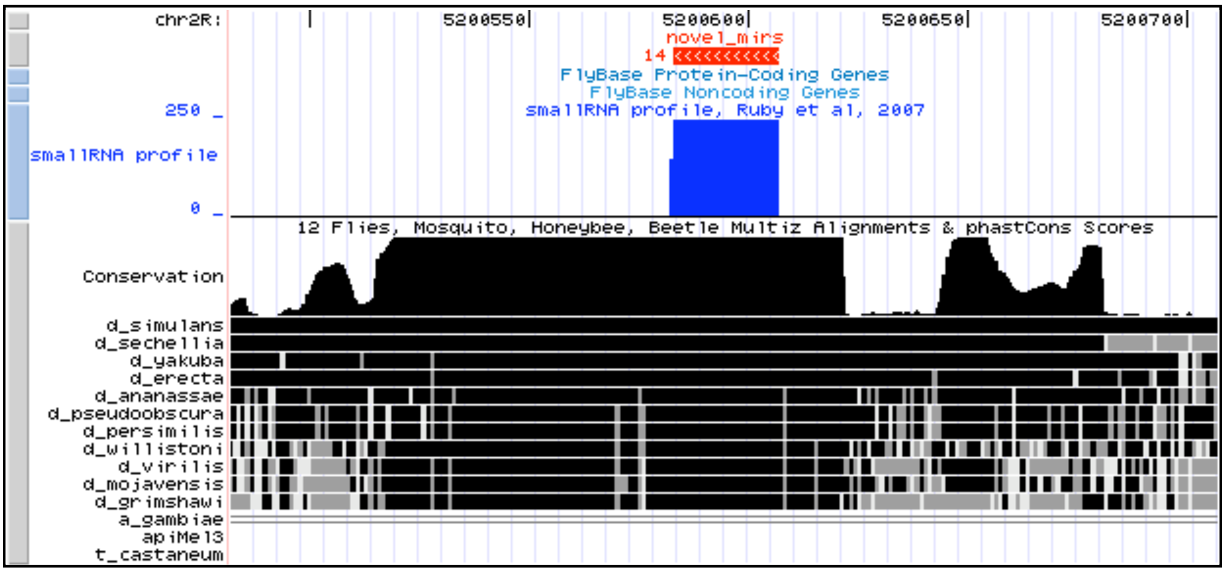

genomic context

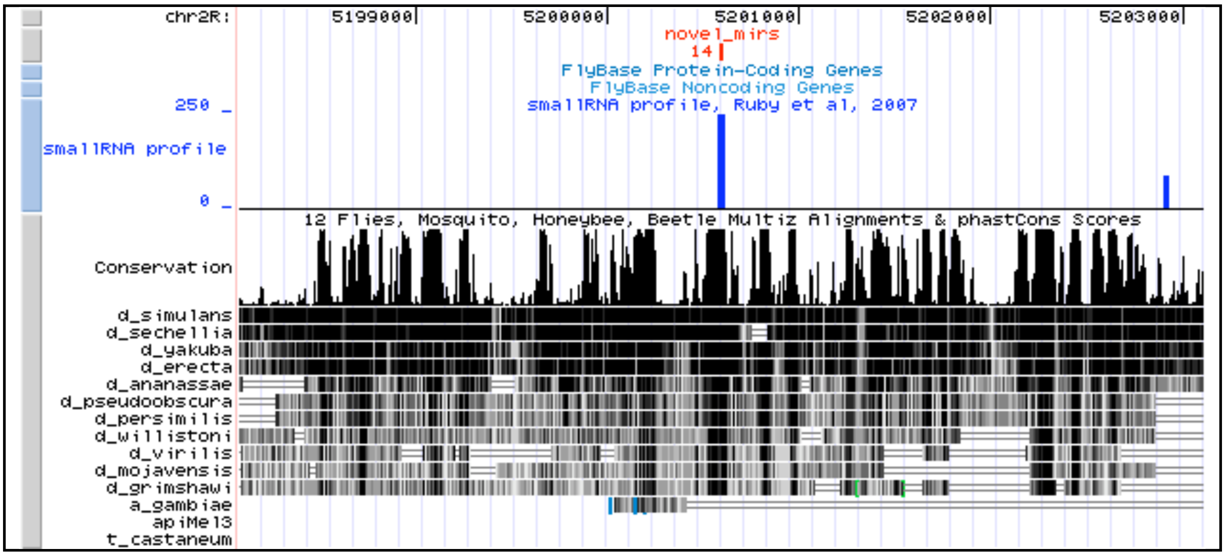

rnafold / alifold prediction

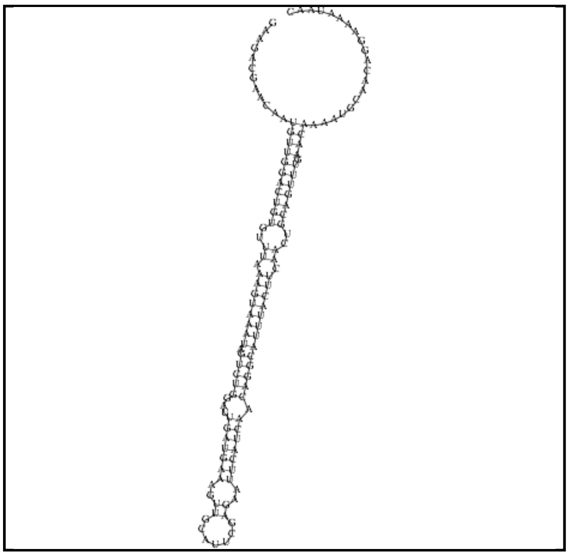

Hits in RNA libraries

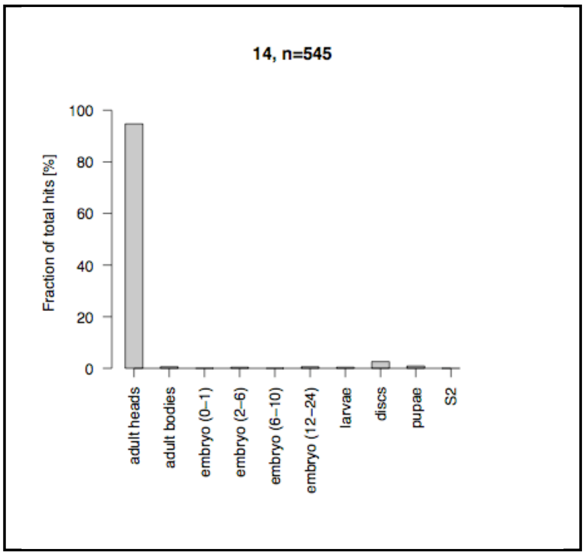

miRNA 15

chr2R : 8046393 - 8046414 Transcribed strand: -

predicted as:

Validated: detected in small RNA sequencing data

Mature sequence: CCCCUUGUUGCAAACCUCACGC

conservation profile

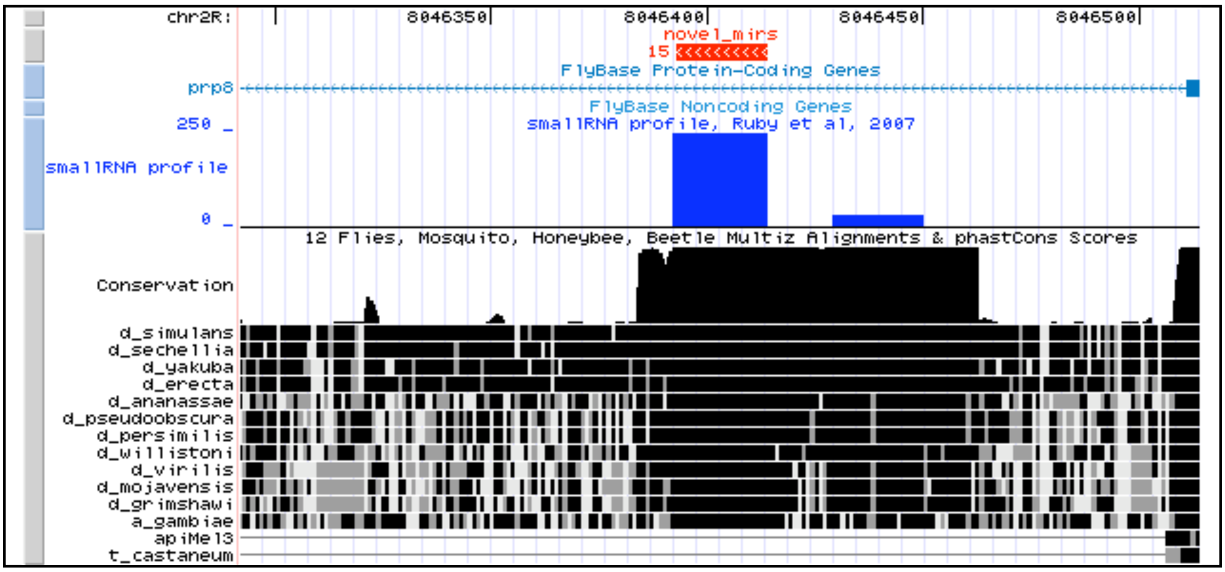

genomic context

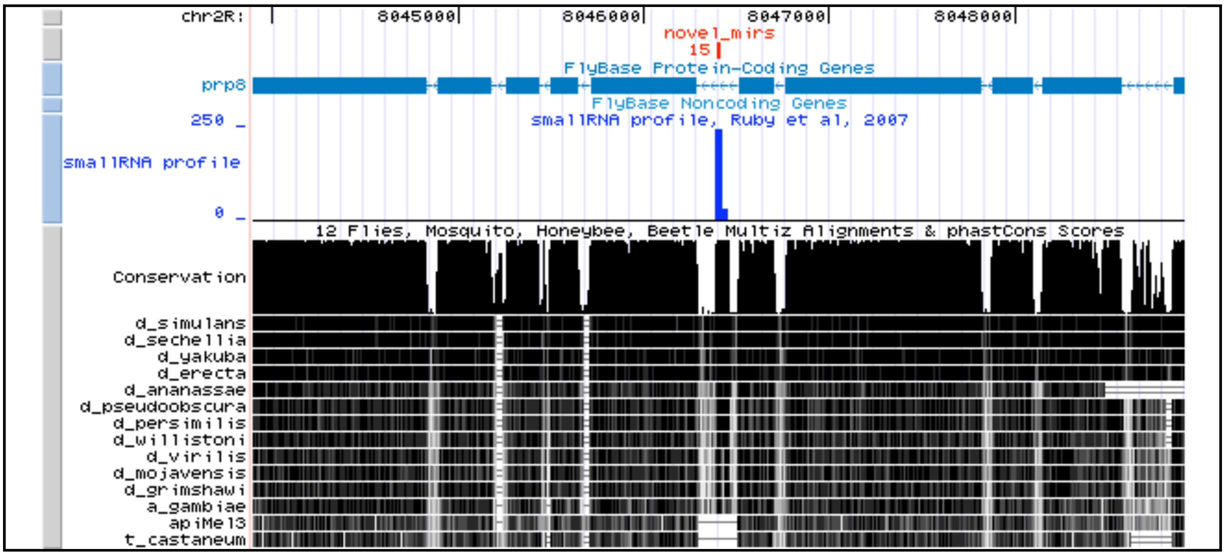

rnafold / alifold prediction

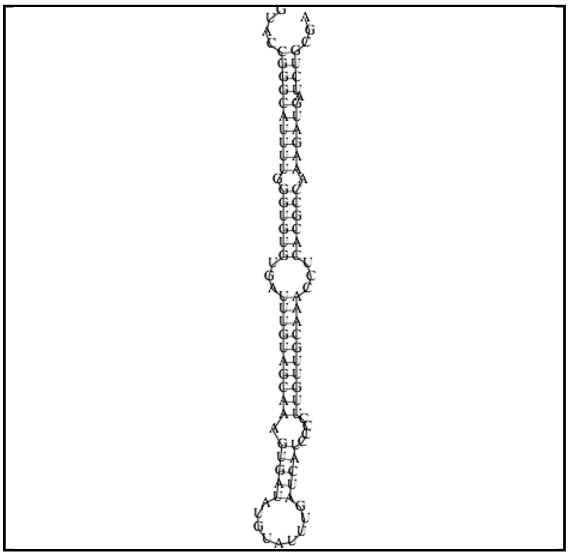

Hits in RNA libraries

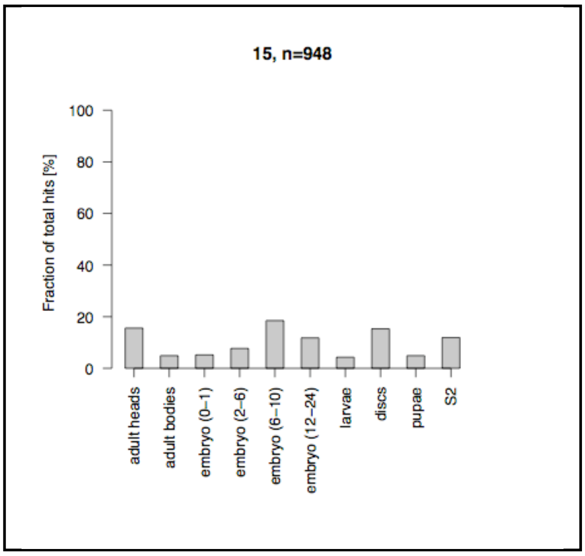

miRNA 16

chr2R : 10032871 - 10032891 Transcribed strand: -

predicted as:

Validated: detected in small RNA sequencing data

Mature sequence: UGUGAUGUGACGUAGUGGAAC

conservation profile

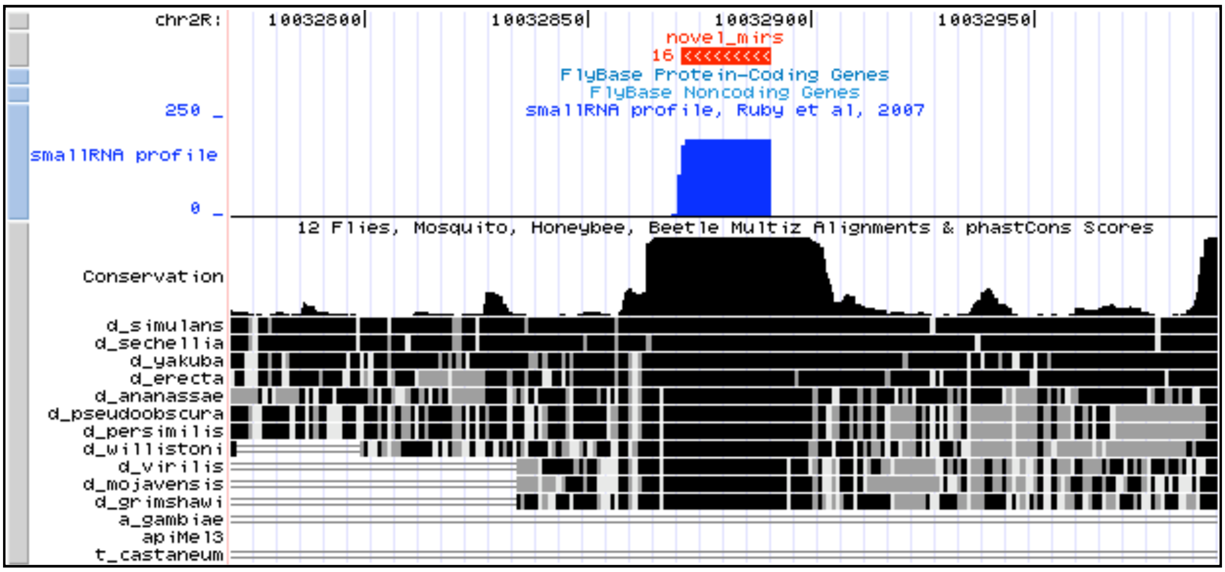

genomic context

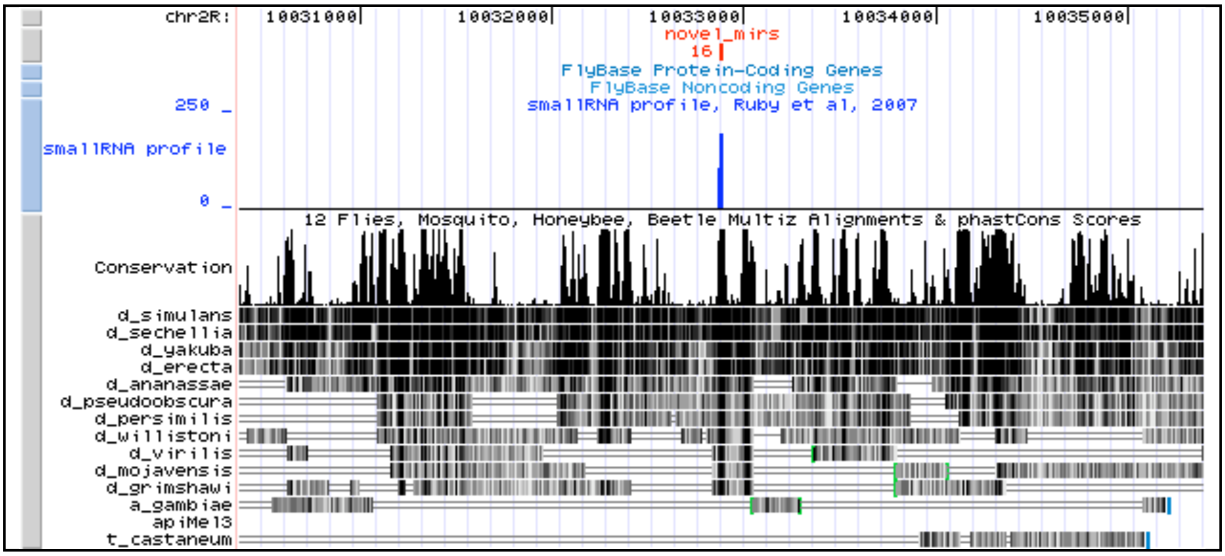

rnafold / alifold prediction

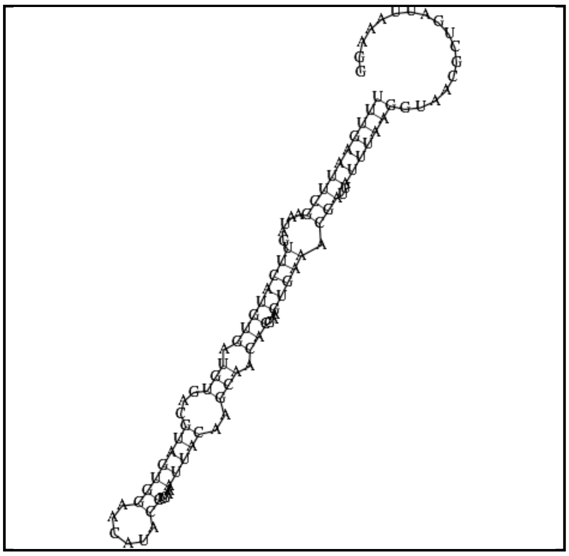

Hits in RNA libraries

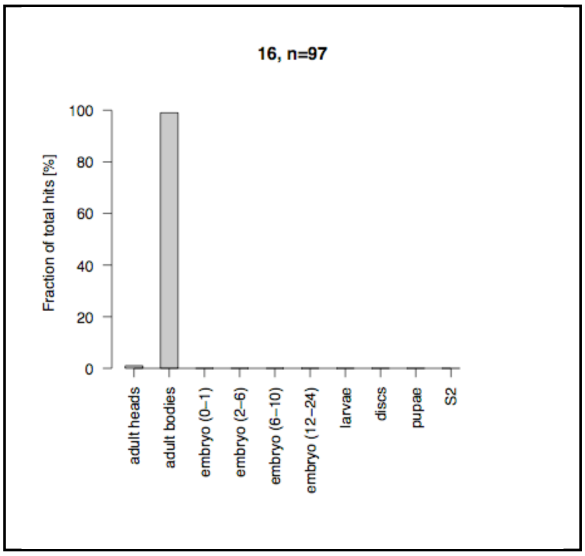

miRNA 17

chr2R : 16472719 - 16472740 **Transcribed strand: -**

predicted as:

Validated: detected in small RNA sequencing data

Mature sequence: UUAAAGUUGUAGUUUGGAAAGU

conservation profile

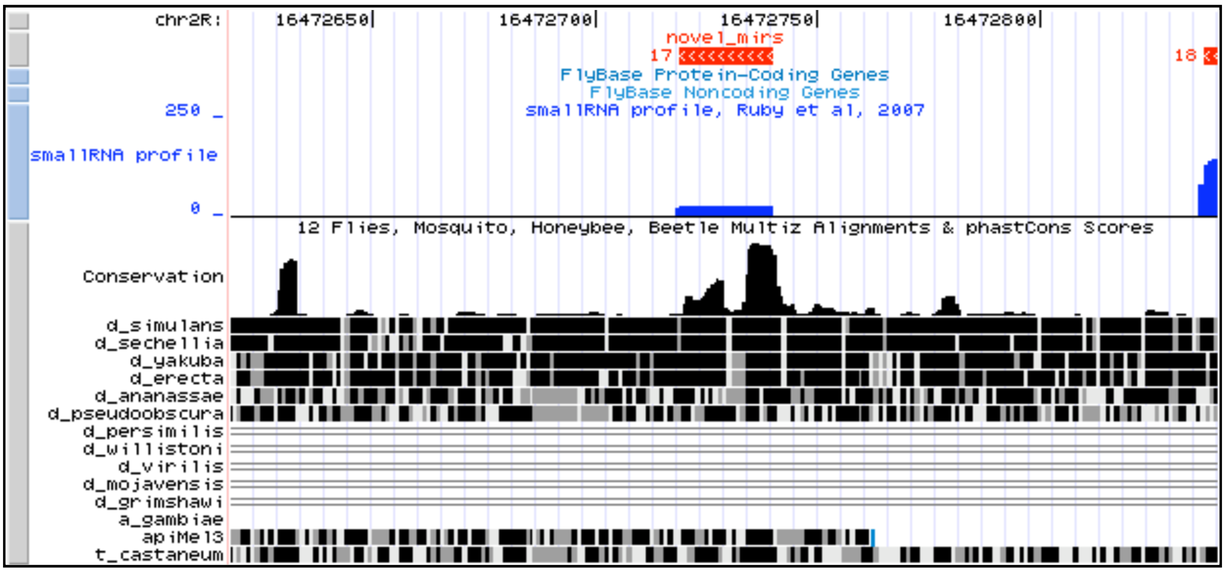

genomic context

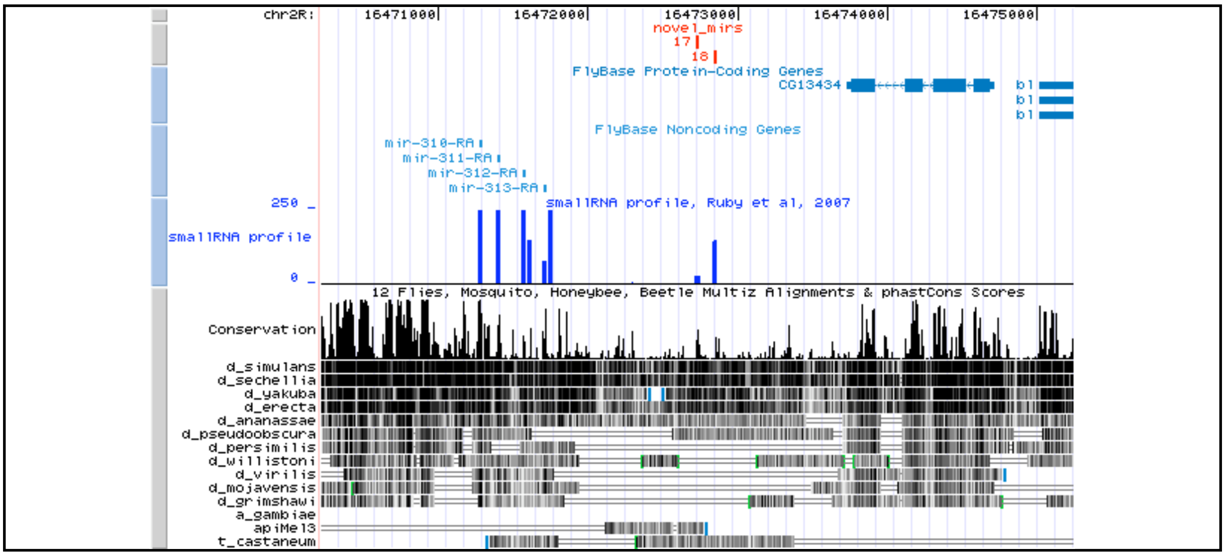

rnafold / alifold prediction

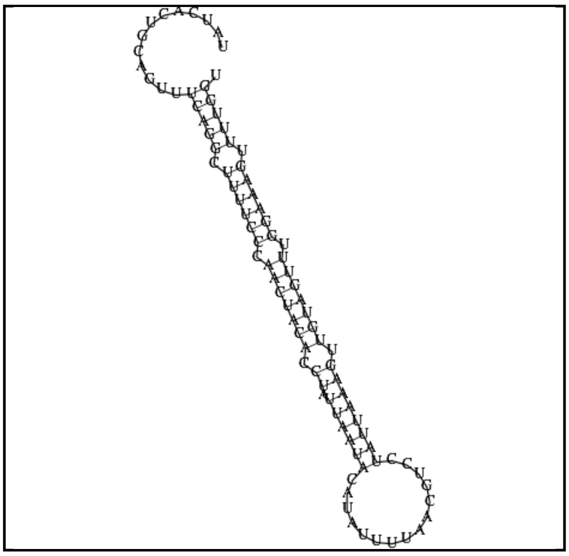

Hits in RNA libraries

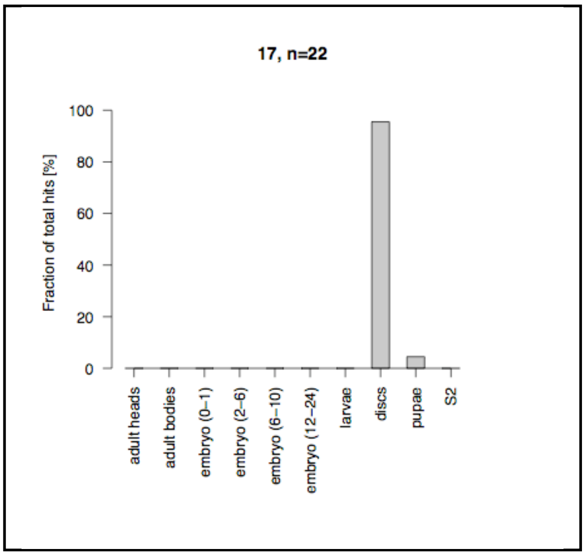

miRNA 18

chr2R : 16472837 - 16472858   Transcribed strand: -

predicted as:

Validated: detected in small RNA sequencing data

Mature sequence: AGUACACGUUUCUGGUACUAAG

conservation profile

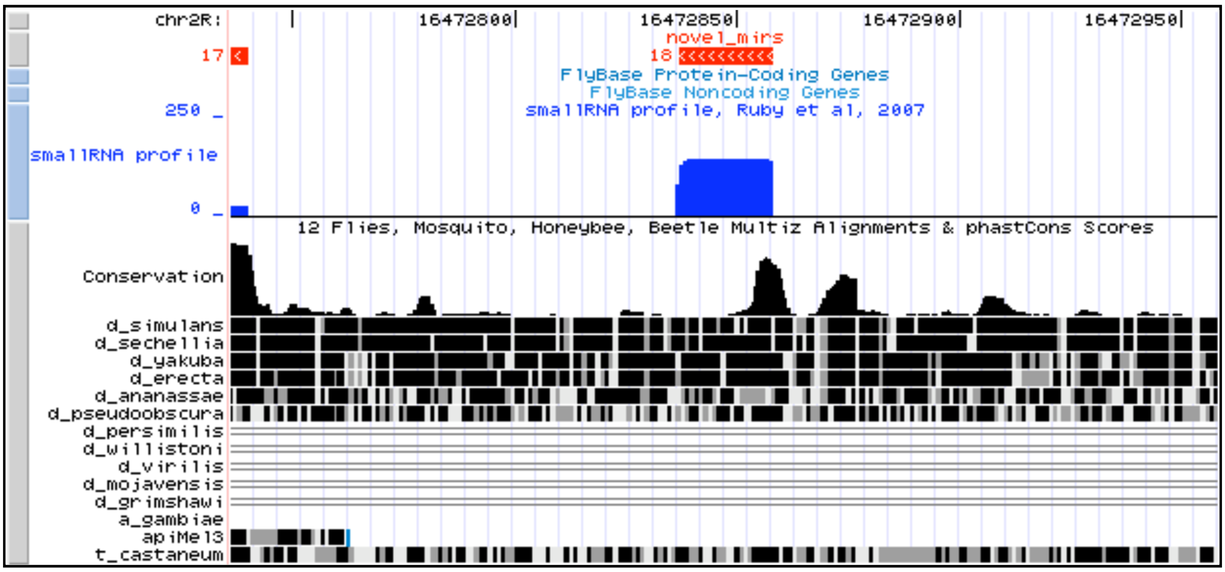

genomic context

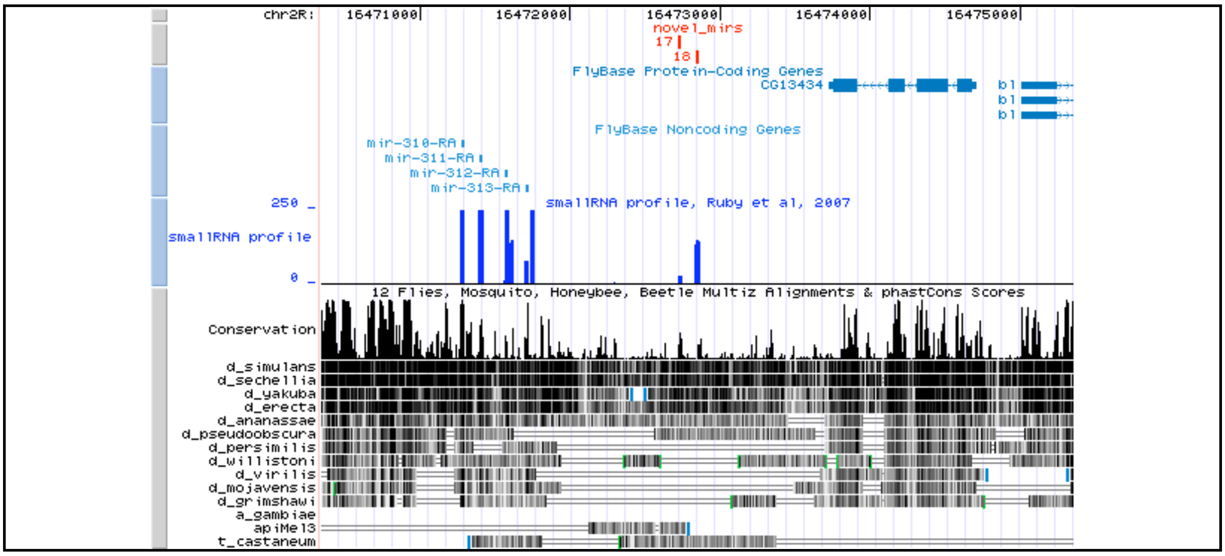

rnafold / alifold prediction

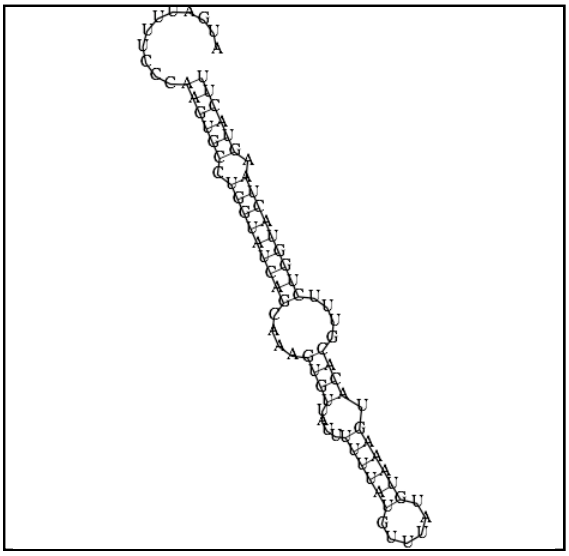

Hits in RNA libraries

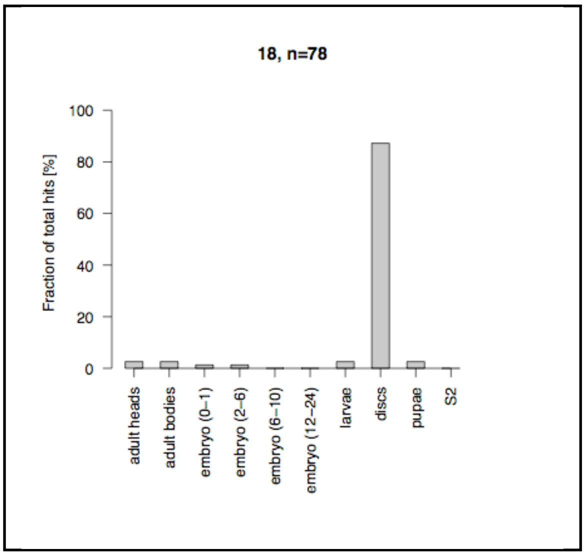

miRNA 19

chr3L : 8564922 - 8564946 Transcribed strand: +

predicted as:

Validated: detected in small RNA sequencing data

Mature sequence: AGAUAUGUUUGAUAUUCUUGGUUG

conservation profile

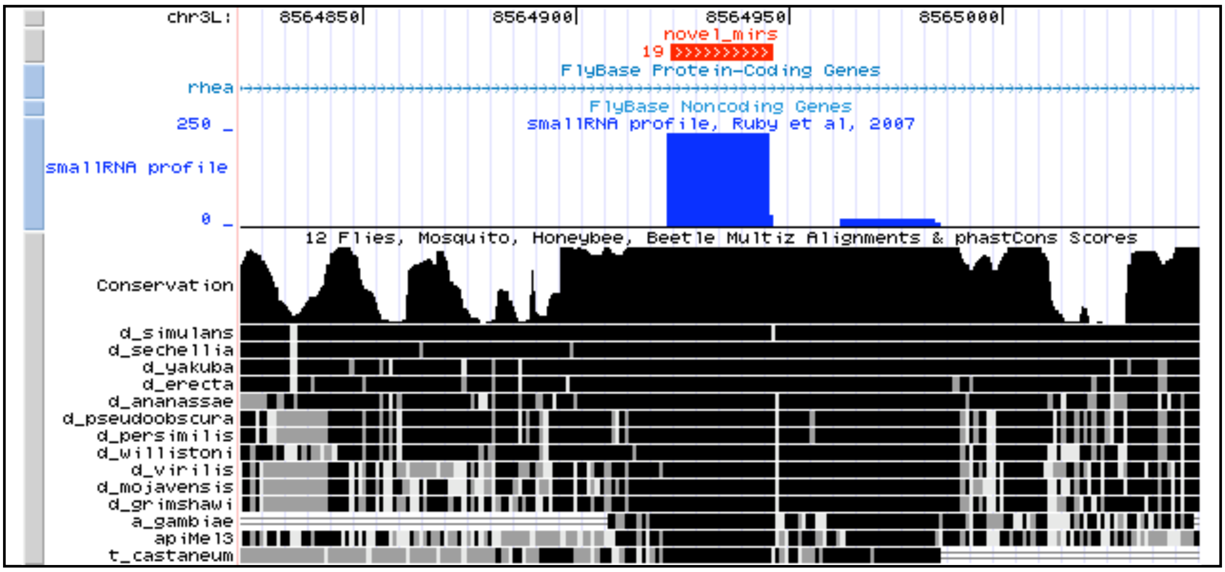

genomic context

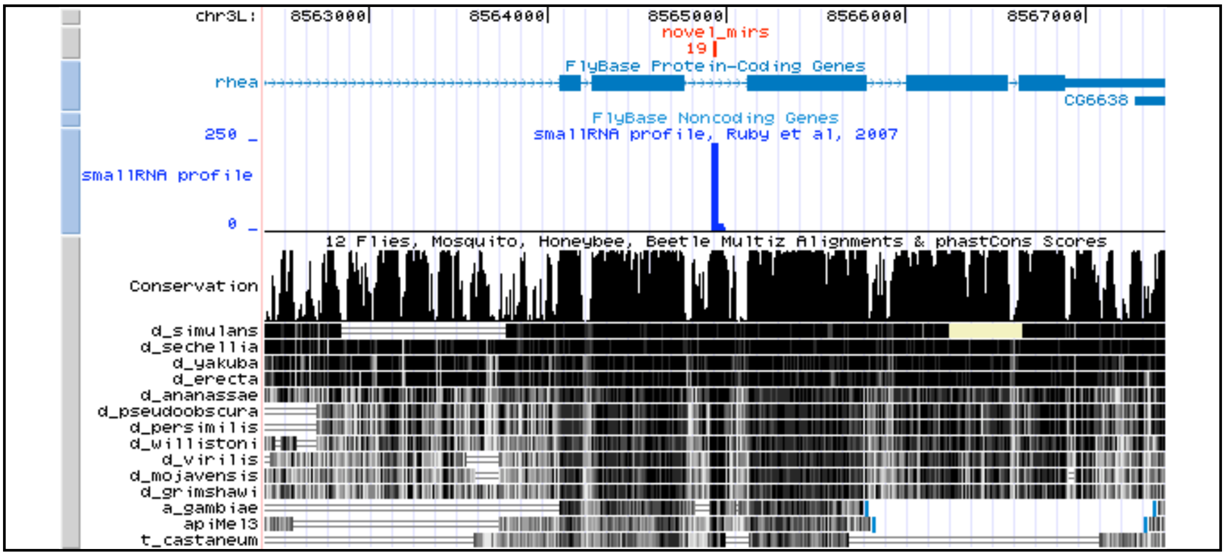

rnafold / alifold prediction

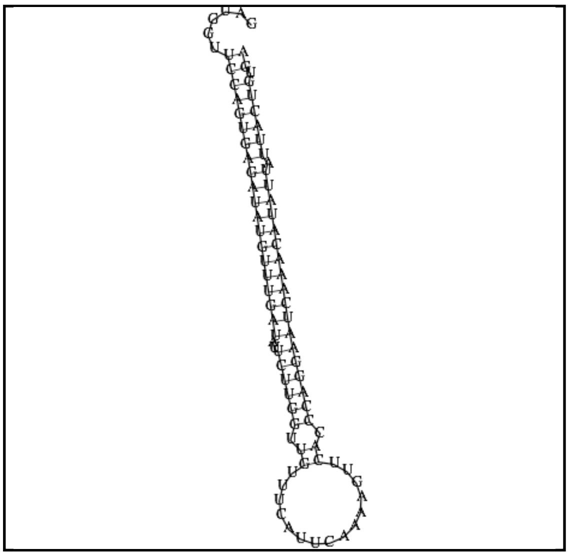

Hits in RNA libraries

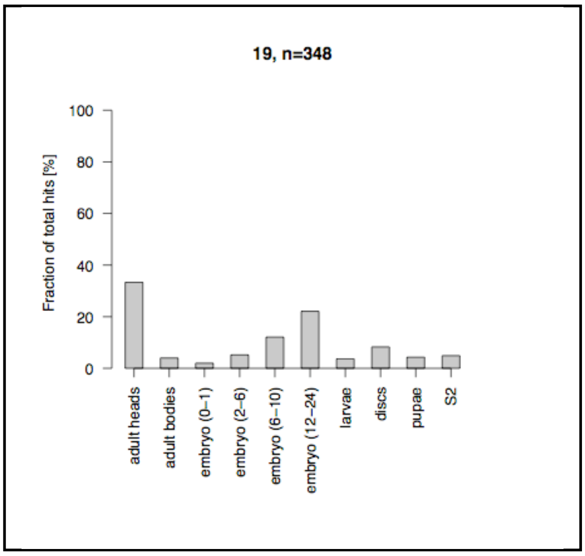

miRNA 20

chr3L : 11766840 - 11766862 Transcribed strand: -

predicted as:

Validated: detected in small RNA sequencing data

Mature sequence: UUUCGAGACCACUCUAAUCCAUU

conservation profile

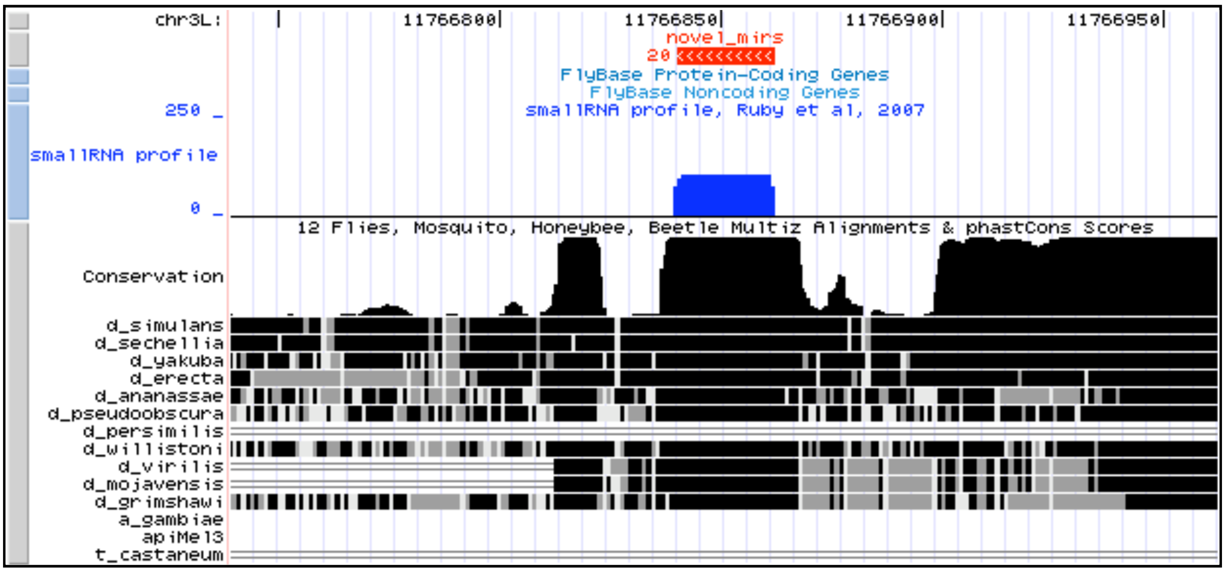

genomic context

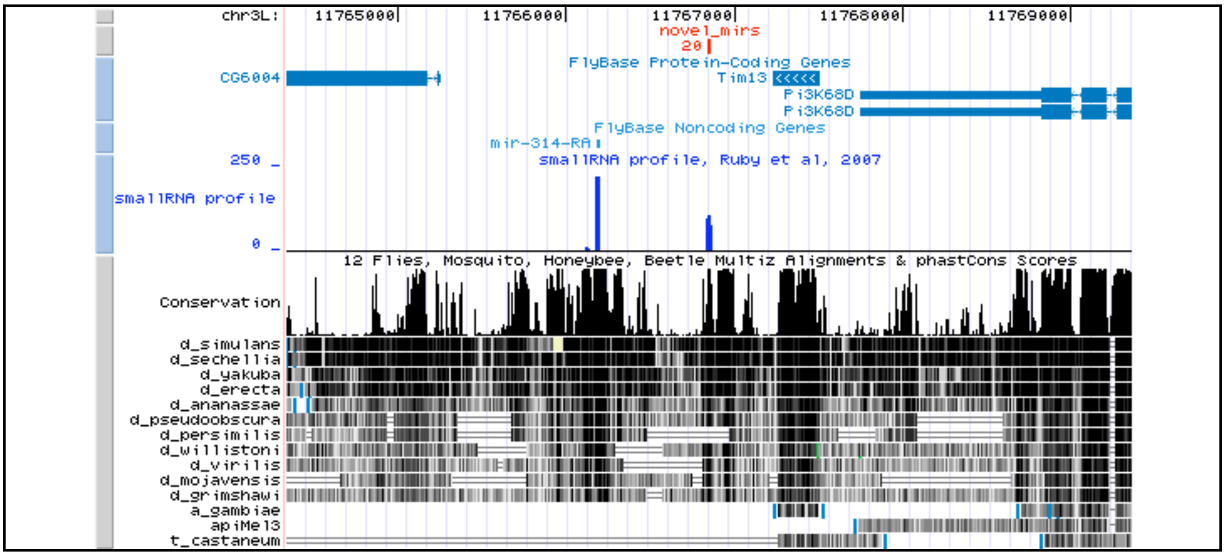

rnafold / alifold prediction

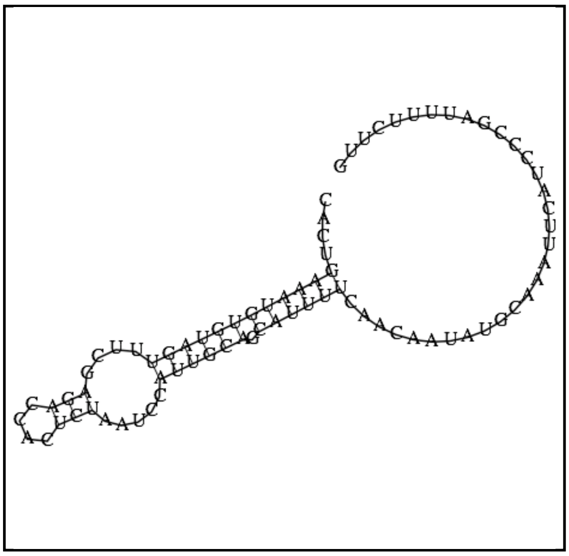

Hits in RNA libraries

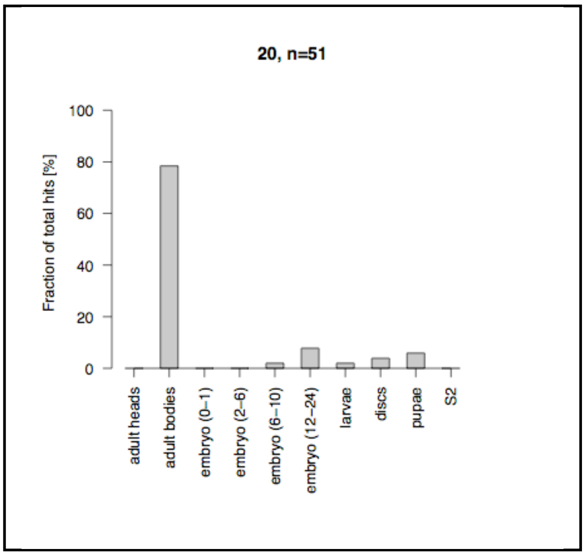

miRNA 21

chr3L : 19471681 - 19471701 Transcribed strand: -

predicted as:

Validated: detected in small RNA sequencing data

Mature sequence: GAGUUGGCCAAACGCGAAGGG

conservation profile

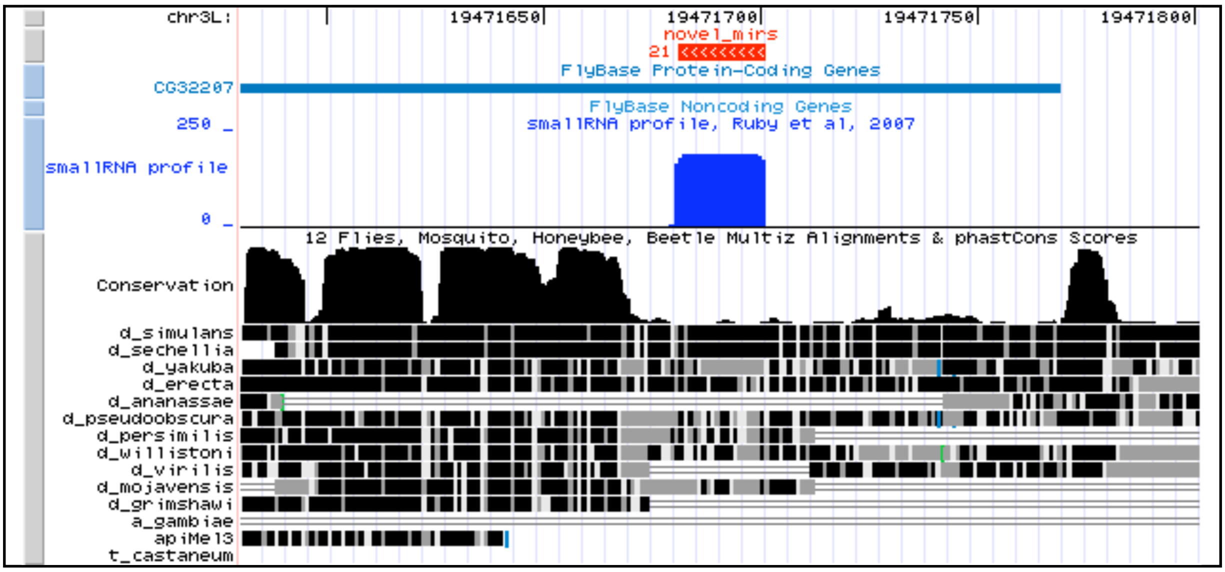

genomic context

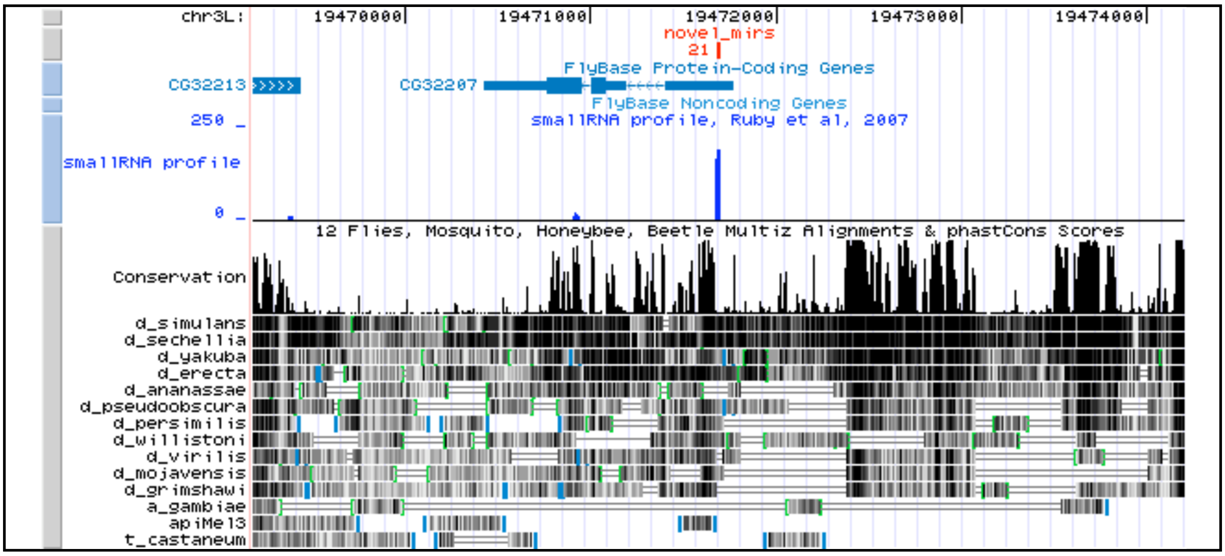

rnafold / alifold prediction

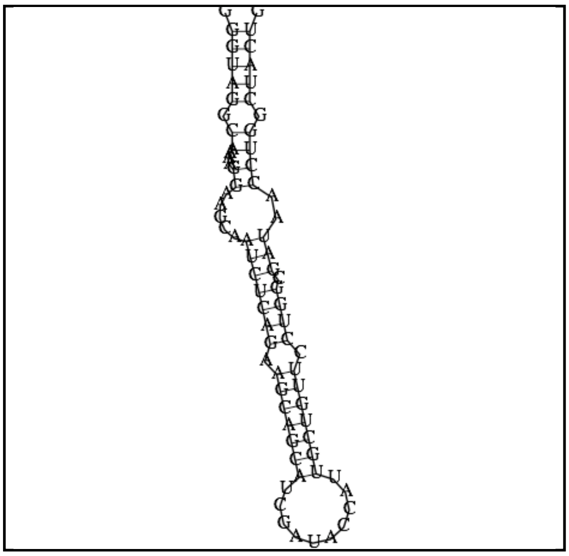

Hits in RNA libraries

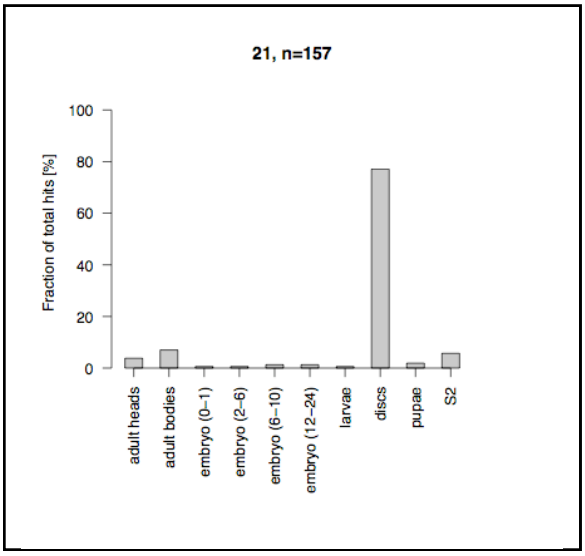

miRNA 22

chr3L : 21567186 - 21567209 Transcribed strand: +

predicted as:

Validated: detected in small RNA sequencing data

Mature sequence: UACUGGCCUACUAAGUCCCAAC

conservation profile

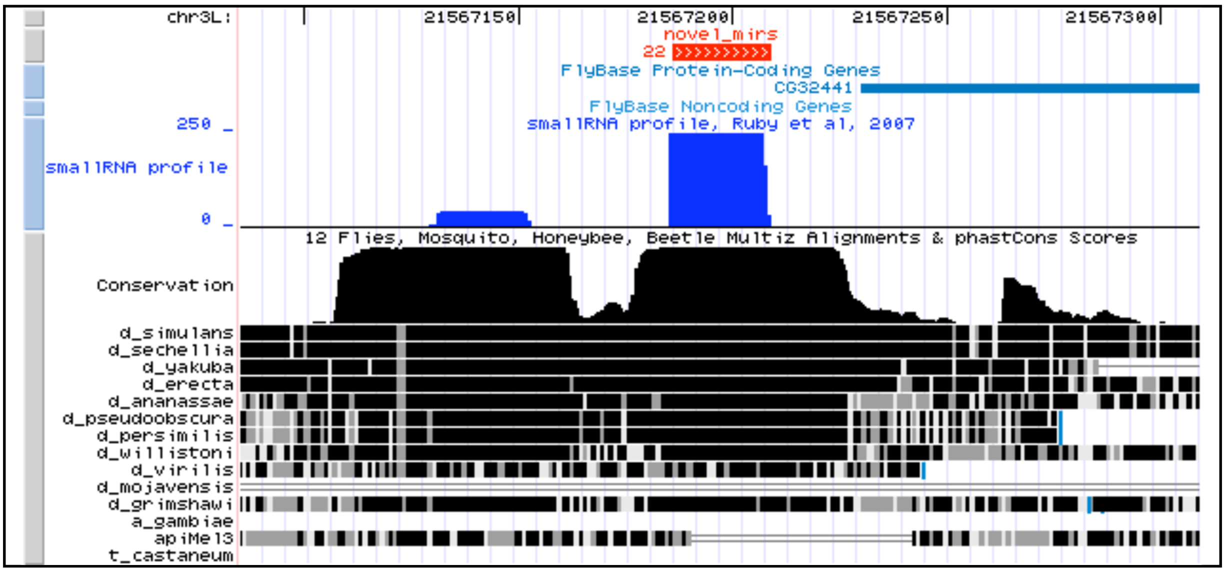

genomic context

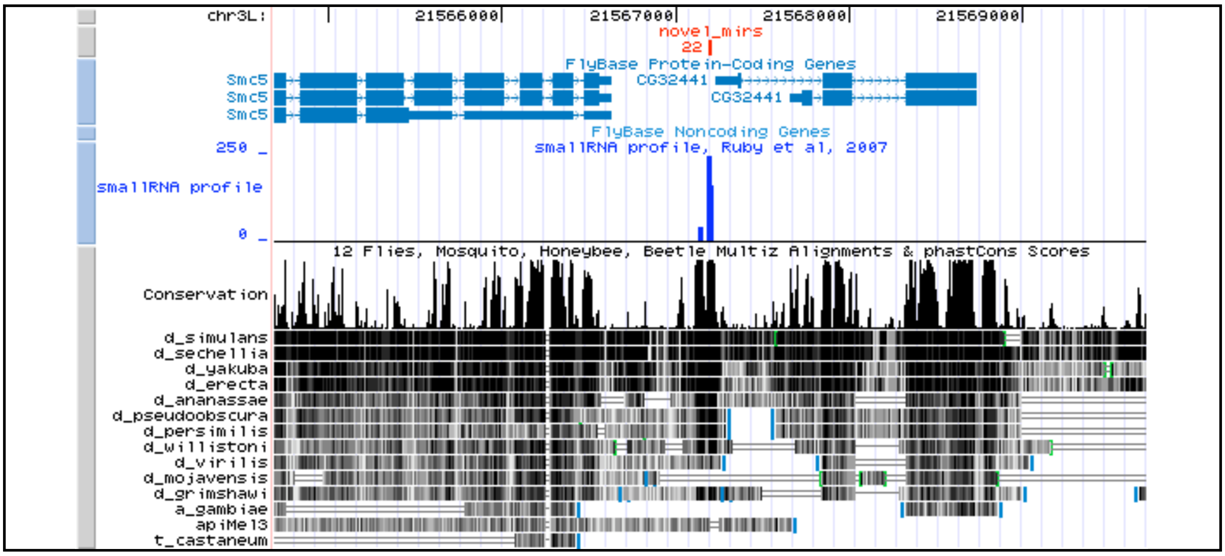

rnafold / alifold prediction

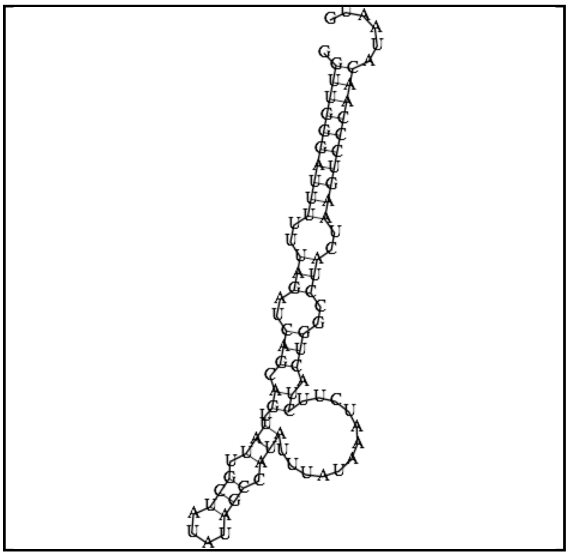

Hits in RNA libraries

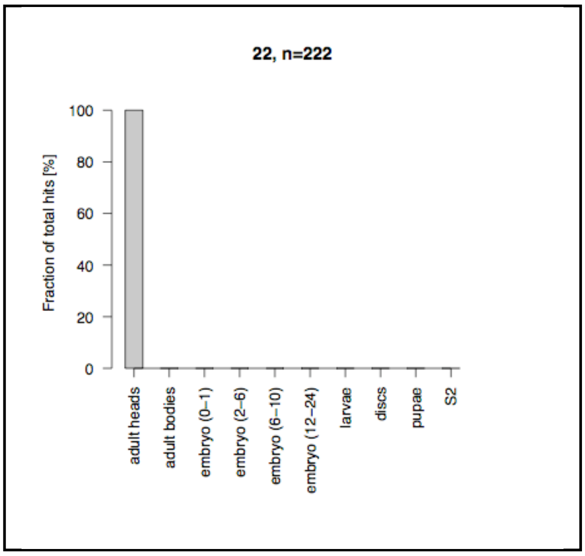

miRNA 23

chr3L : 22511310 - 22511331   Transcribed strand: -

predicted as:

Validated: detected in small RNA sequencing data

Mature sequence: UGAAACCGUCCAAAACUGAGGC

conservation profile

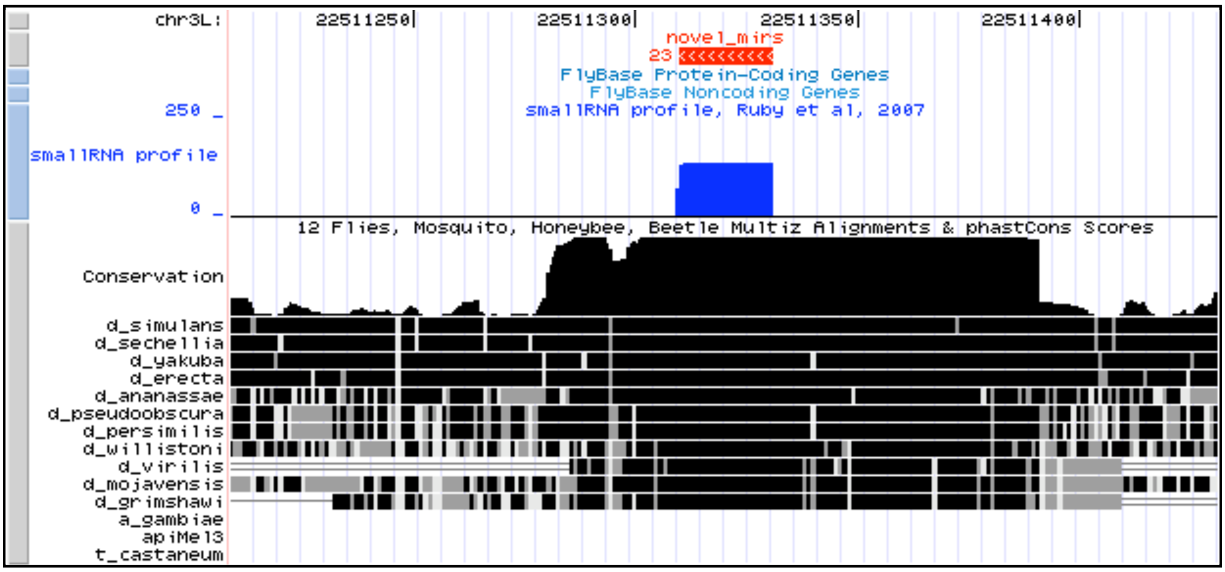

genomic context

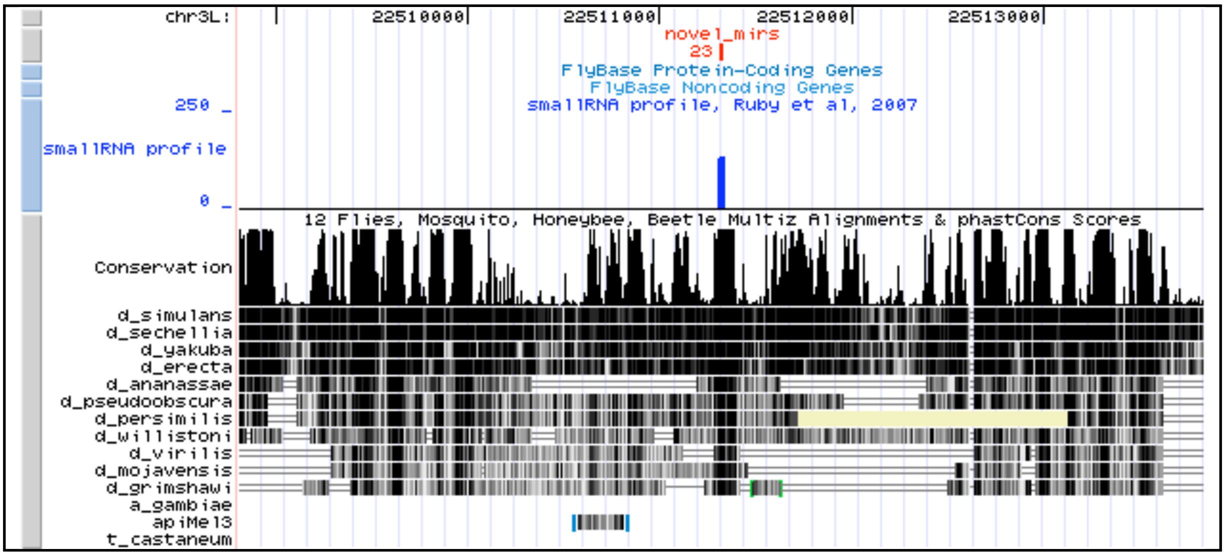

rnafold / alifold prediction

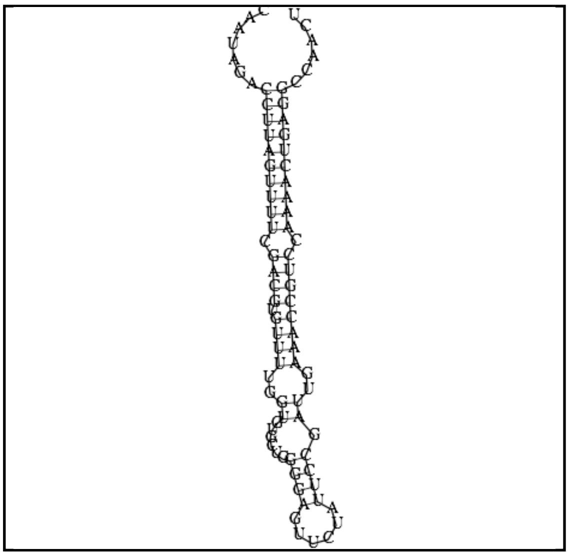

Hits in RNA libraries

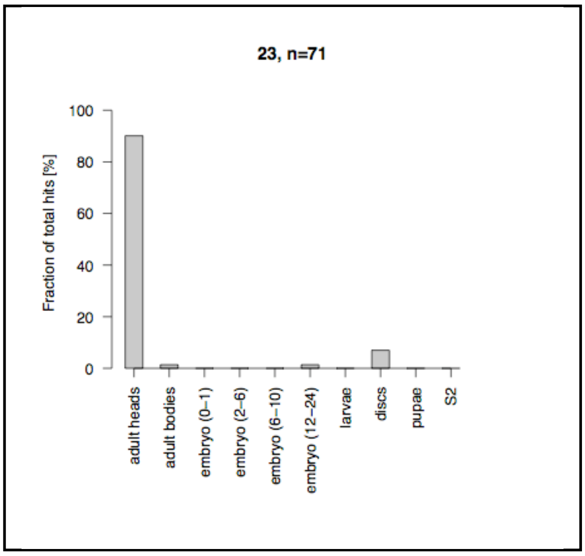

miRNA 24

chr3L : 22662402 - 22662423   Transcribed strand: -

predicted as:

Validated: detected in small RNA sequencing data

Mature sequence: UGAGAUUCUUCUAUUCUACUUU

conservation profile

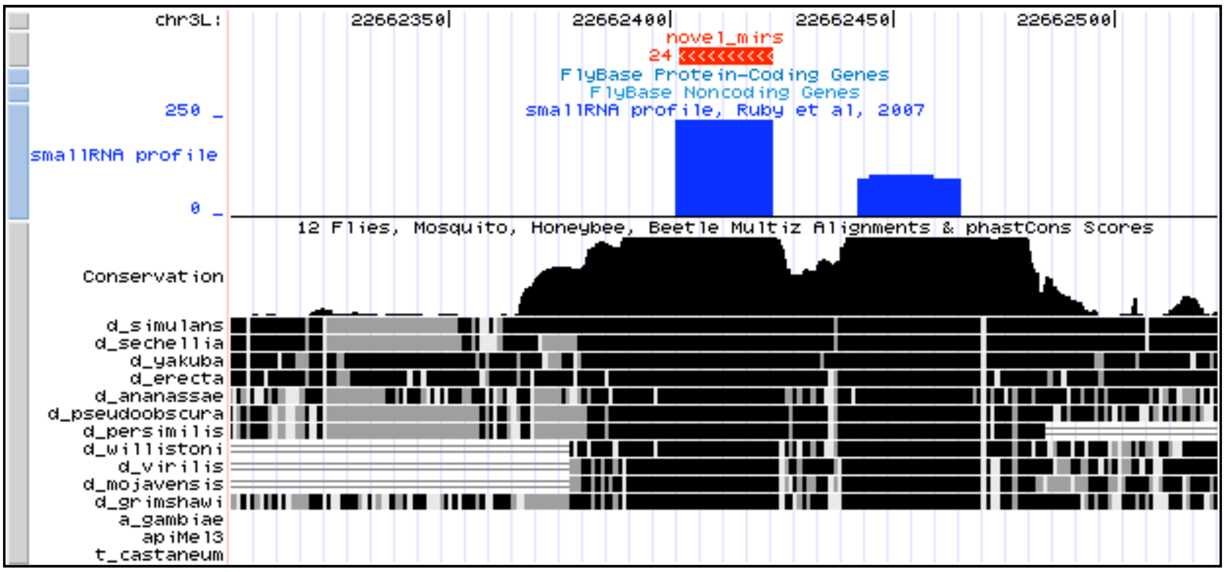

genomic context

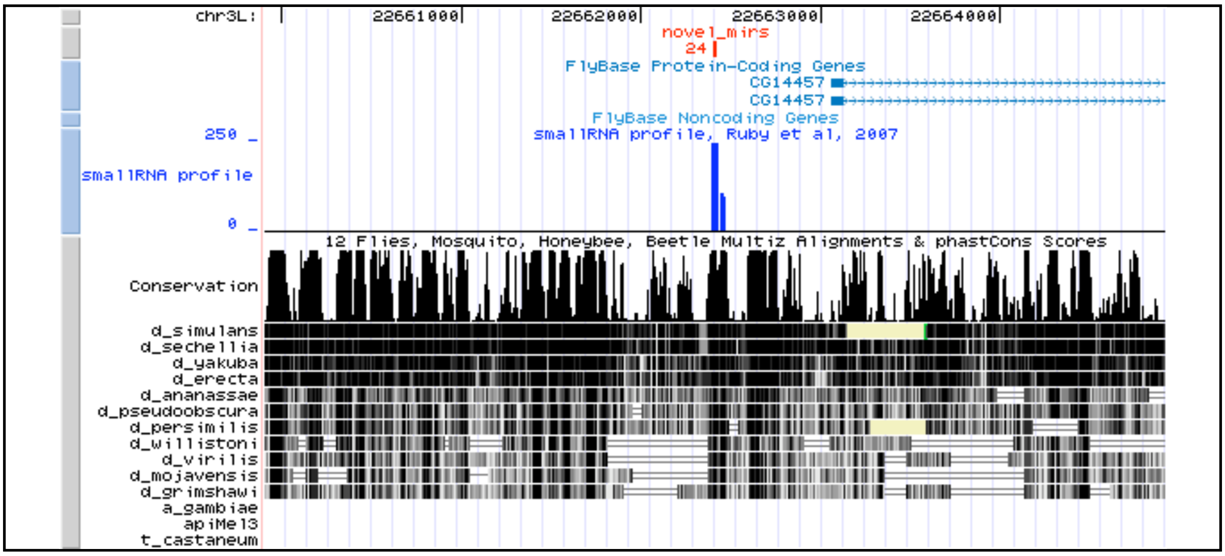

rnafold / alifold prediction

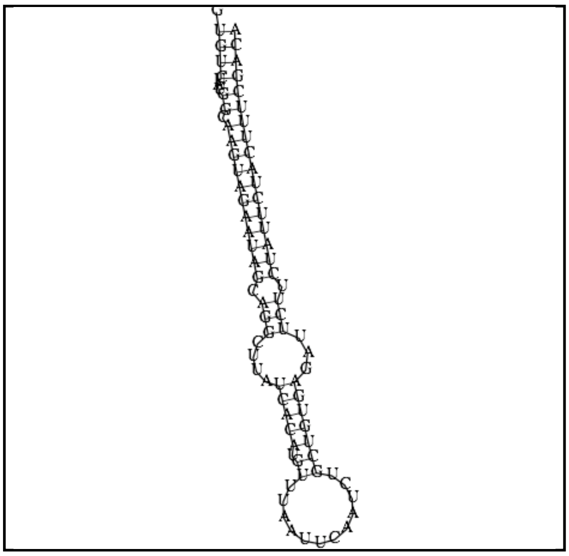

Hits in RNA libraries

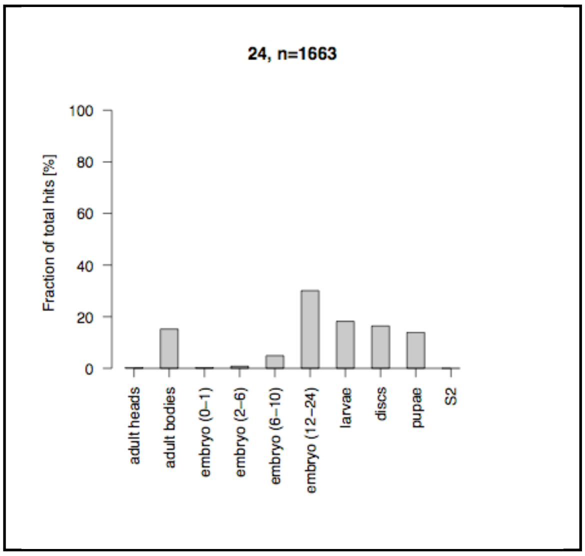

miRNA 25

chr3R : 121142 - 121163 Transcribed strand: +

predicted as: locus47

Validated: detected in small RNA sequencing data

Mature sequence: CUCCCUAACGGAGUCAGAUUG

conservation profile

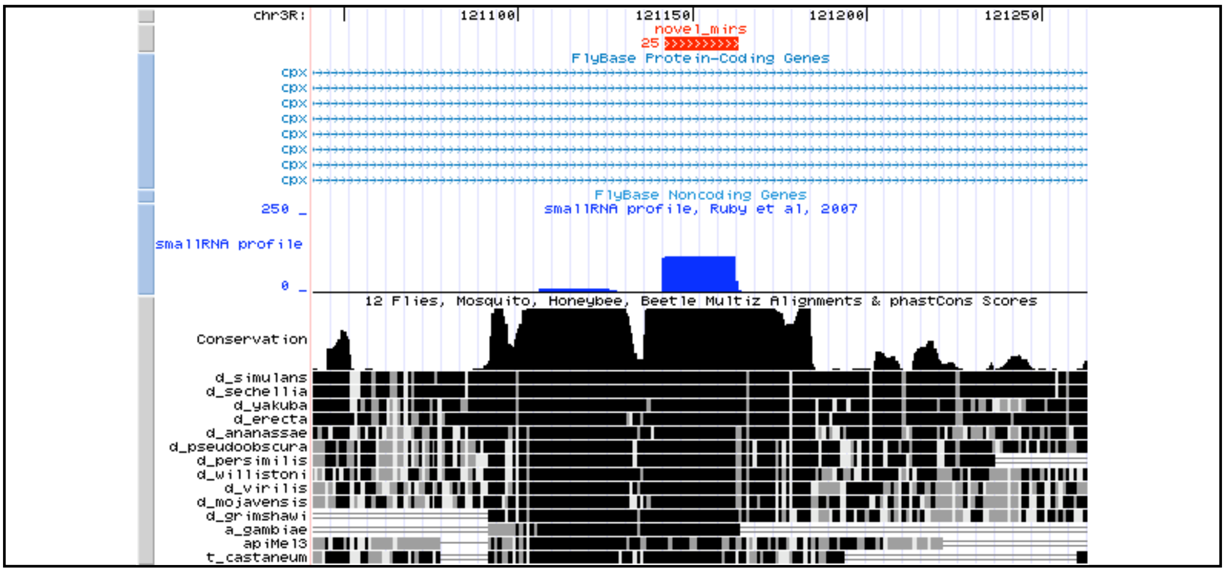

genomic context

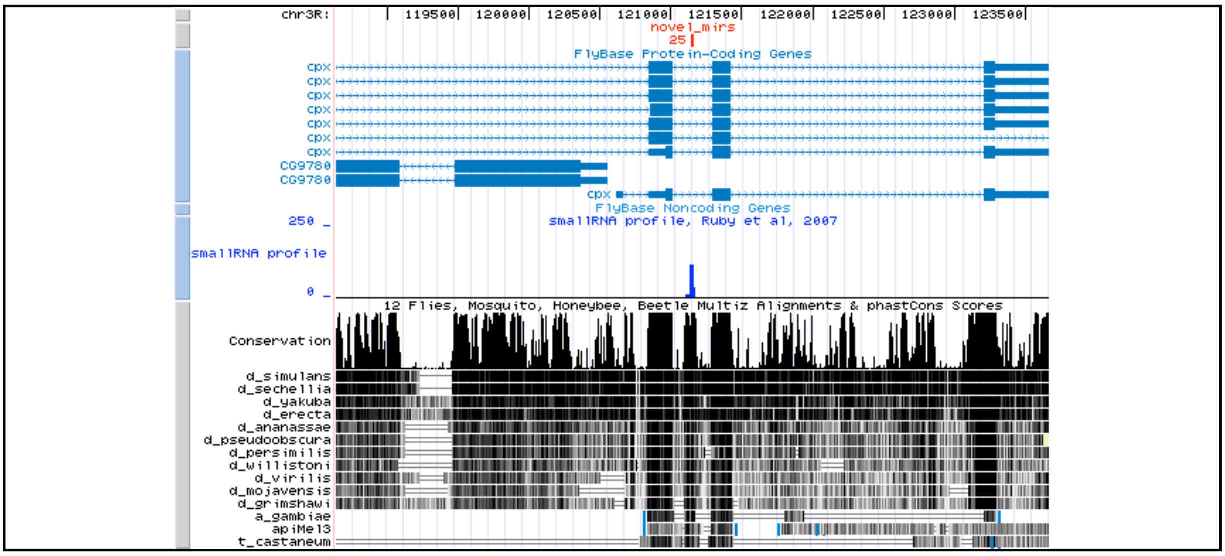

rnafold / alifold prediction

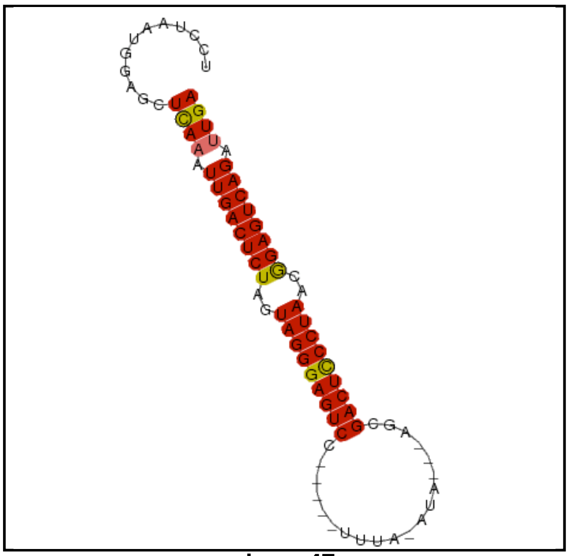

locus47+

Hits in RNA libraries

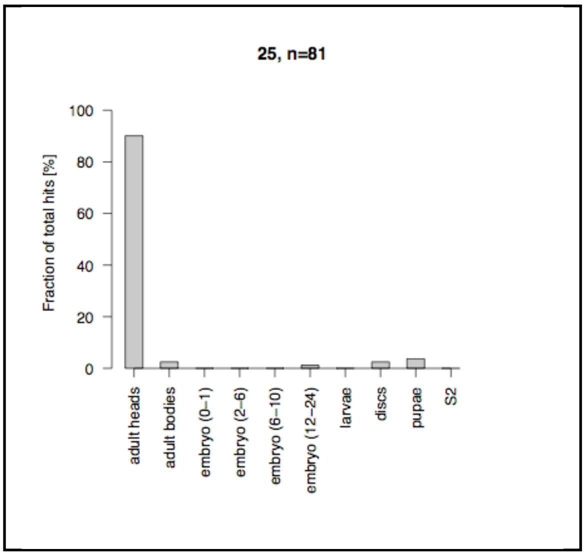

miRNA 26

chr3R : 2602139 - 2602161 Transcribed strand: +

predicted as: locus48

Validated: detected in small RNA sequencing data

Mature sequence: GAAGCUCGUCUCUACAGGUAUCU

conservation profile

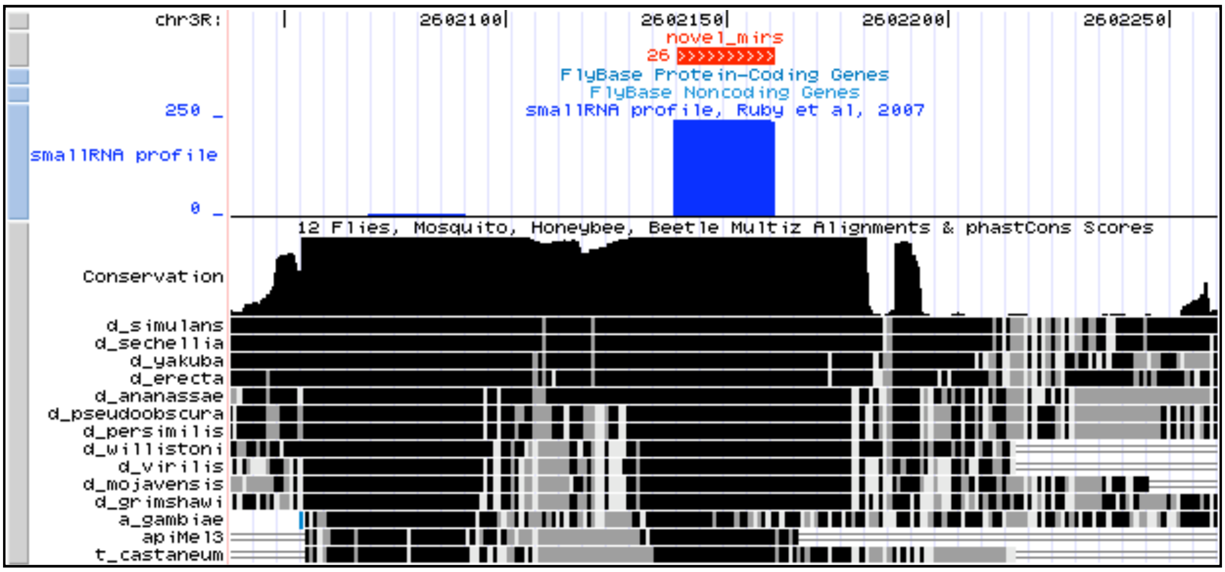

genomic context

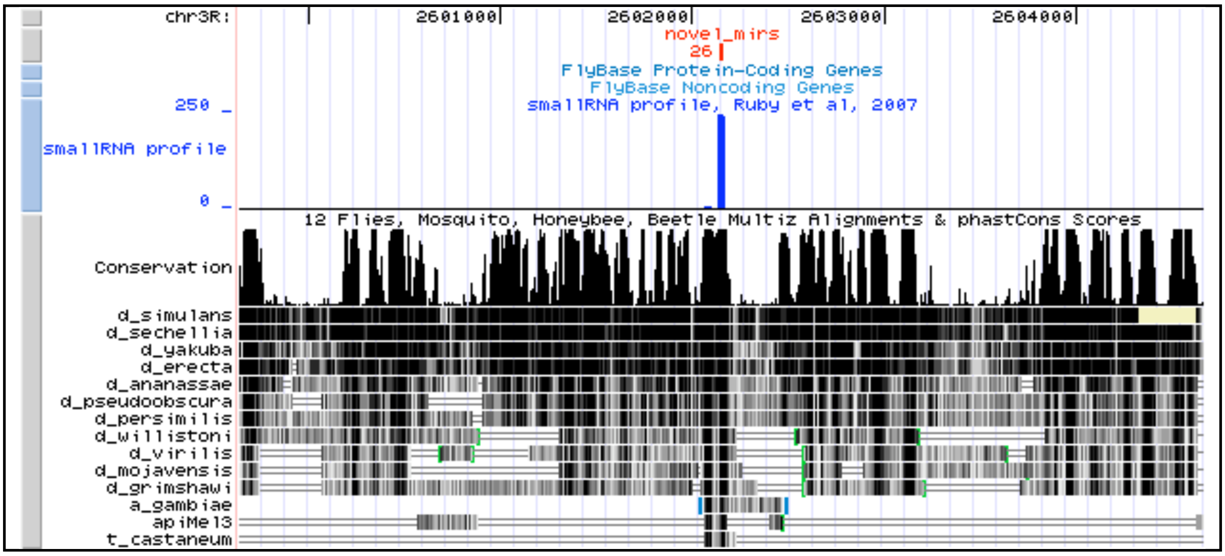

rnafold / alifold prediction

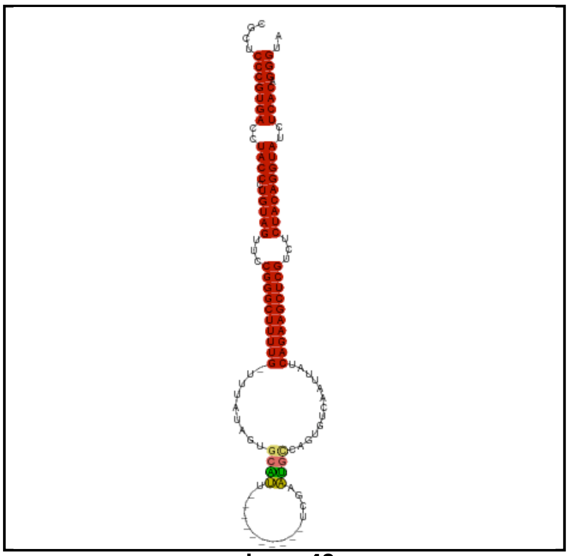

locus48+

Hits in RNA libraries

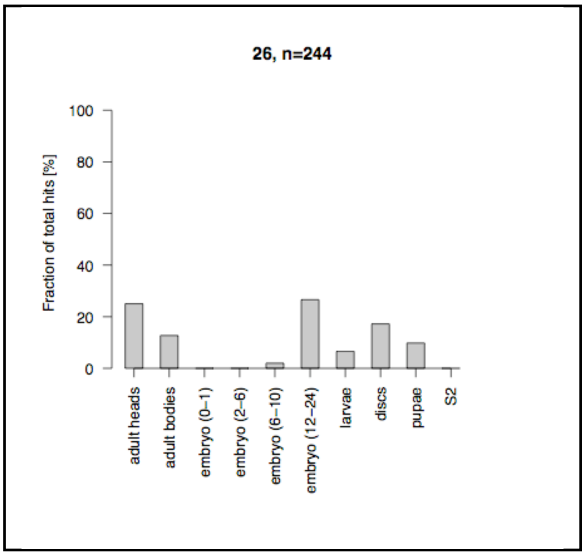

miRNA 27

chr3R : 6233870 - 6233892 Transcribed strand: +

cluster2 with dme-miR-318

predicted as: locus43

Validated: detected in small RNA sequencing data, validated by Northern blot

Mature sequence: CUAAGGAAAUAGUAGCCGUGAU

conservation profile

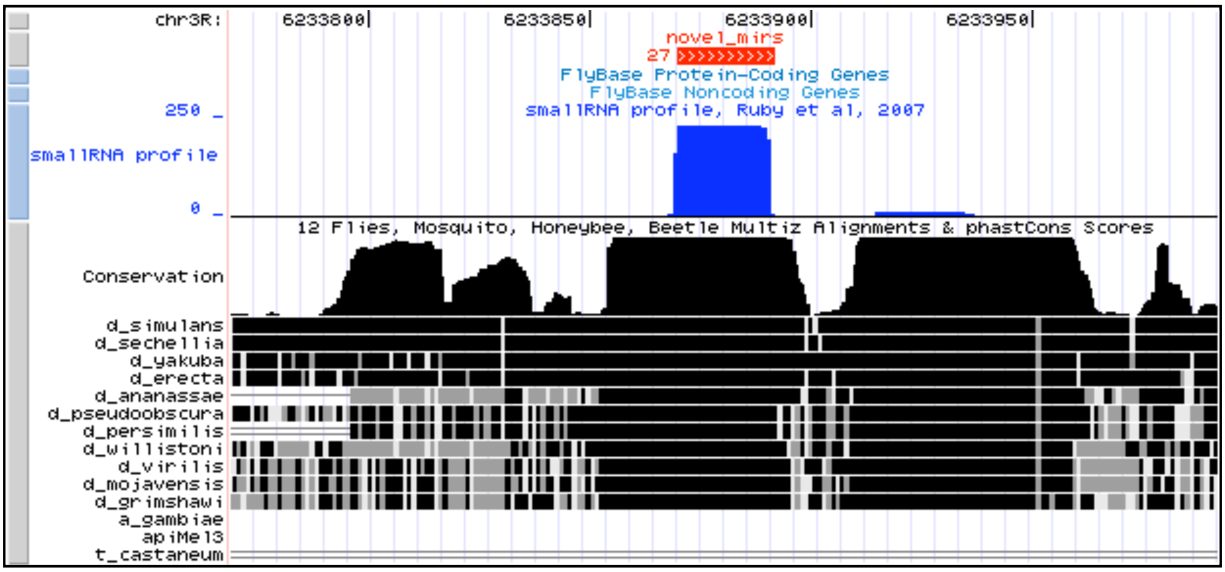

genomic context

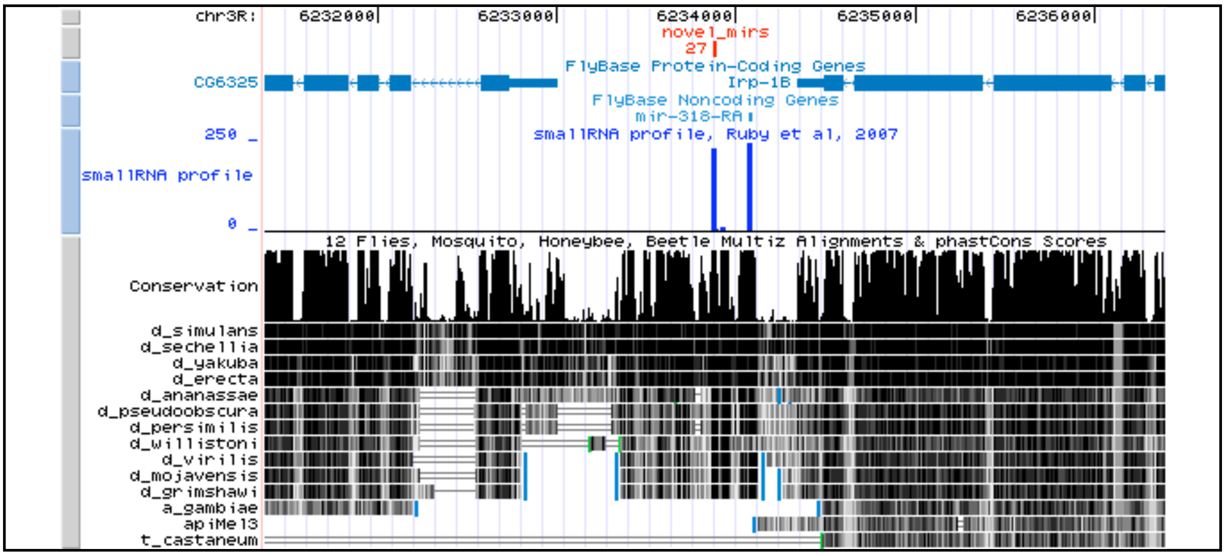

rnafold / alifold prediction

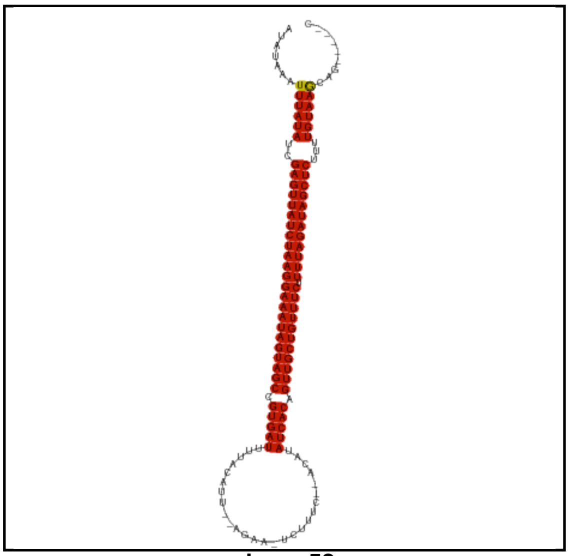

locus52+

Hits in RNA libraries

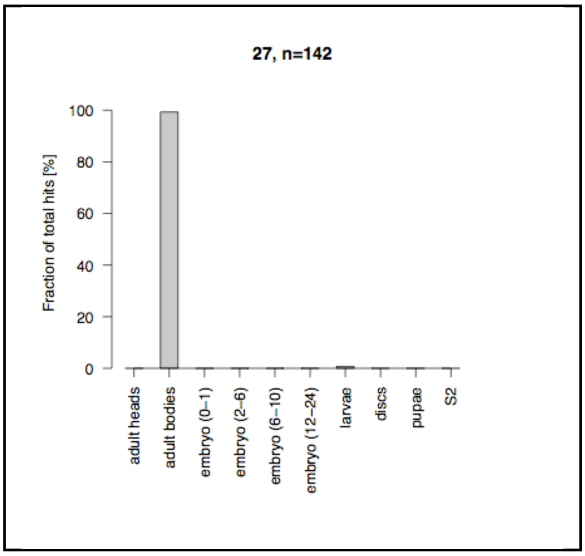

miRNA 28

chr3R : 9289996 - 9290018 Transcribed strand: -

predicted as:

Validated: detected in small RNA sequencing data

Mature sequence: CUAAGUACUAGUGCCGCAGGAG

conservation profile

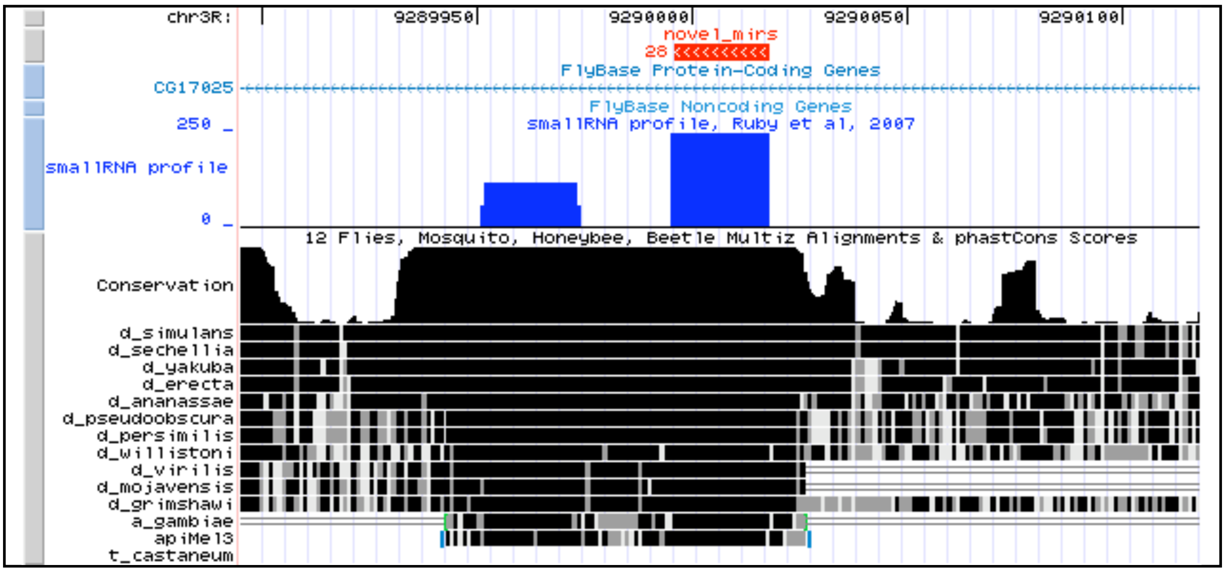

genomic context

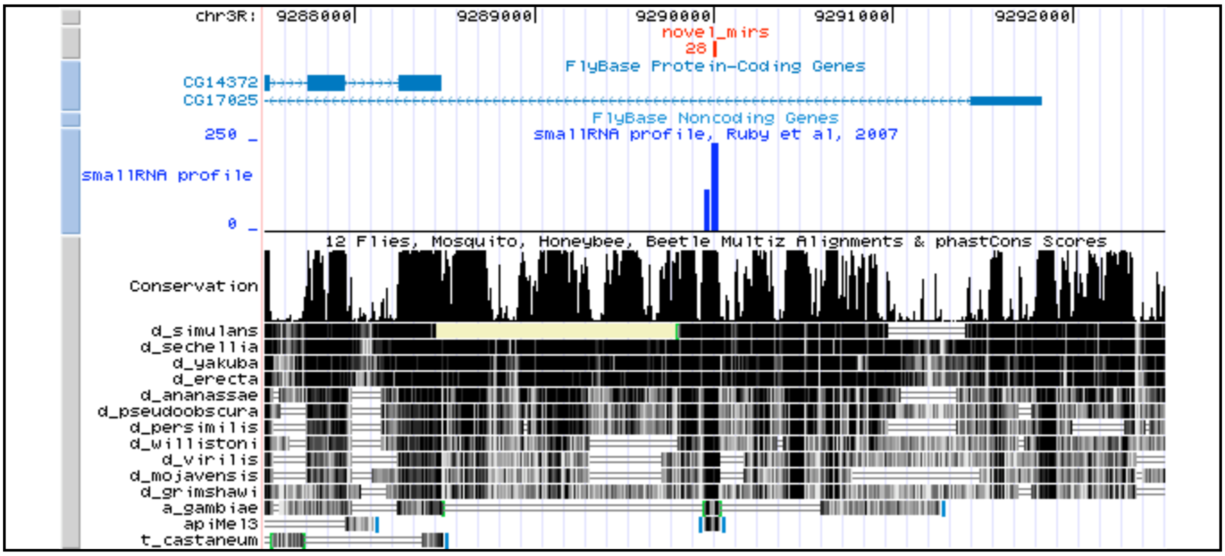

rnafold / alifold prediction

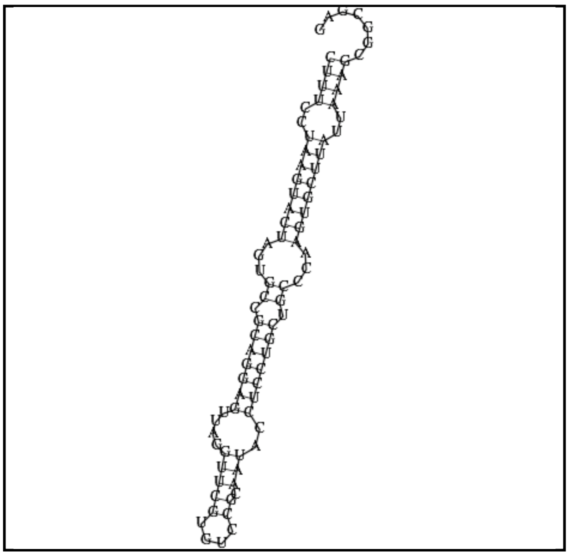

Hits in RNA libraries

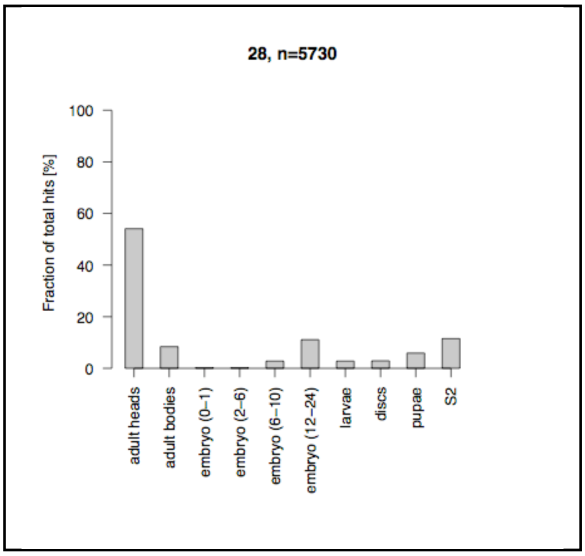

miRNA 29

chr3R : 16561699 - 16561719 Transcribed strand: +

predicted as:

Validated: detected in small RNA sequencing data

Mature sequence: UAGCACCACAUGAUUCGGCUU

conservation profile

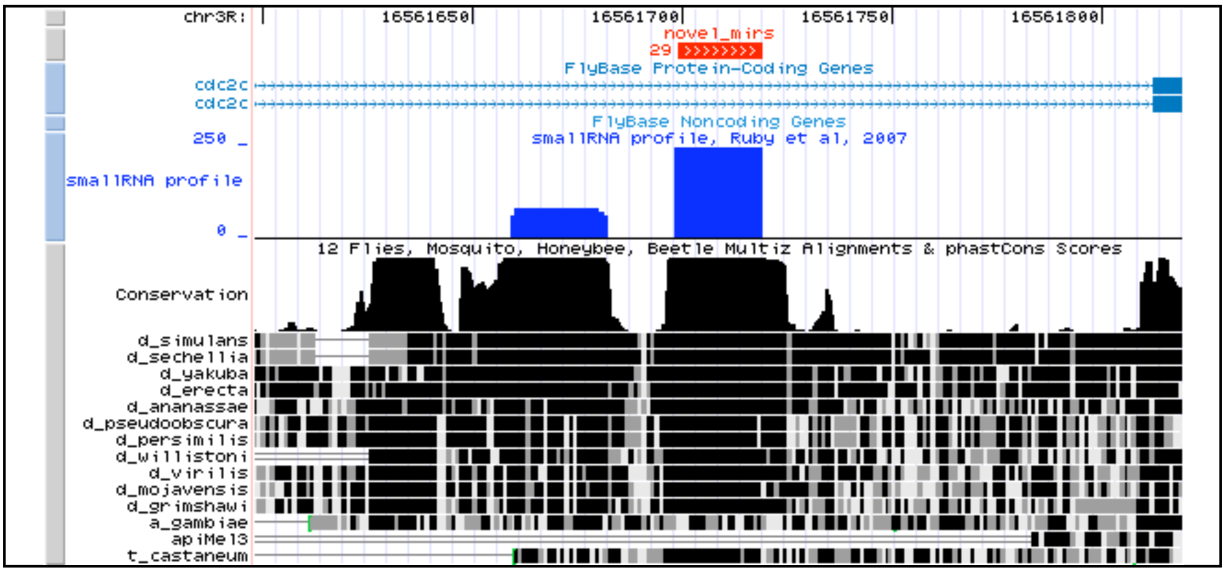

genomic context

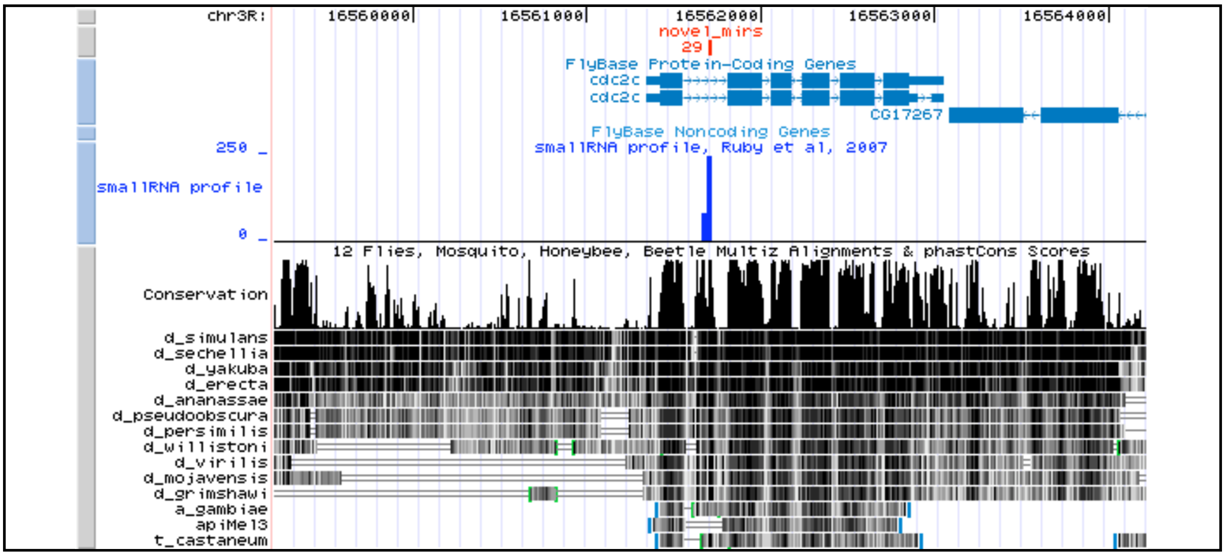

rnafold / alifold prediction

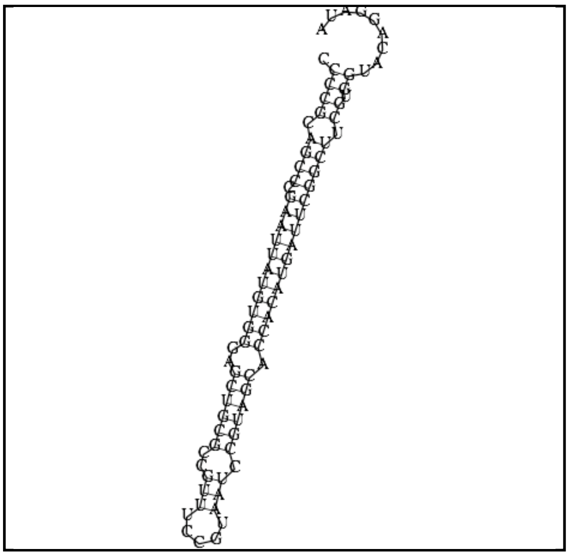

Hits in RNA libraries

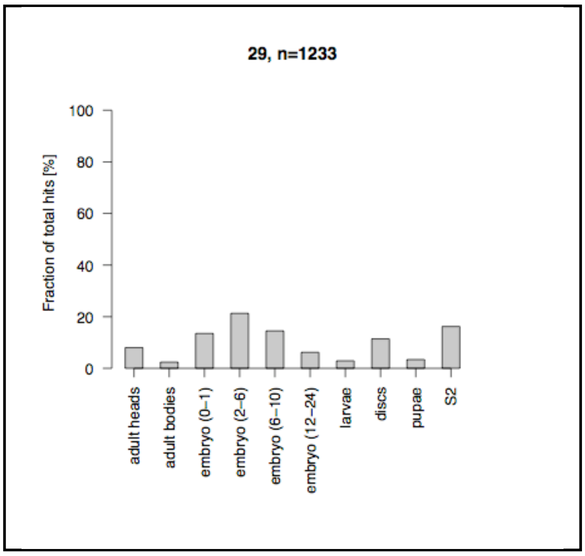

miRNA 30 chr3R : 16738582 - 16738600 Transcribed strand: -

predicted as:

Validated: detected in small RNA sequencing data

Mature sequence: AUGCGAUUUCAUCAUGGCA

conservation profile

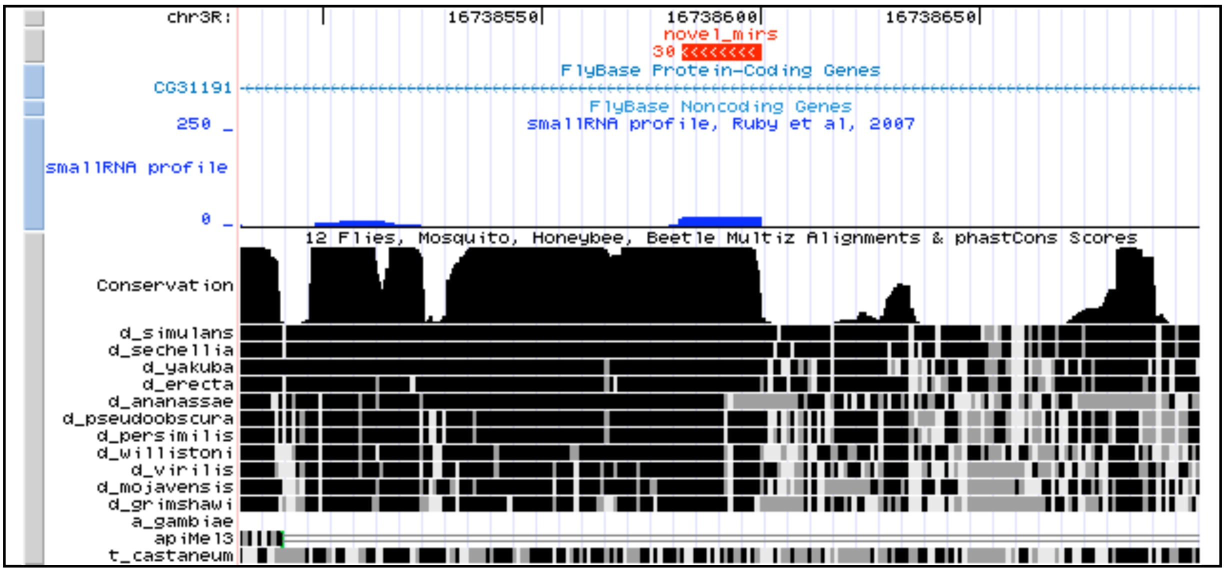

genomic context

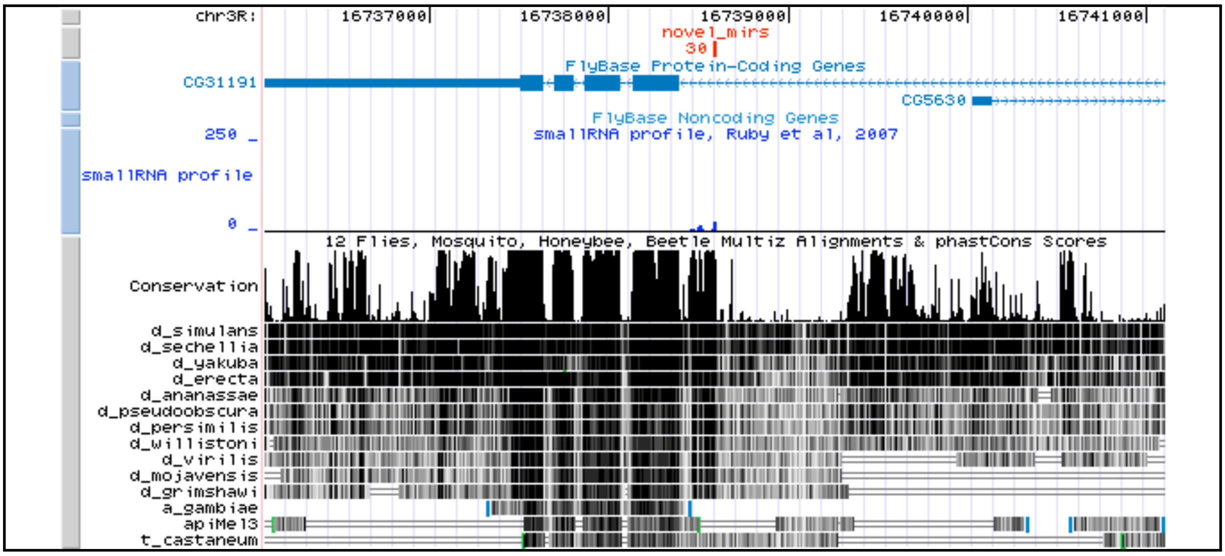

rnafold / alifold prediction

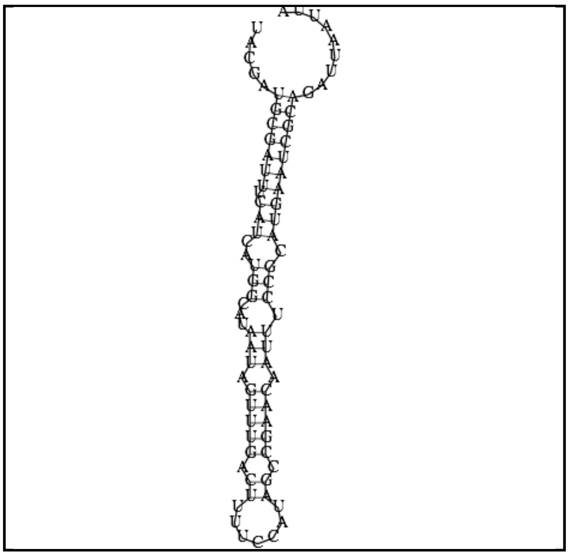

Hits in RNA libraries

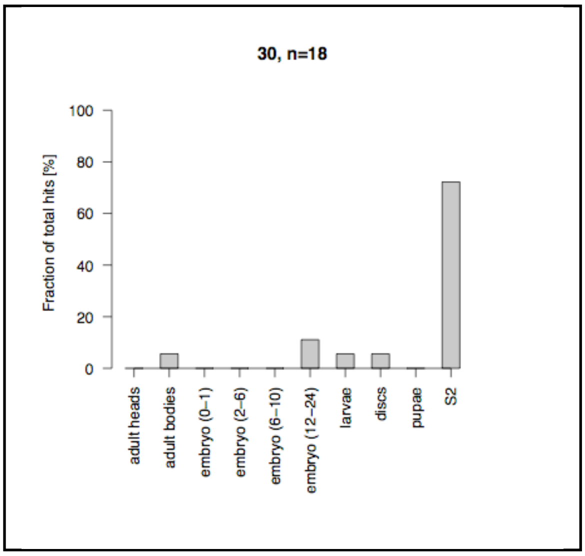

miRNA 31

chr3R : 17447624 - 17447644 Transcribed strand: -

predicted as:

Validated: detected in small RNA sequencing data

Mature sequence: UAGCACCAUGAGAUUCAGCUC

conservation profile

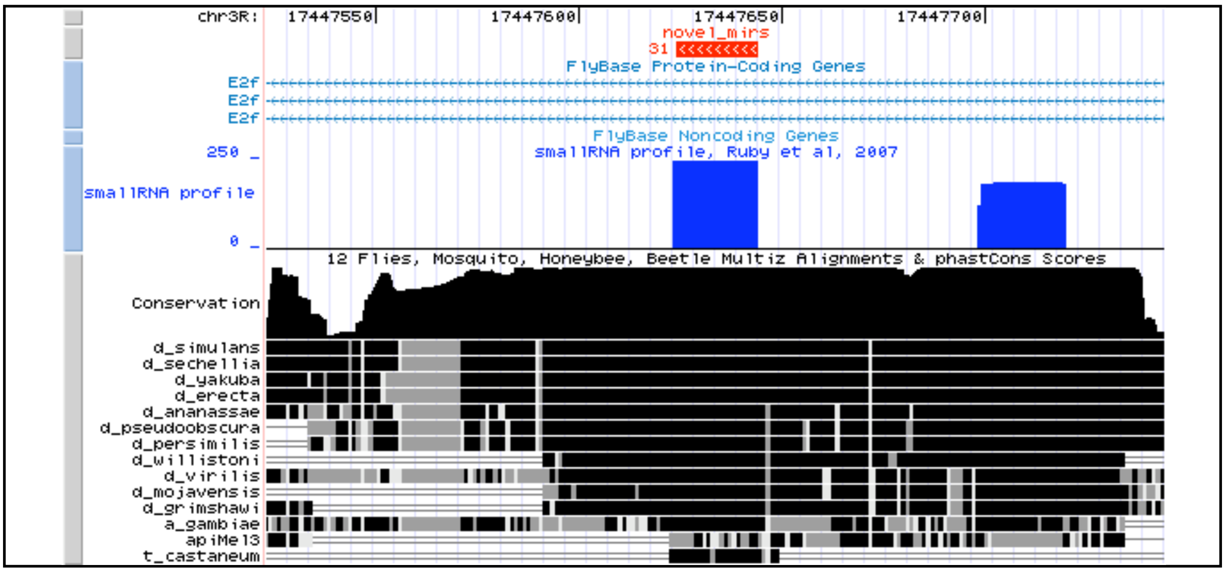

genomic context

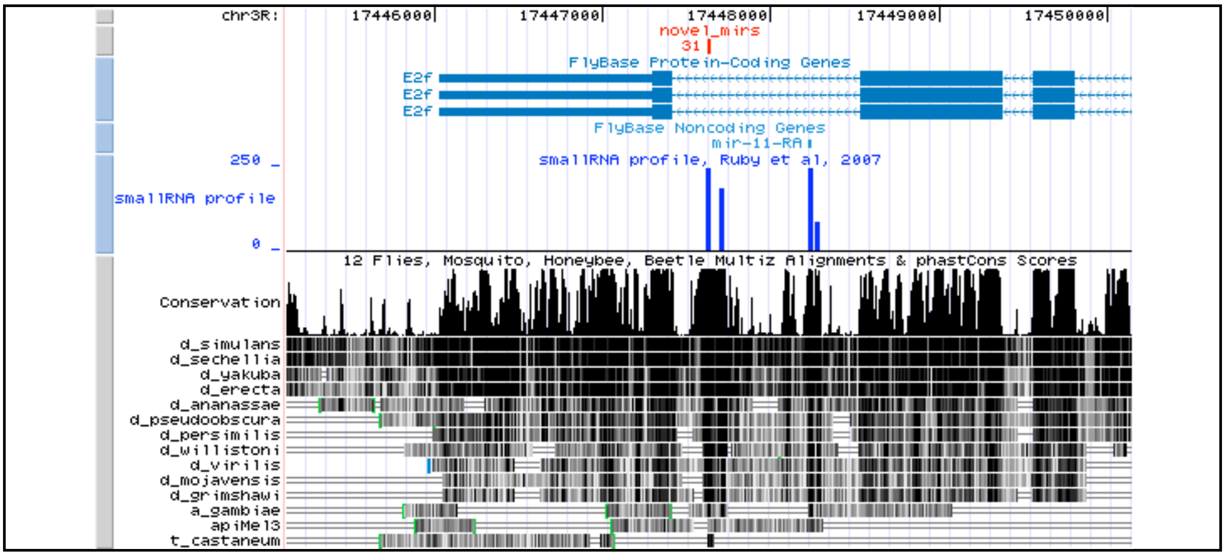

rnafold / alifold prediction

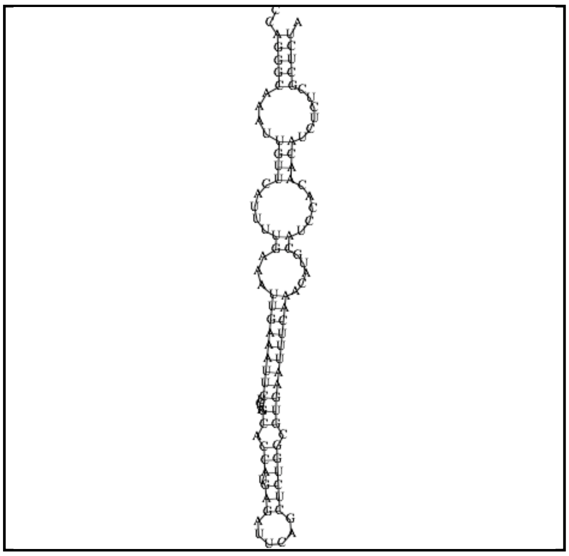

Hits in RNA libraries

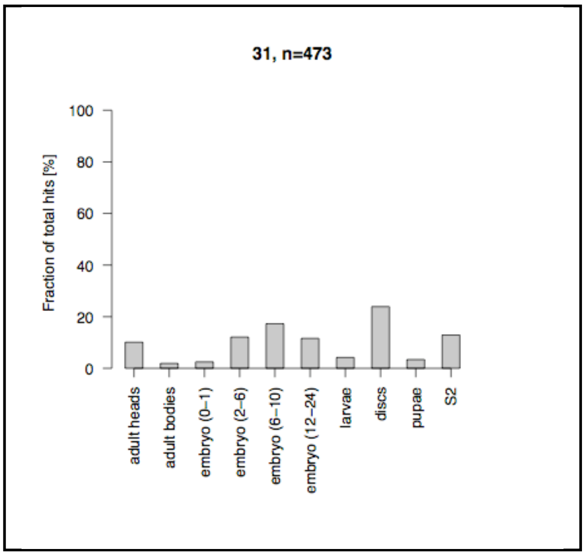

miRNA 32

chr3R : 17623971 - 17623992 **Transcribed strand: -**

predicted as:

Validated: **detected in small RNA sequencing data**

Mature sequence: **UGUUAACUGUAAGACUGUGUCU**

conservation profile

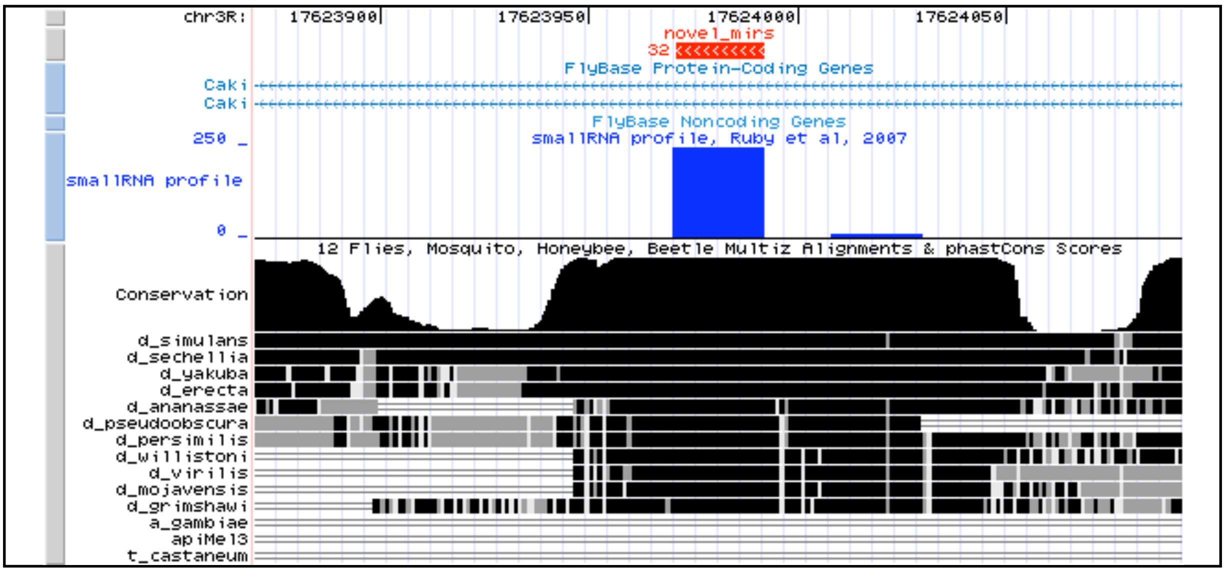

genomic context

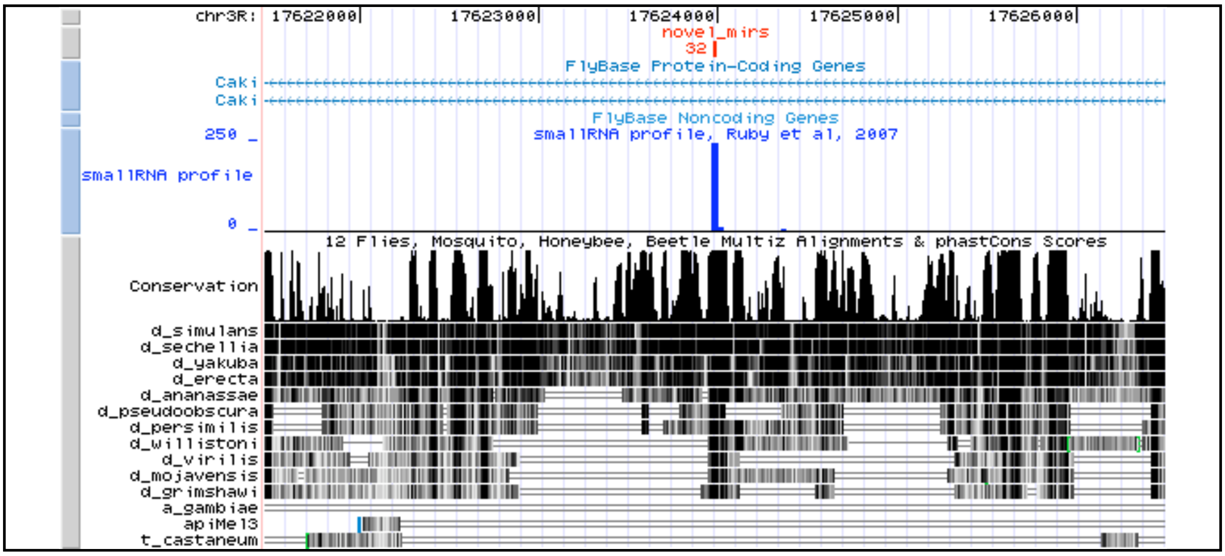

rnafold / alifold prediction

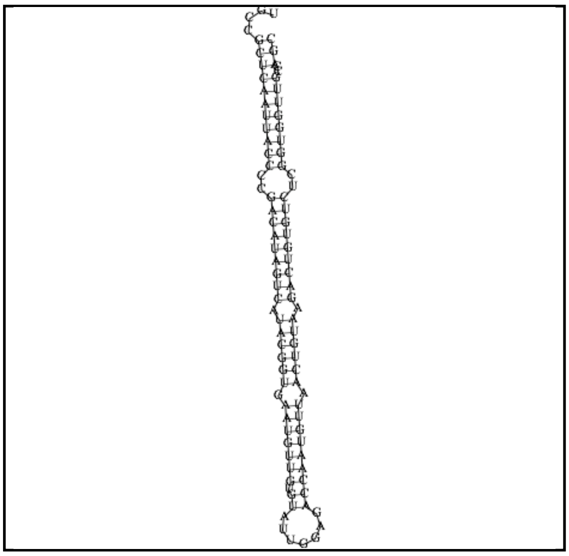

Hits in RNA libraries

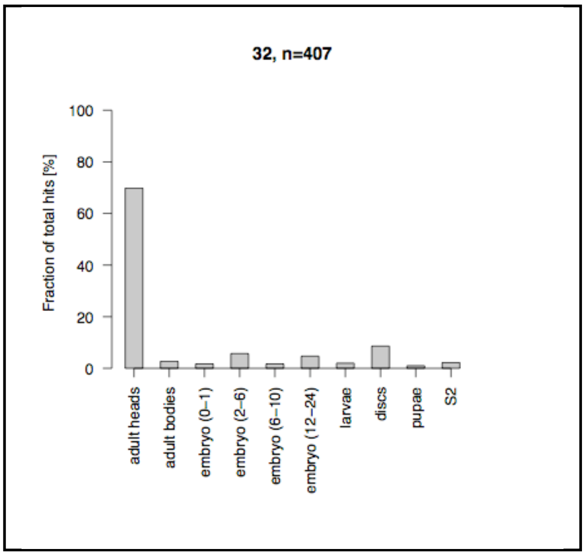

miRNA 33

chr3R : 21414645 - 21414666 Transcribed strand: -

predicted as: locus13

Validated: detected in small RNA sequencing data, validated by Northern blot

Mature sequence: AUAUUGUCCUGUCACAGCAGU

conservation profile

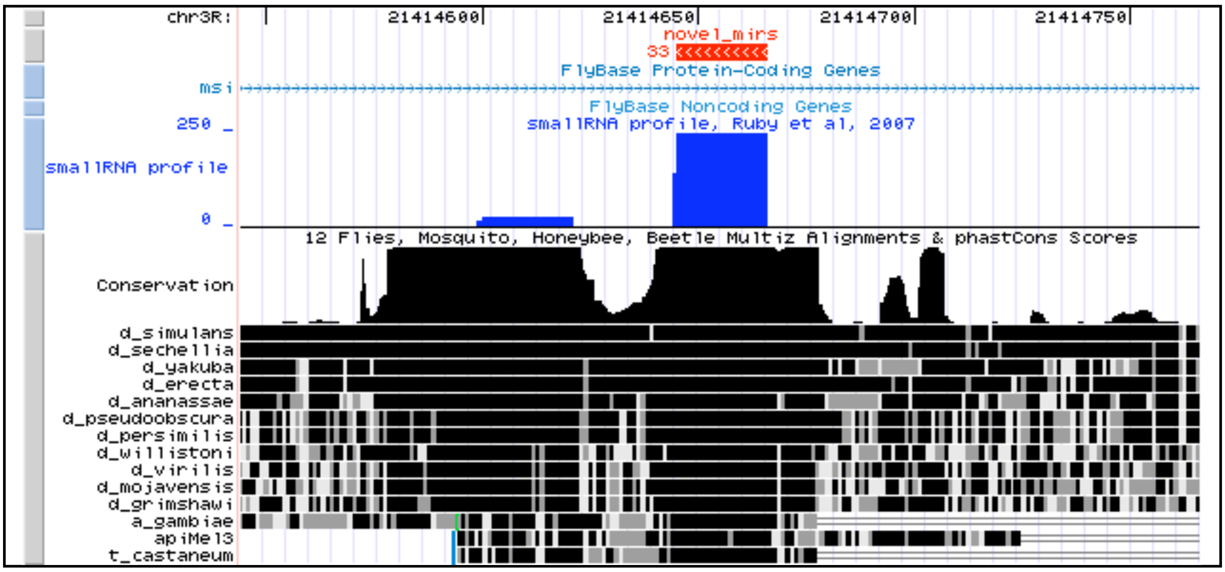

genomic context

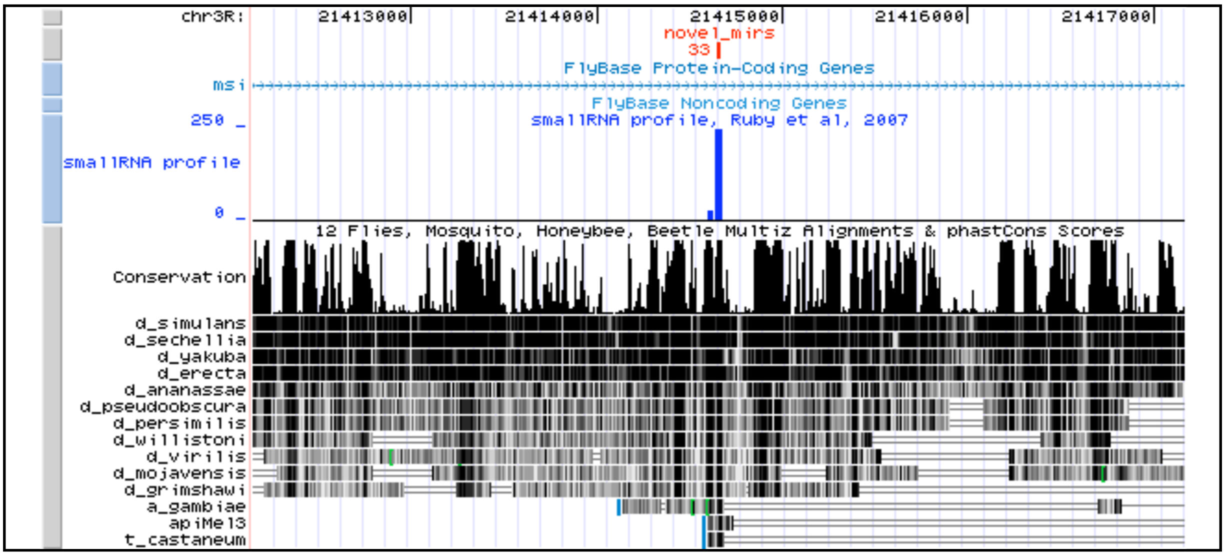

rnafold / alifold prediction

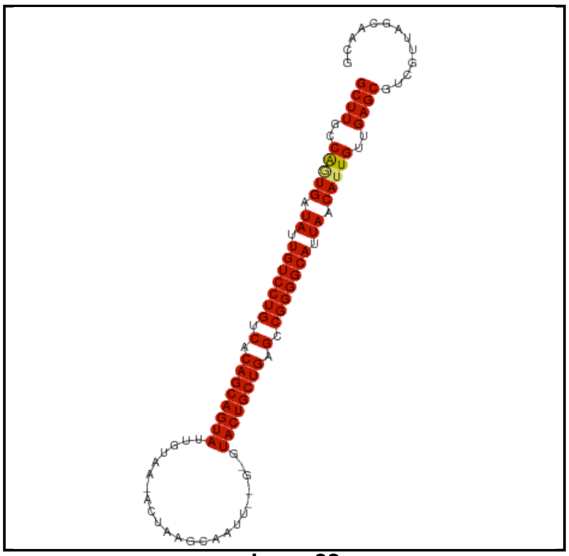

locus62-

Hits in RNA libraries

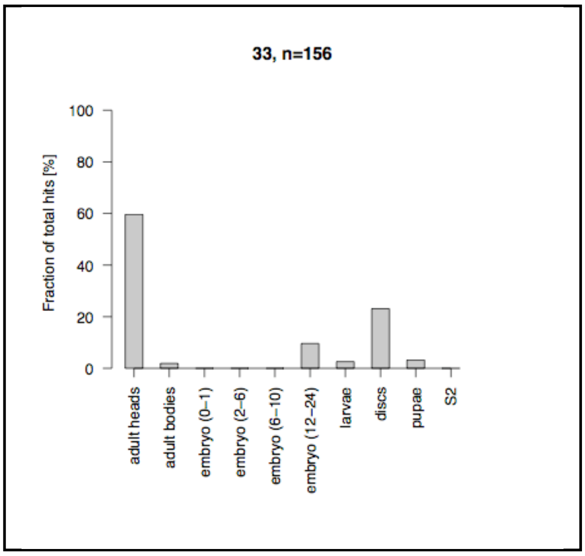

miRNA 34

chr3R : 23468242 - 23468262   Transcribed strand: -

predicted as:

Validated: detected in small RNA sequencing data

Mature sequence: UGGGUAAACUCCCAAGGAUCA

conservation profile

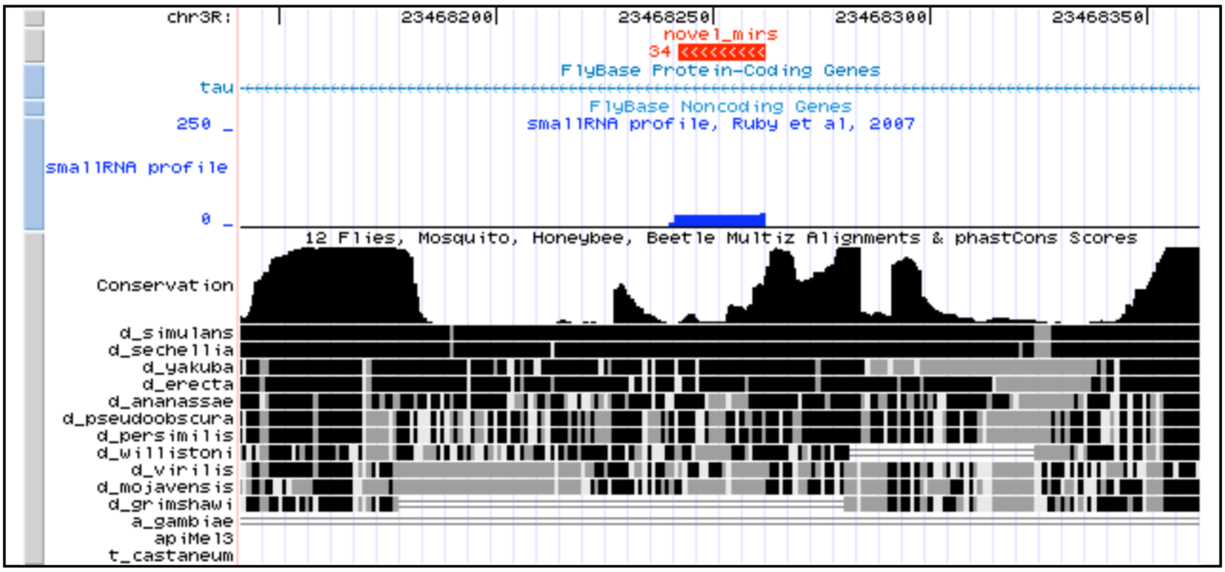

genomic context

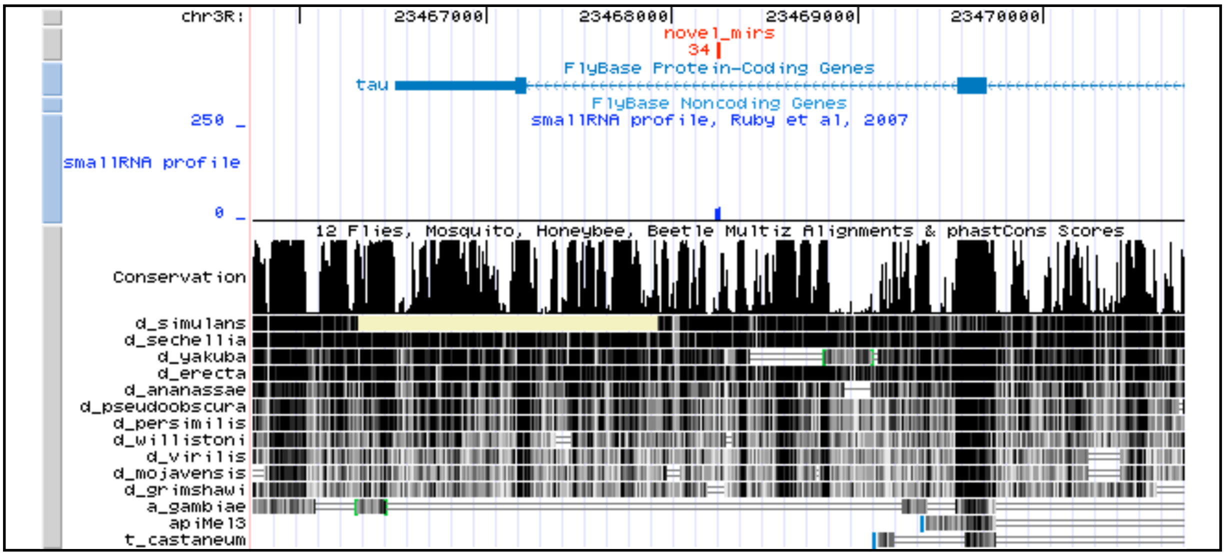

rnafold / alifold prediction

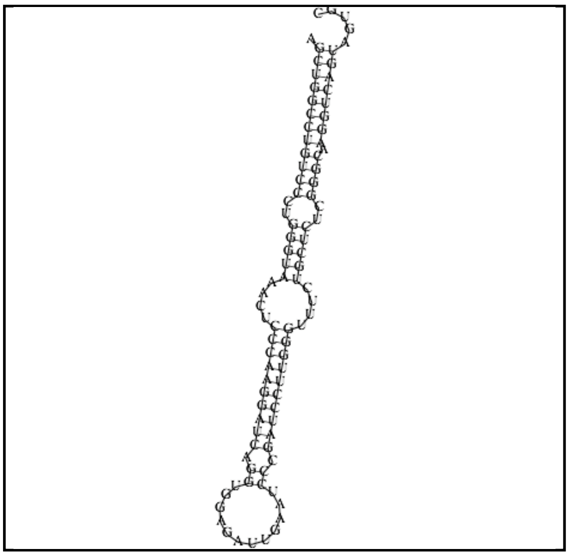

Hits in RNA libraries

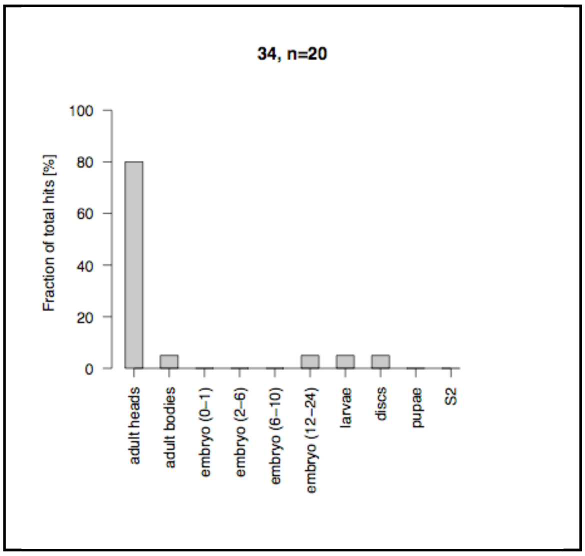

miRNA 35

chr3R : 24628381 - 24628452 Transcribed strand: -

predicted as: locus7

Validated: validated by Northern blot only

Mature sequence: n.d.,sequence matched by Northern probe: AACGACACCAAAUCAAUGCUC

conservation profile

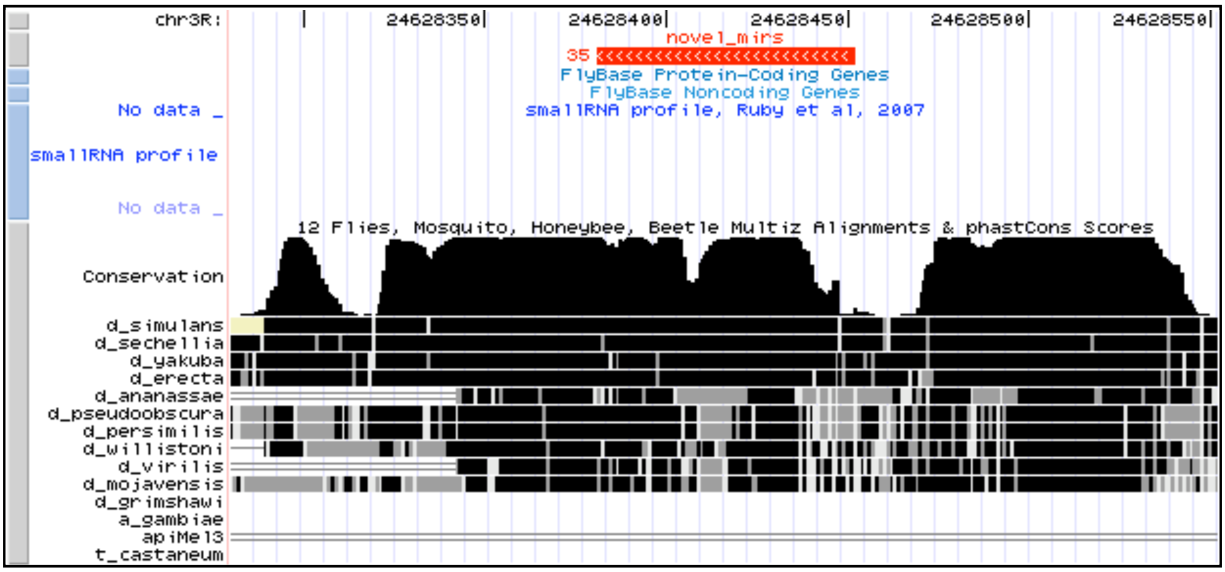

genomic context

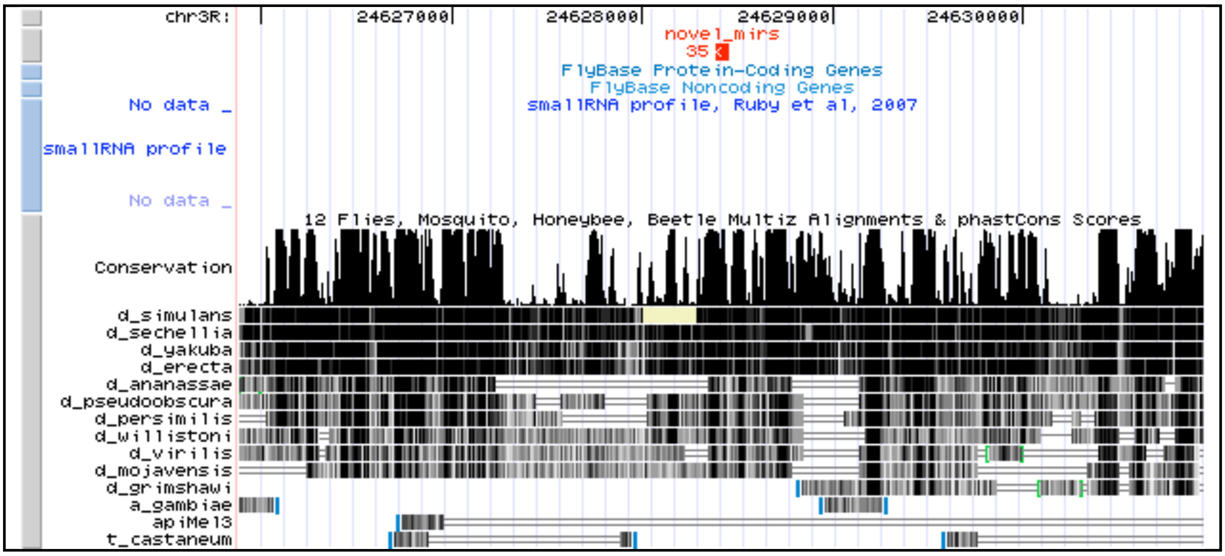

rnafold / alifold prediction

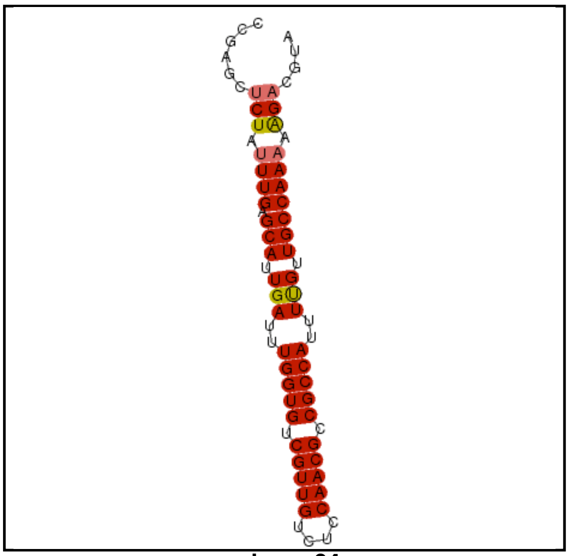

locus64-

Hits in RNA libraries

miRNA 36

chr3R : 25042965 - 25042989 Transcribed strand: +

predicted as:

Validated: detected in small RNA sequencing data

Mature sequence: UGACUAGAUUUCAUGCUCGUCU

conservation profile

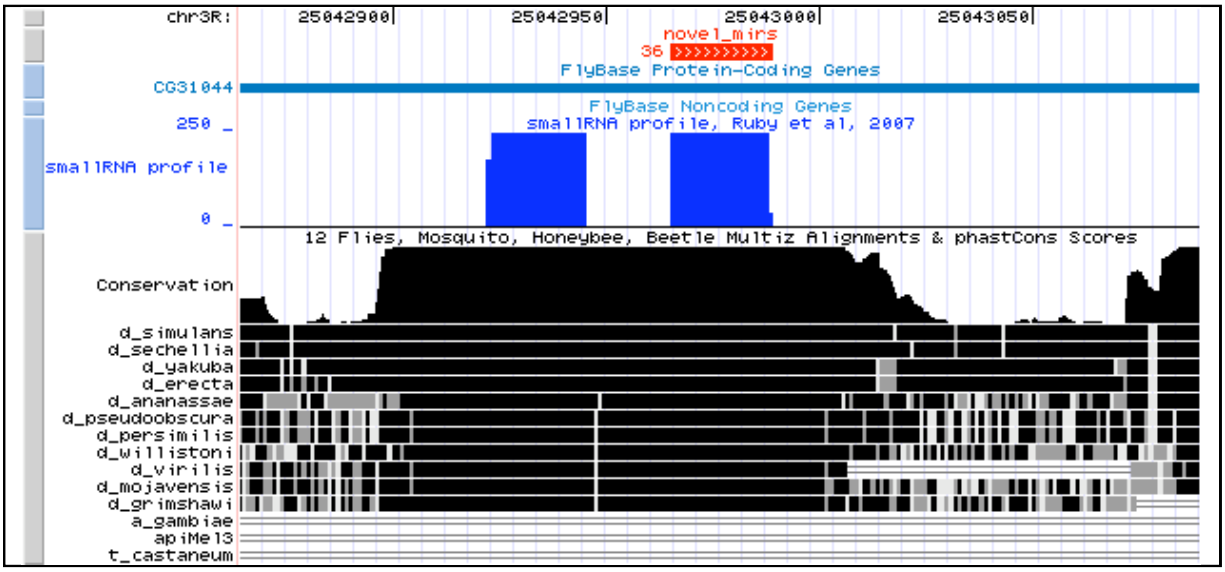

genomic context

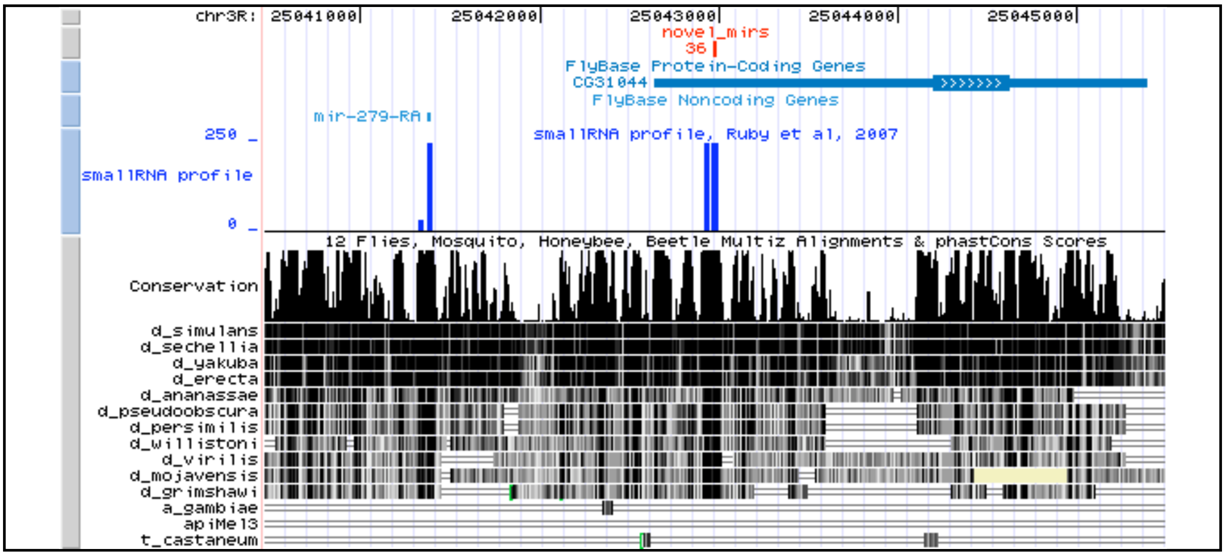

rnafold / alifold prediction

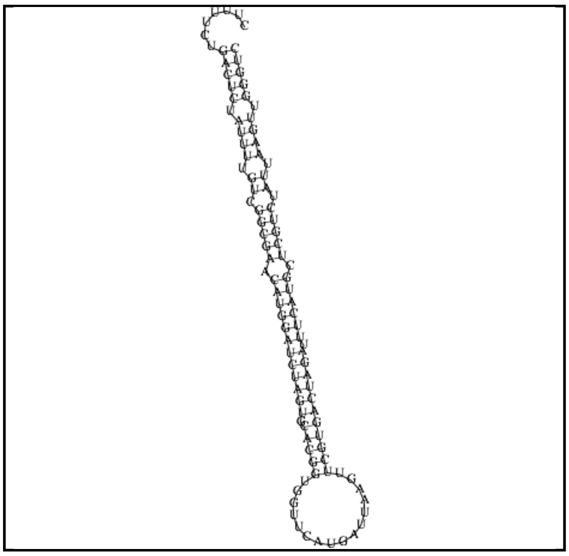

Hits in RNA libraries

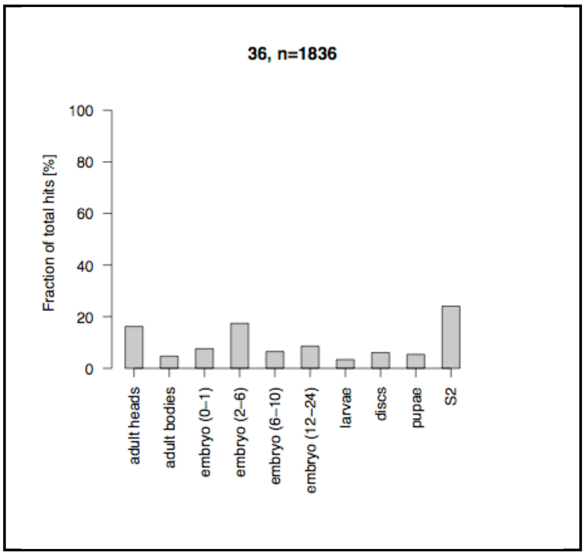

miRNA 37

chr4 : 646768 - 646788 Transcribed strand: +

predicted as:

Validated: detected in small RNA sequencing data

Mature sequence: UCUGGGUGUUGCGUUGUGUGU

conservation profile

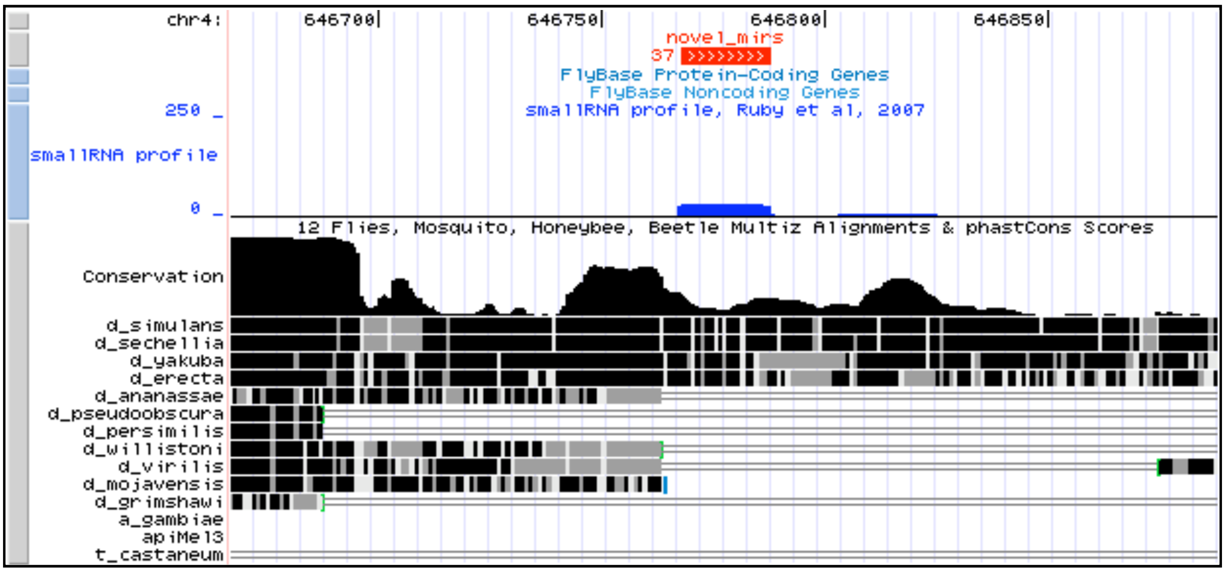

genomic context

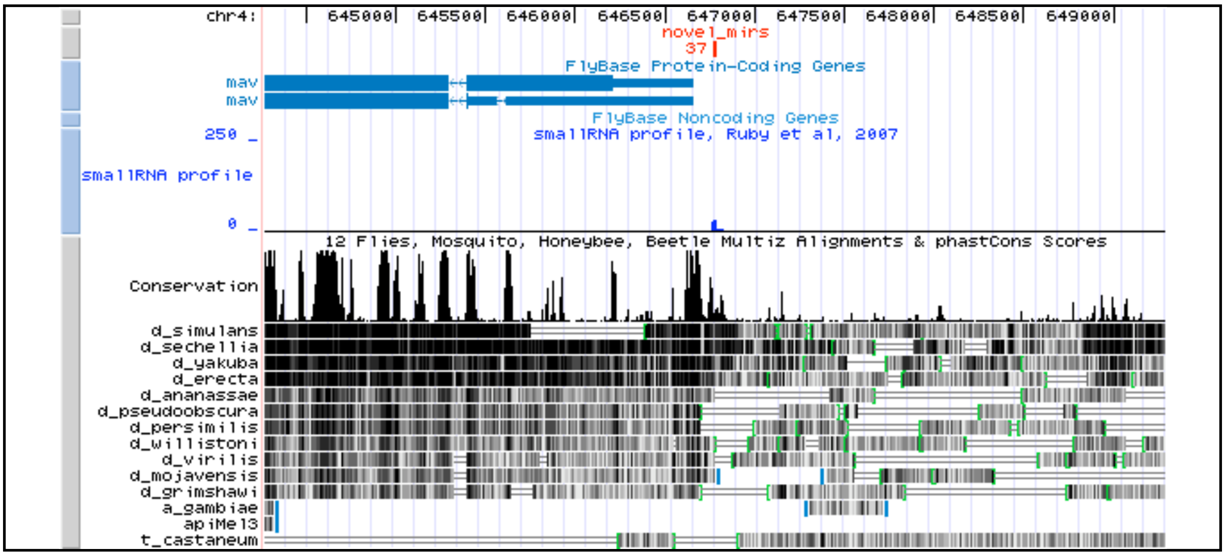

rnafold / alifold prediction

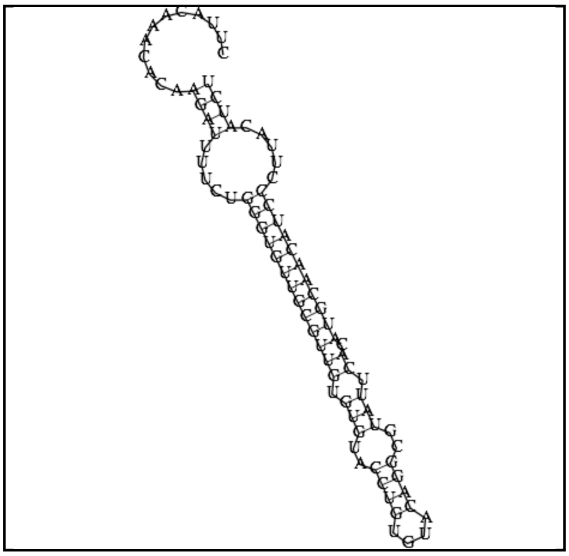

Hits in RNA libraries

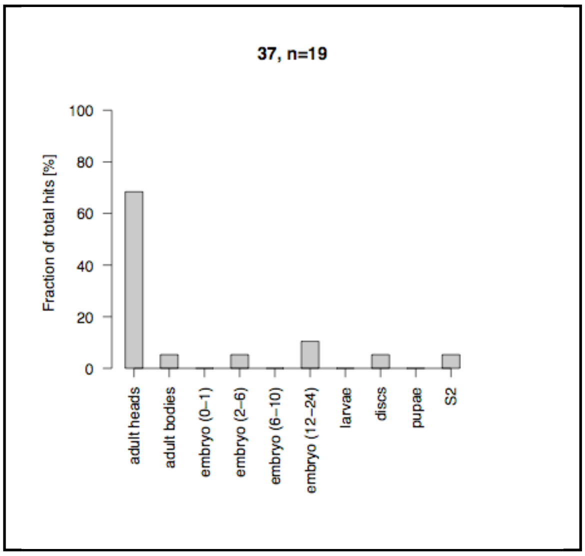

miRNA 38

chrX : 174221 - 174242 Transcribed strand: -

predicted as: locus11

Validated: detected in small RNA sequencing data, validated by Northern blot

Mature sequence: UAGCUGCCUUGUGAAGGGCUUA

conservation profile

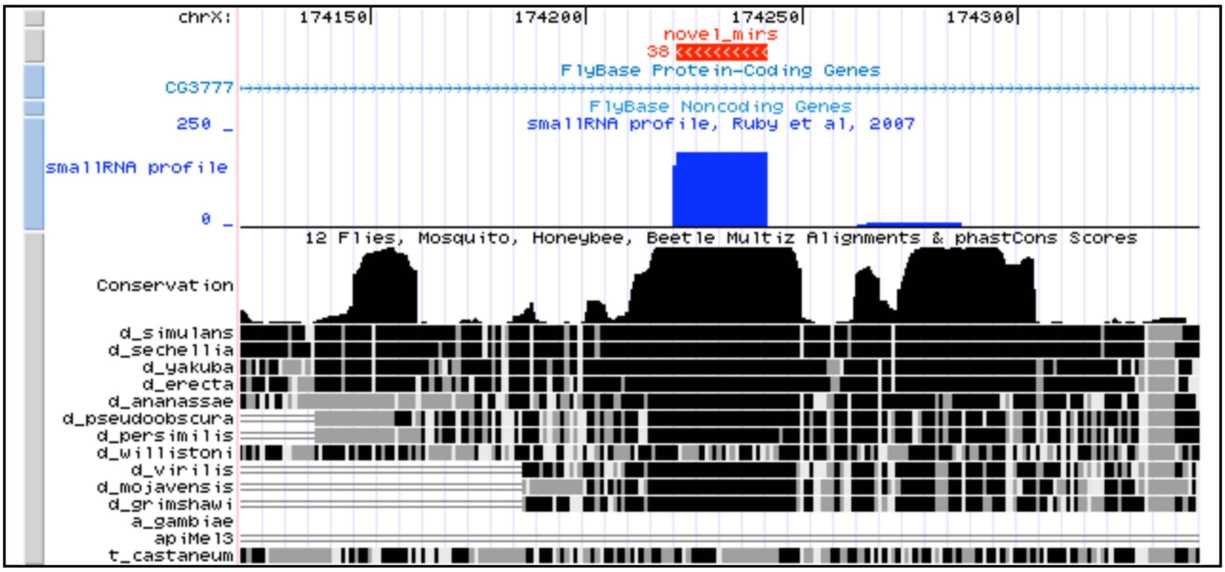

genomic context

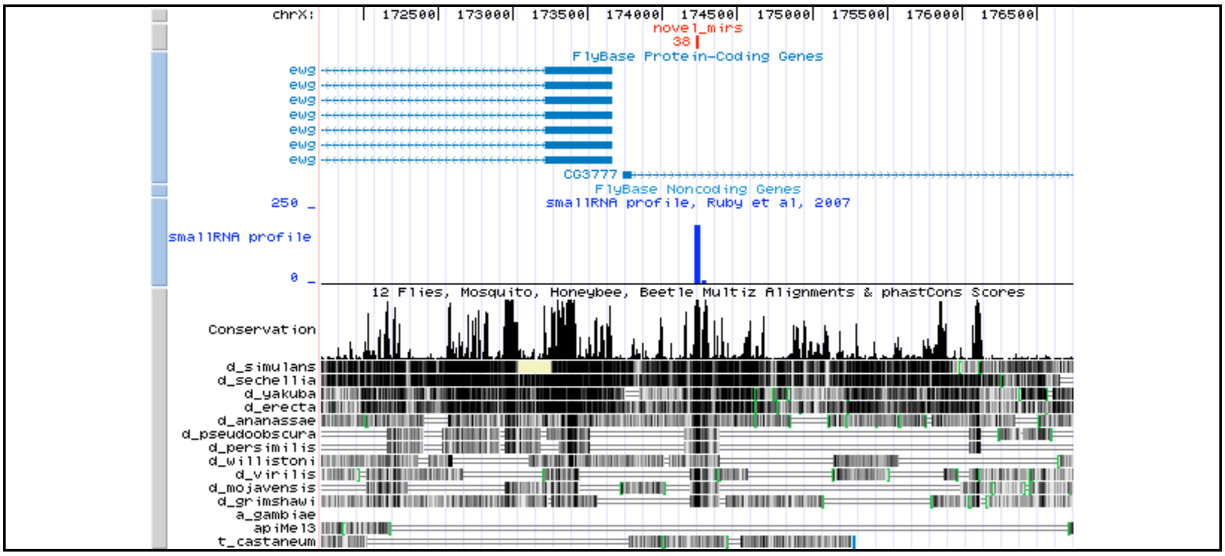

rnafold / alifold prediction

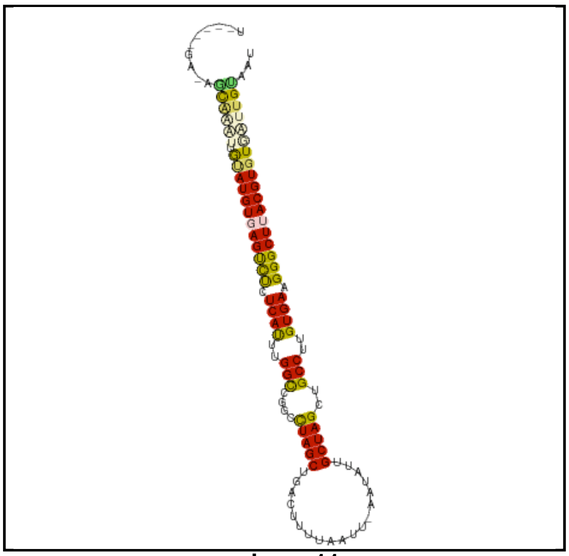

locus11-

Hits in RNA libraries

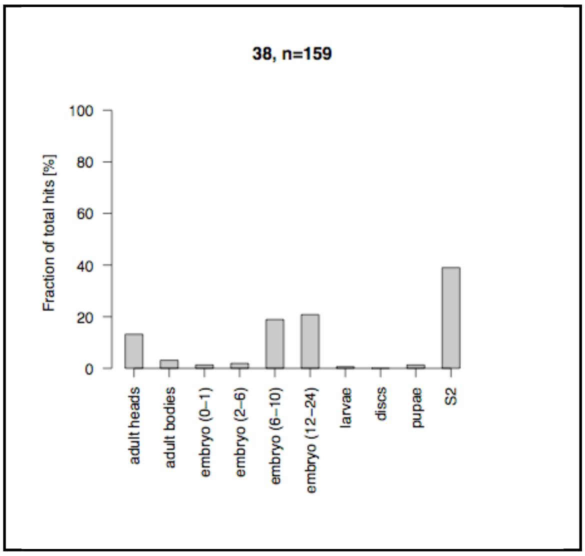

miRNA 39 chrX : 1682908 - 1682930 Transcribed strand: -

predicted as: locus12

Validated: detected in small RNA sequencing data, validated by Northern blot

Mature sequence: UUCGUUGUCGACGAAACCUGCA

conservation profile

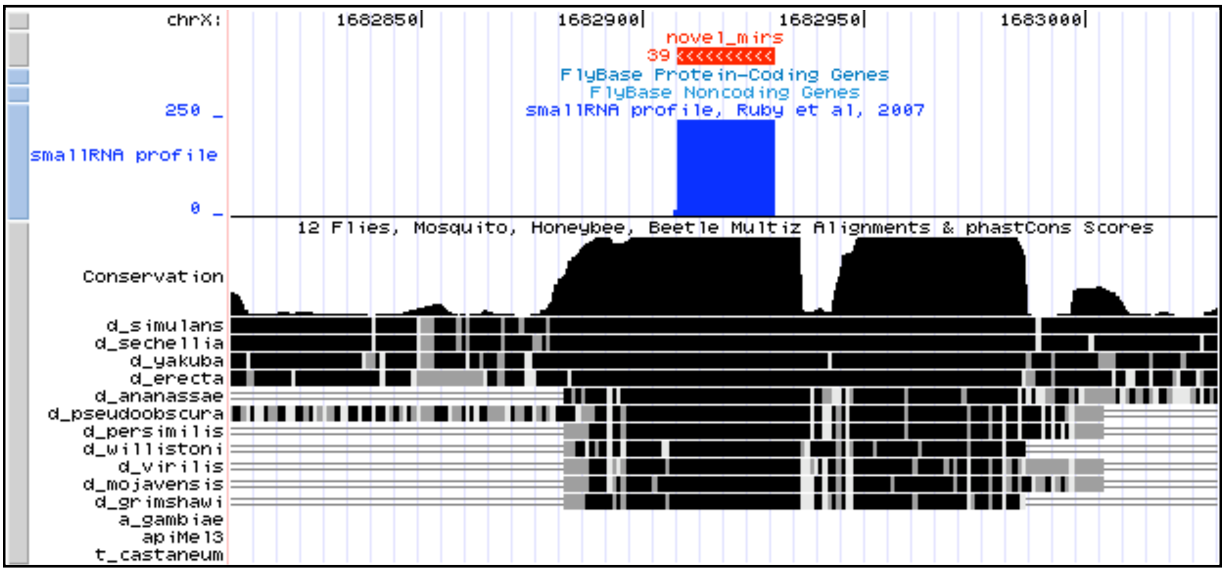

genomic context

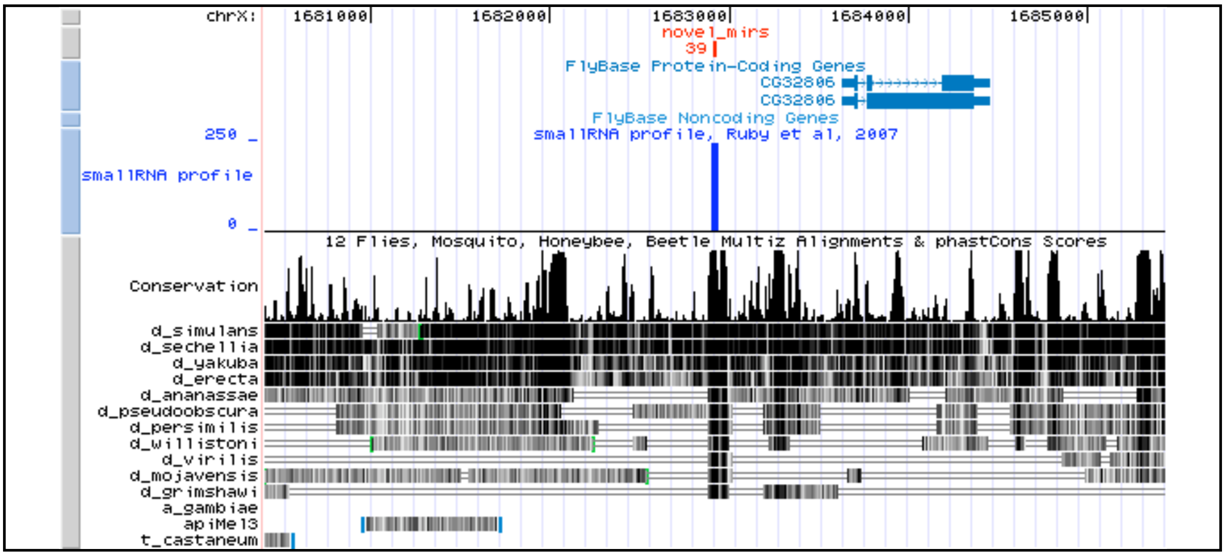

rnafold / alifold prediction

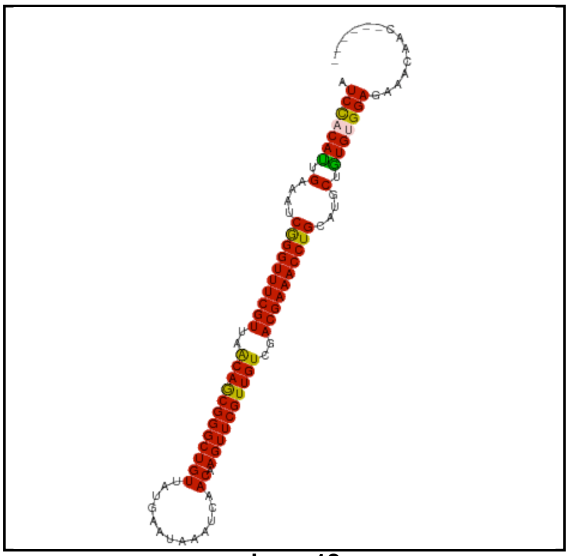

locus12-

Hits in RNA libraries

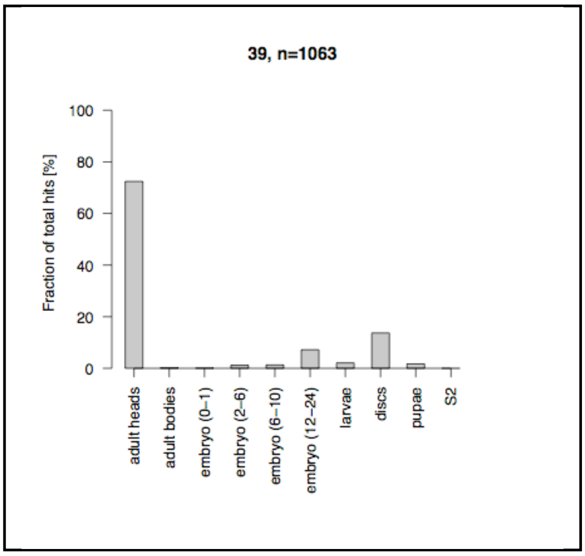

miRNA 40 chrX : 4262357 - 4262378 Transcribed strand: -

predicted as:

Validated: detected in small RNA sequencing data

Mature sequence: AUUAGGUAGUUACGCAUUAUCU

conservation profile

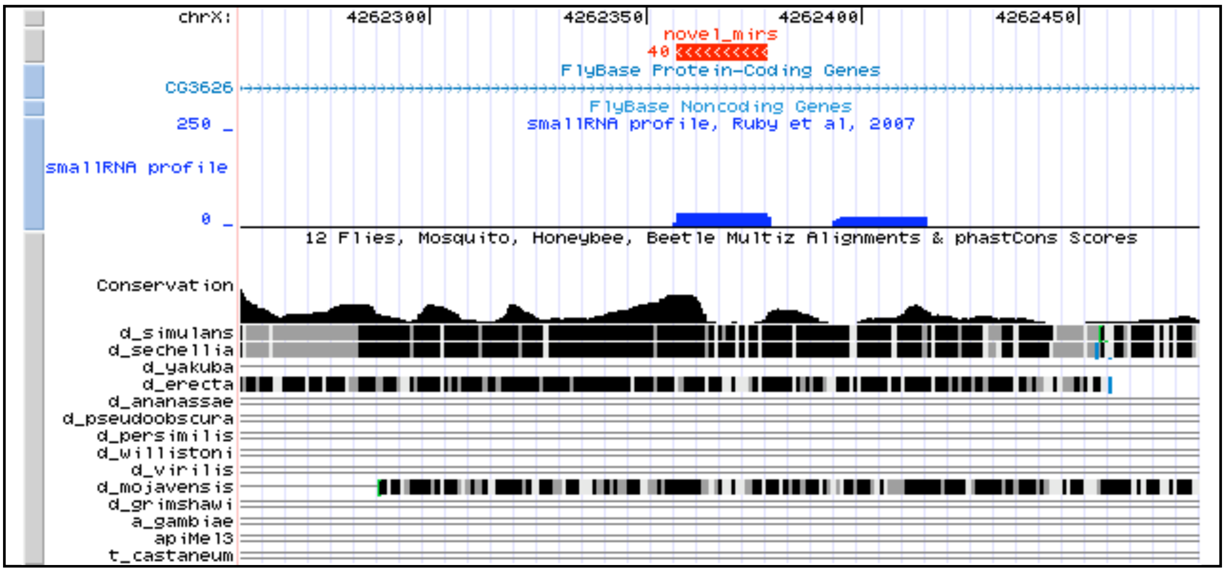

genomic context

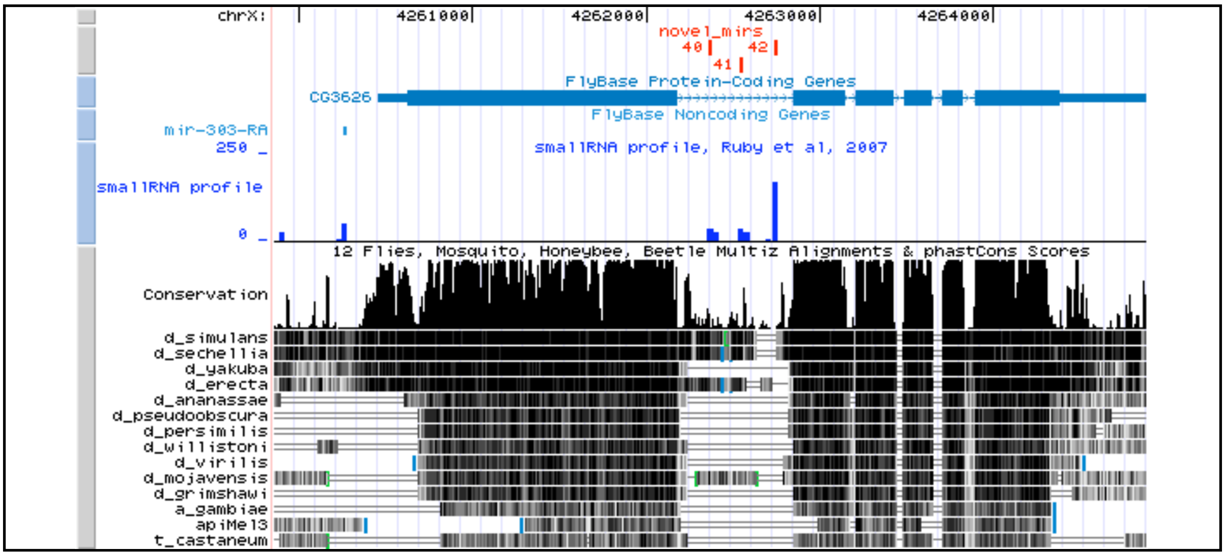

rnafold / alifold prediction

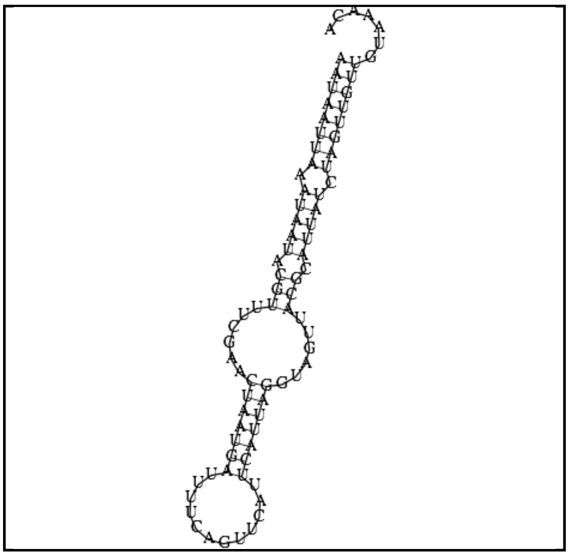

Hits in RNA libraries

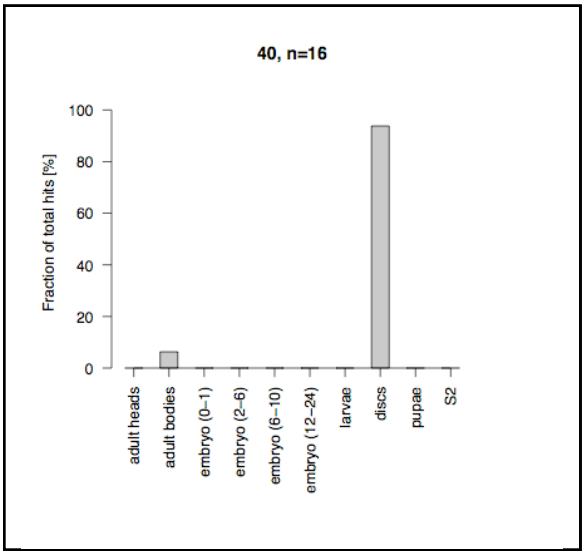

miRNA 41

chrX : 4262536 - 4262558   Transcribed strand: -

predicted as:

Validated: detected in small RNA sequencing data

Mature sequence: AUUAGGUAGUUACGCAUUAUCU

conservation profile

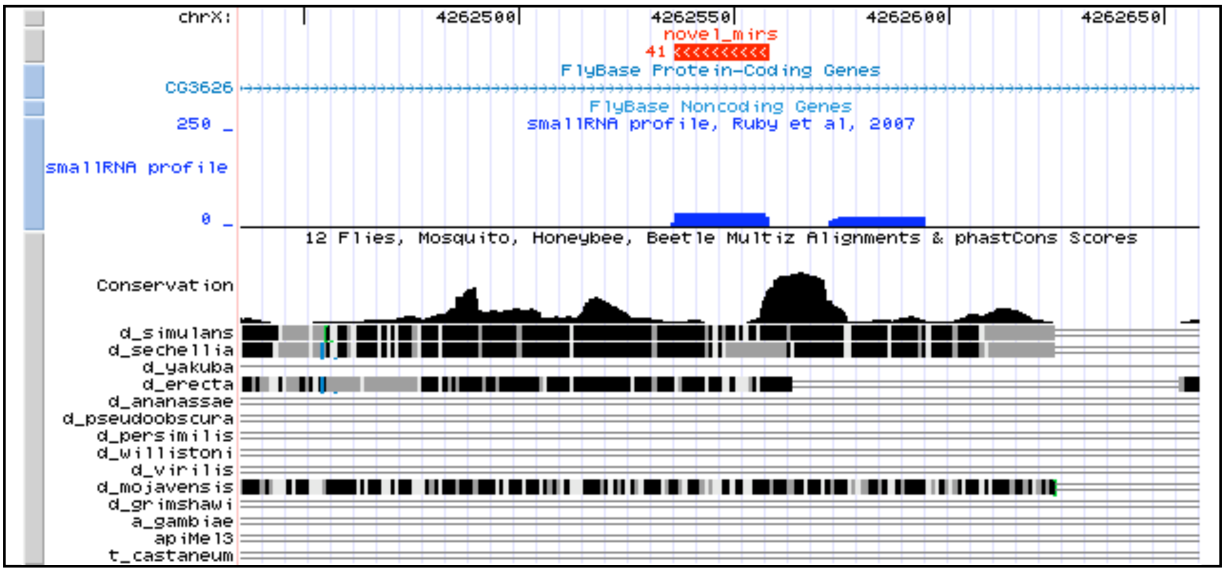

genomic context

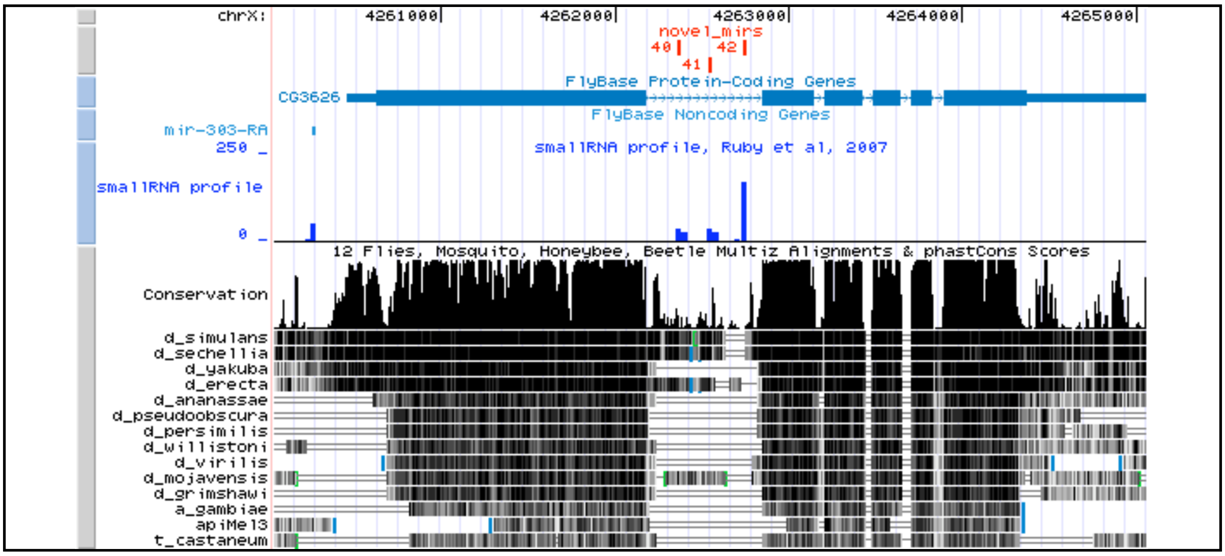

rnafold / alifold prediction

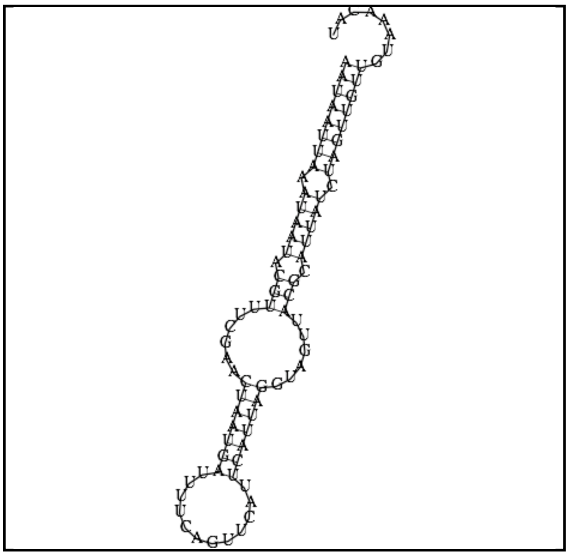

Hits in RNA libraries

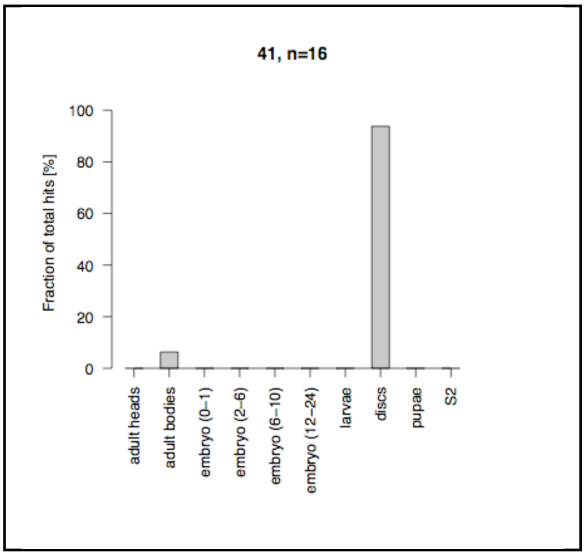

miRNA 42

chrX : 4262731 - 4262753 Transcribed strand: -

predicted as:

Validated: detected in small RNA sequencing data

Mature sequence: UGAGGUAAAUACGGUUGGAAUUU

conservation profile

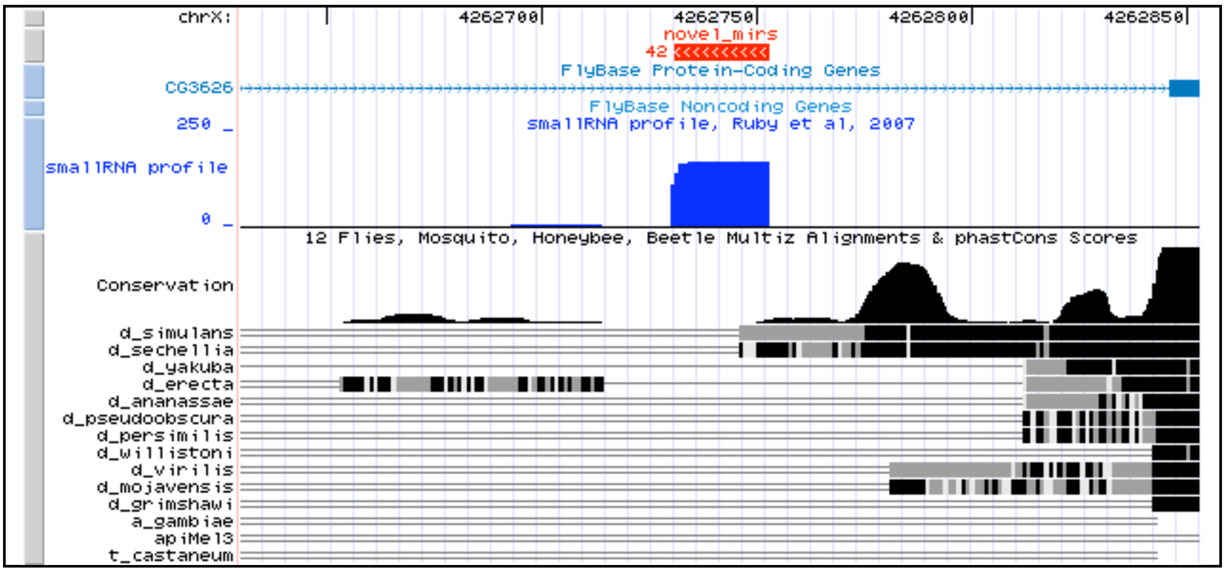

genomic context

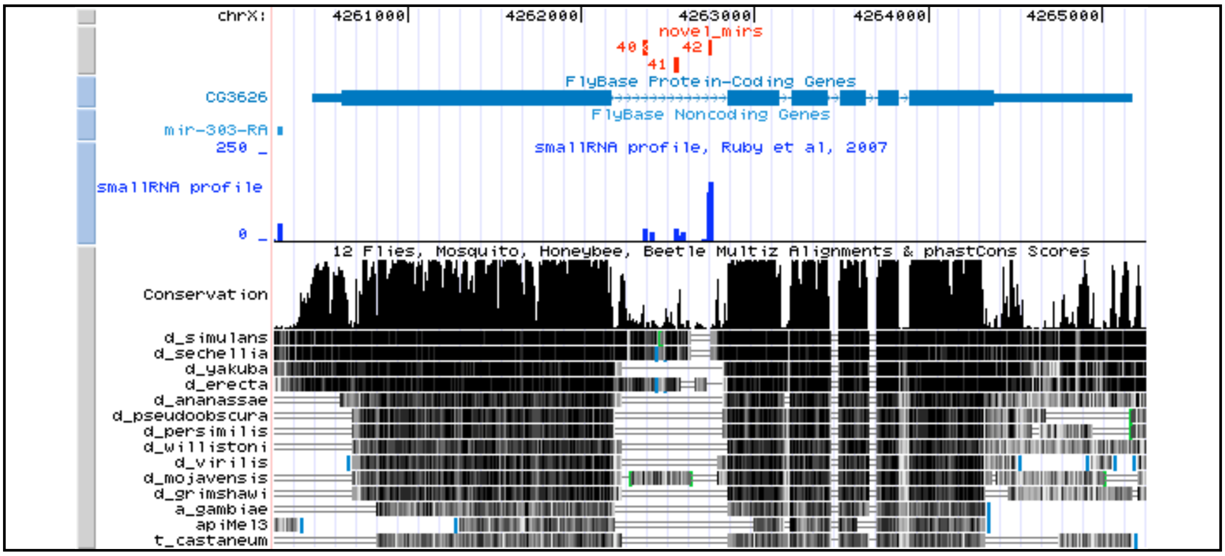

rnafold / alifold prediction

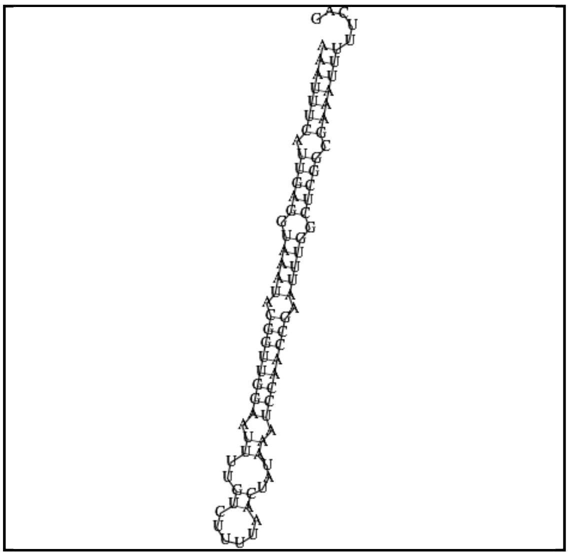

Hits in RNA libraries

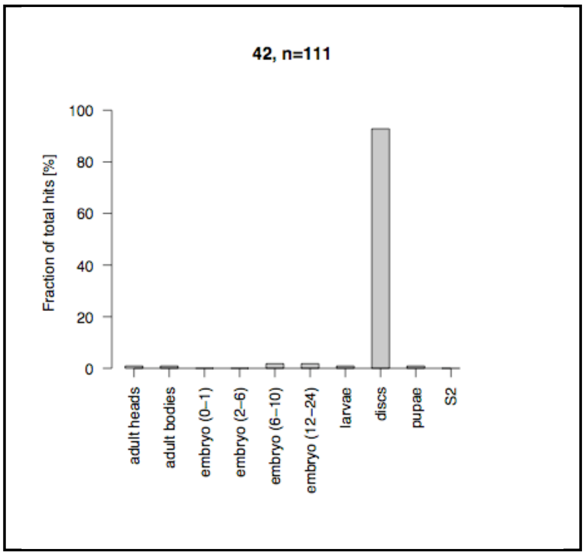

miRNA 43

chrX : 9801266 - 9801283 **Transcribed strand: +**

predicted as:

Validated: detected in small RNA sequencing data

Mature sequence: GUUUGAAUGUGUUUAUGU

conservation profile

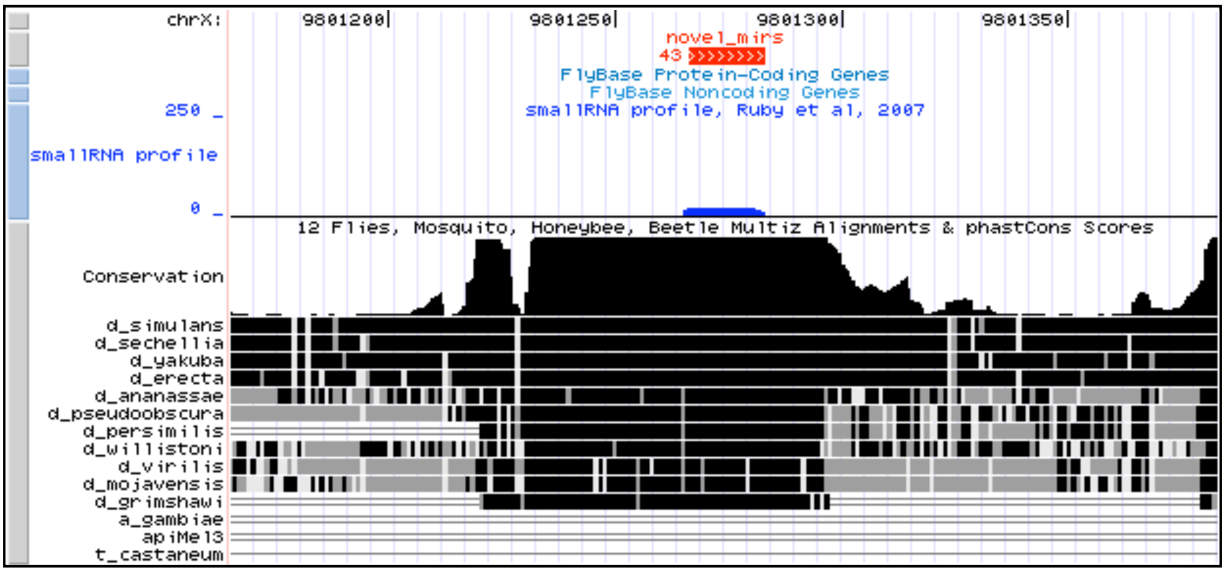

genomic context

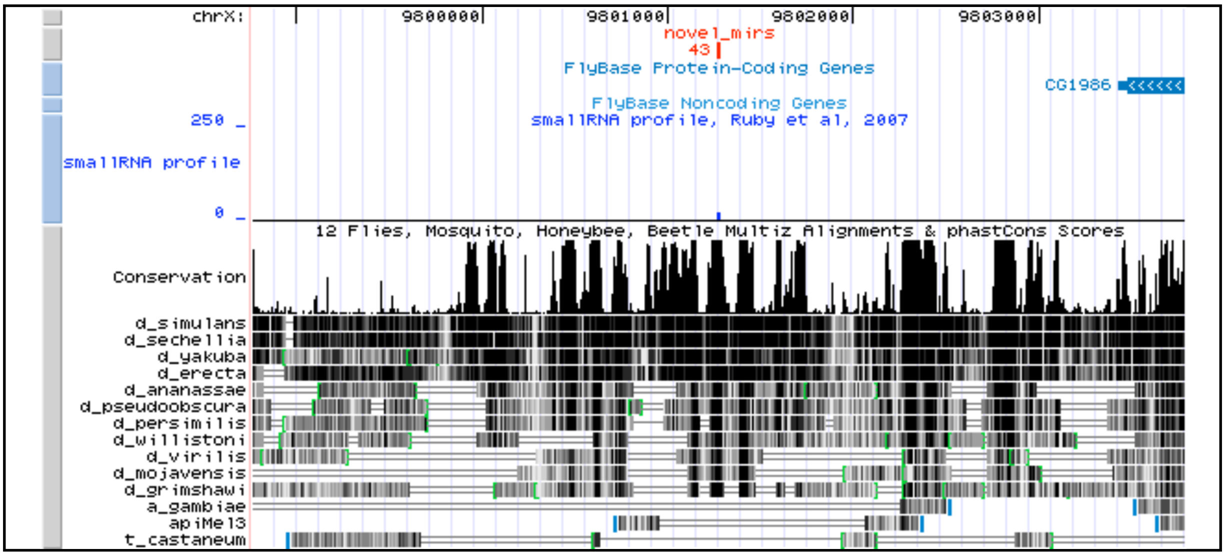

rnafold / alifold prediction

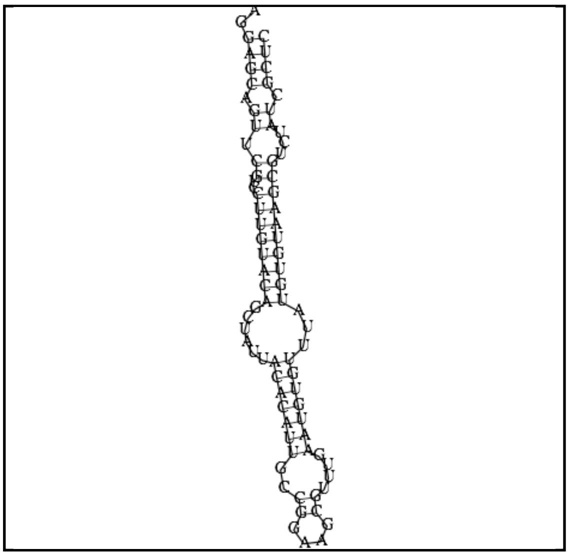

Hits in RNA libraries

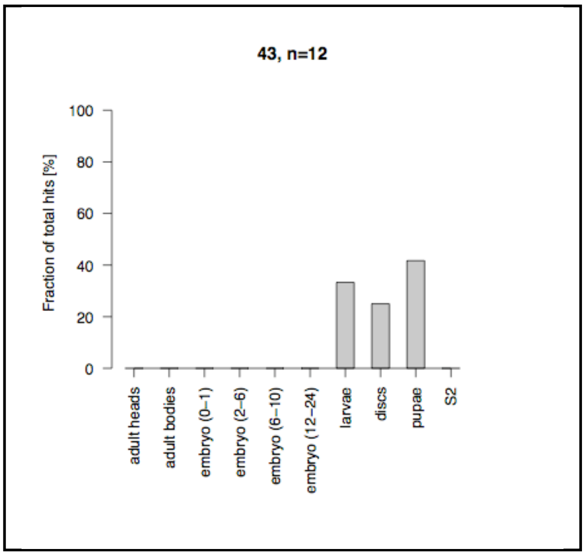

miRNA 44

chrX : 12530429 - 12530451 Transcribed strand: +

predicted as:

Validated: detected in small RNA sequencing data

Mature sequence: UCAUAAGACACACGCGGCUAU

conservation profile

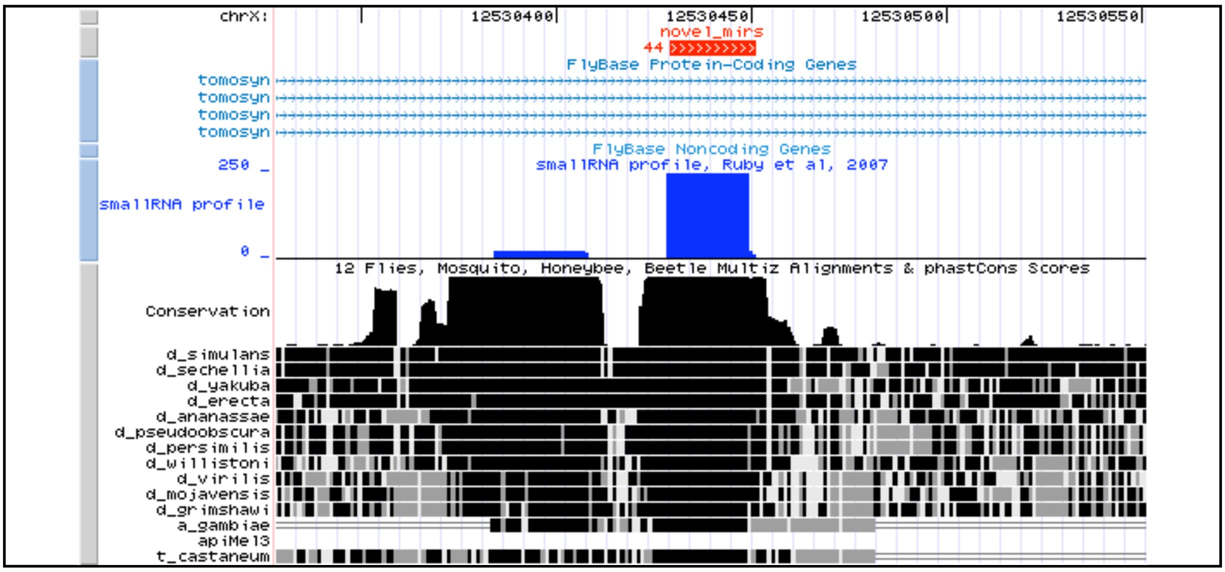

genomic context

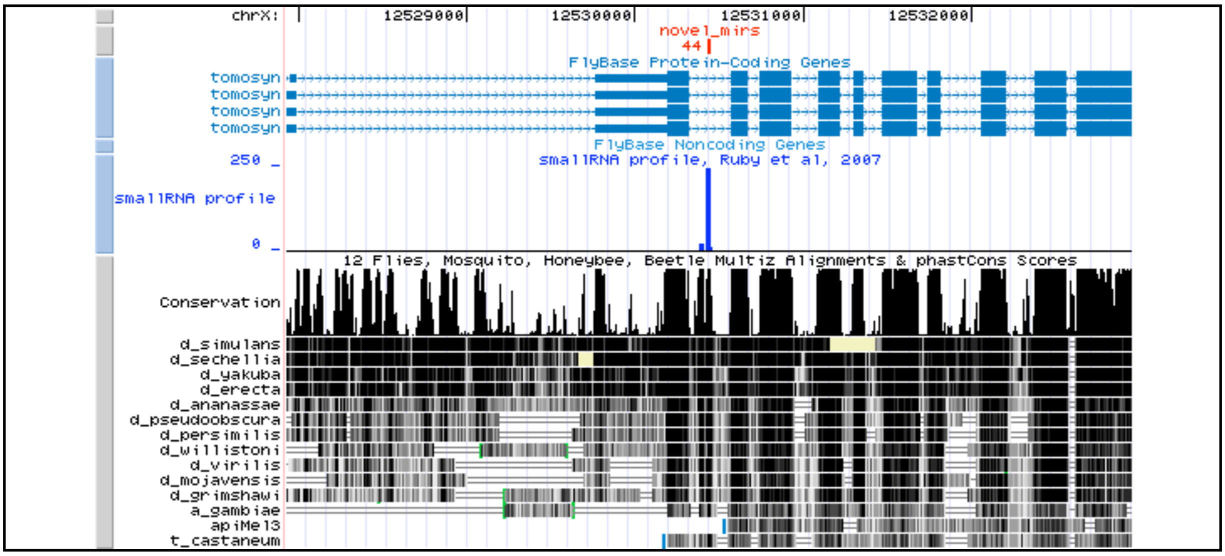

rnafold / alifold prediction

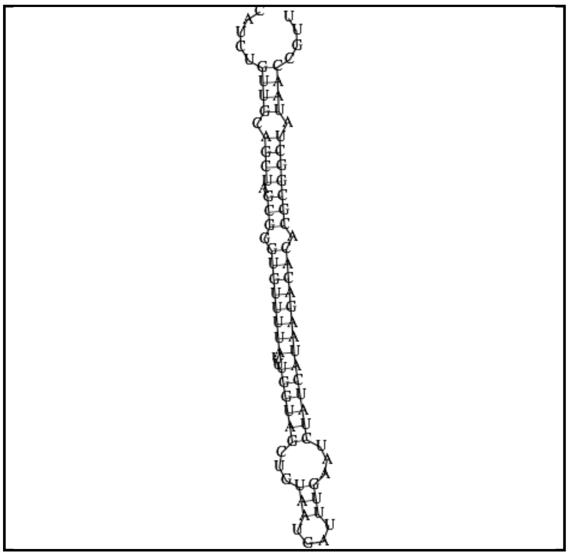

Hits in RNA libraries

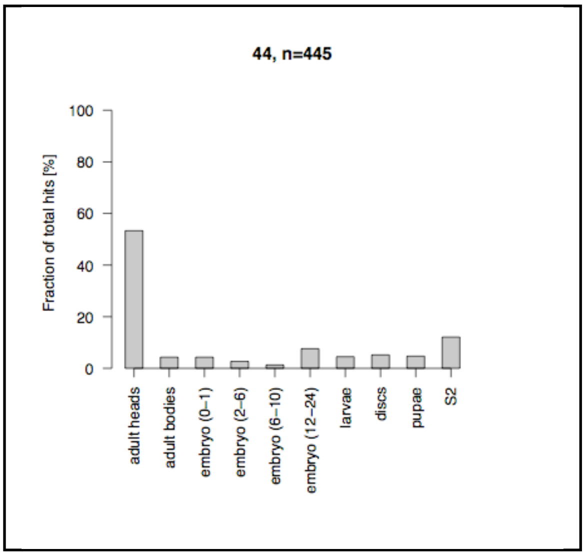

miRNA 45 chrX : 12956164 - 12956185 Transcribed strand: +

predicted as:

Validated: detected in small RNA sequencing data

Mature sequence: UUGGUGUUACUUCUUACAGUGA

conservation profile

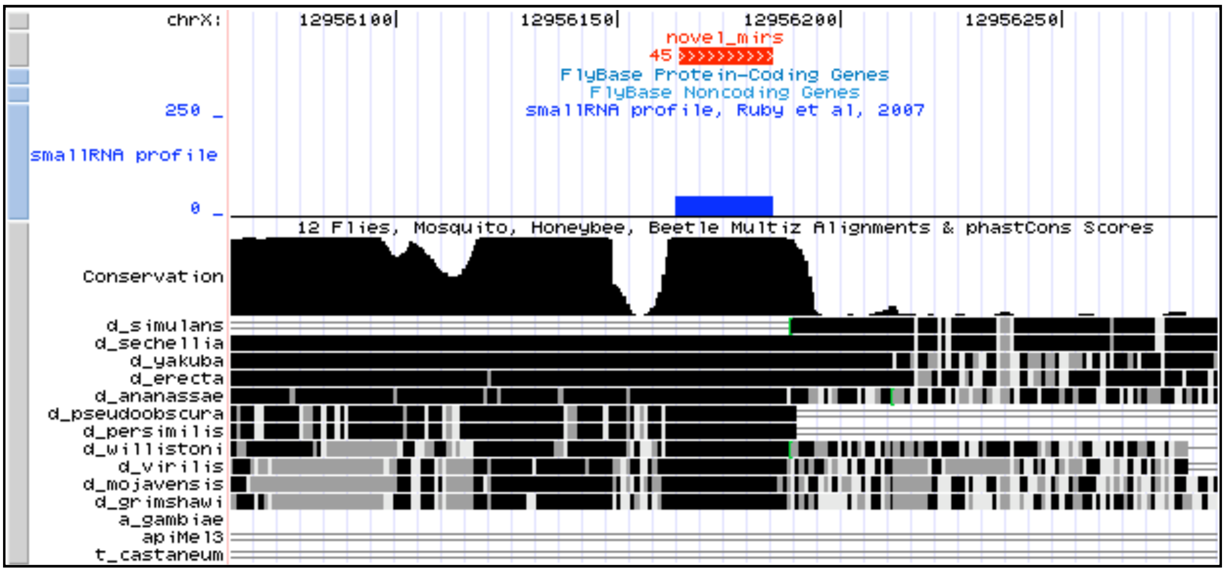

genomic context

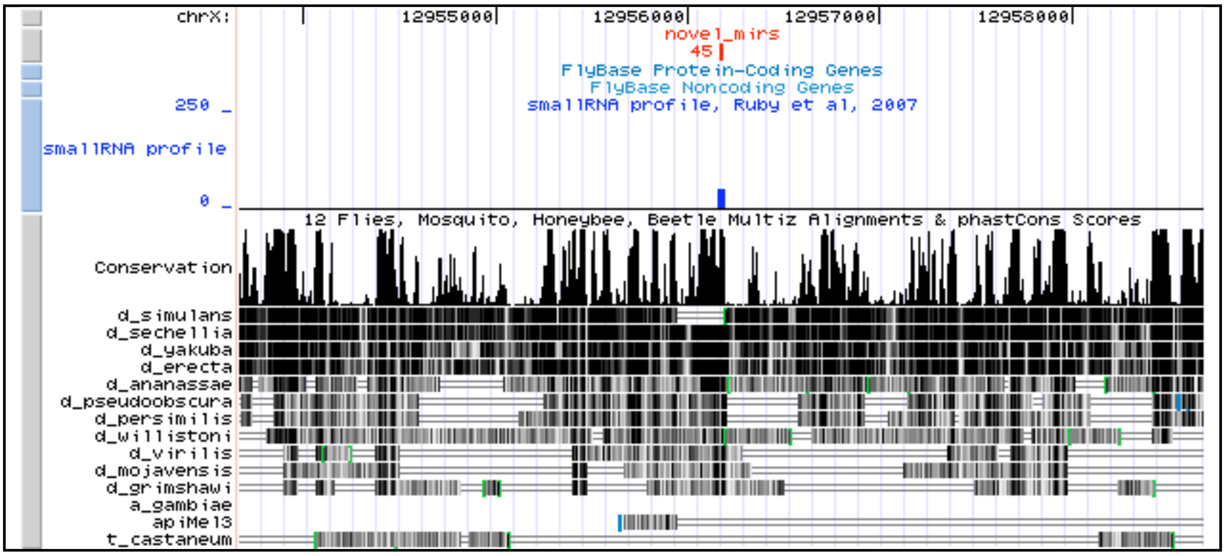

rnafold / alifold prediction

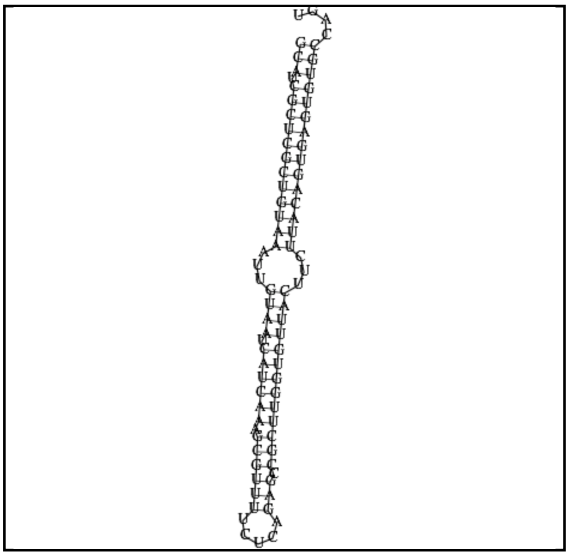

Hits in RNA libraries

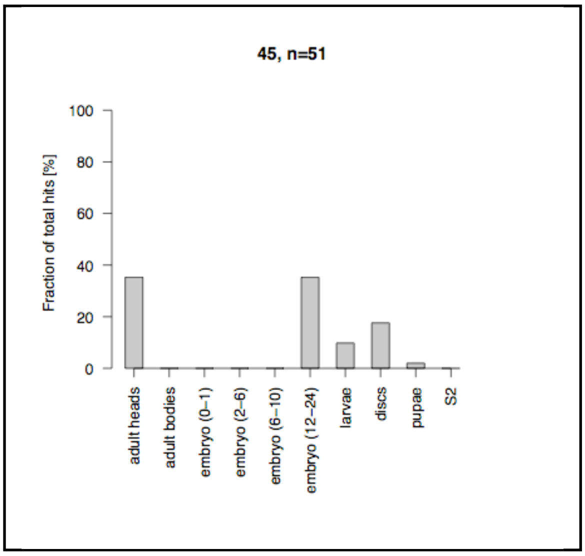

miRNA 46

chrX : 15860034 - 15860057 Transcribed strand: -

predicted as: locus15

Validated: detected in small RNA sequencing data, validated by Northern blot

Mature sequence: UUUAGAAUUCCUACGCUUUACC

conservation profile

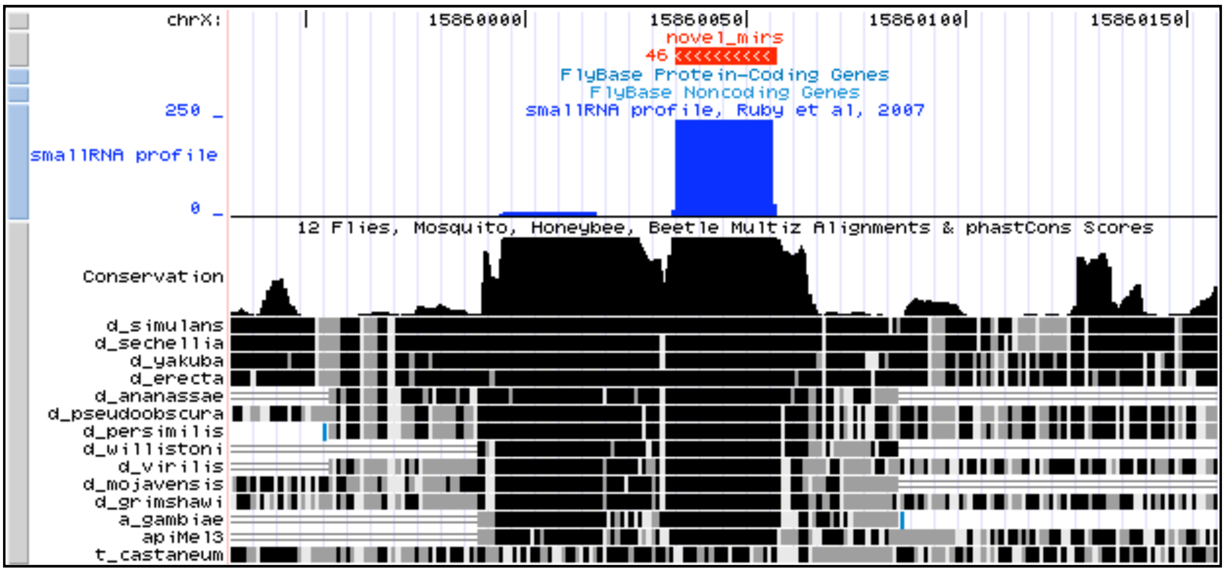

genomic context

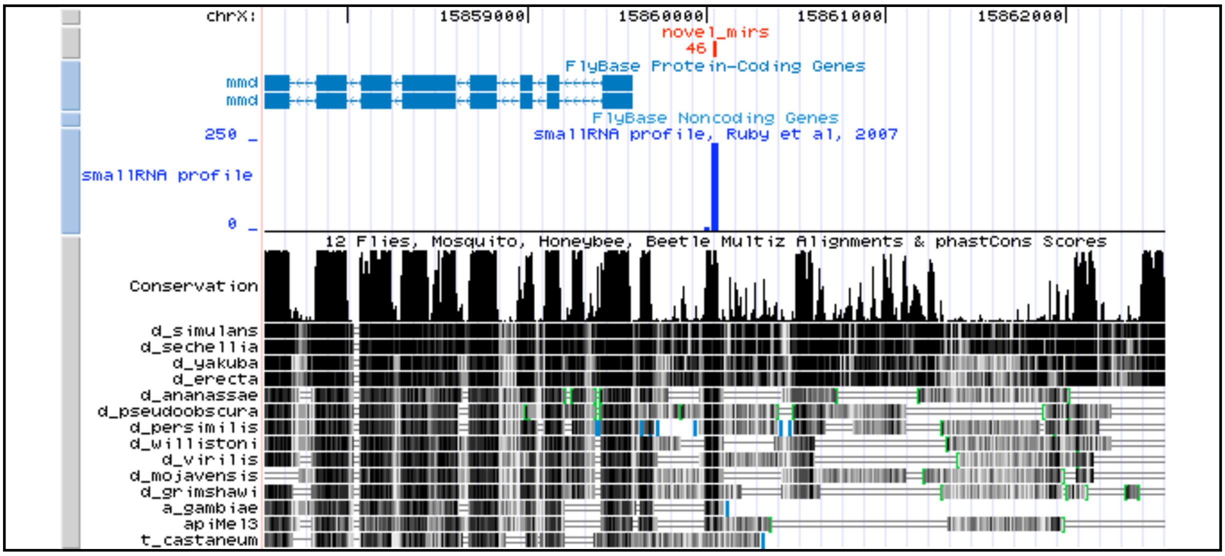

rnafold / alifold prediction

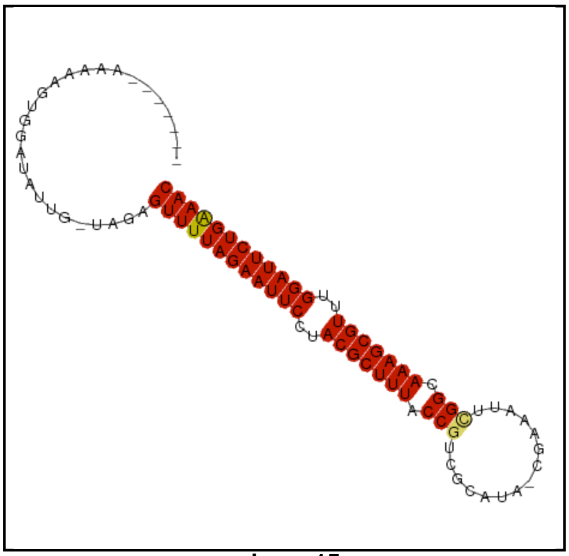

locus15-

Hits in RNA libraries

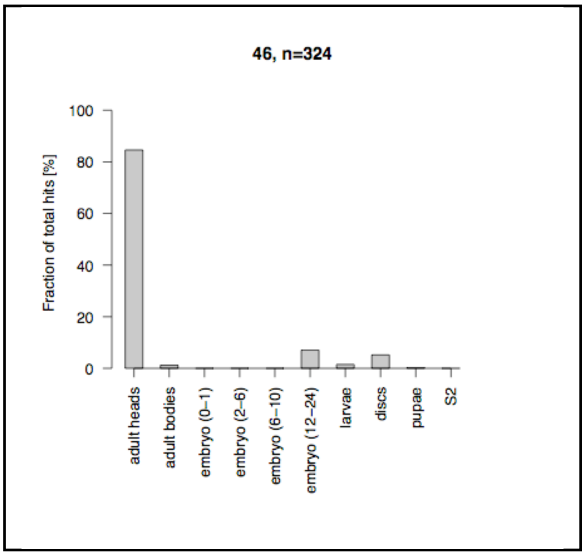

miRNA 47

chrX : 19445579 - 19445603   Transcribed strand: +

predicted as:

Validated: detected in small RNA sequencing data

Mature sequence: UGGUUGGUGGUUGAACUUCGAUUUU

conservation profile

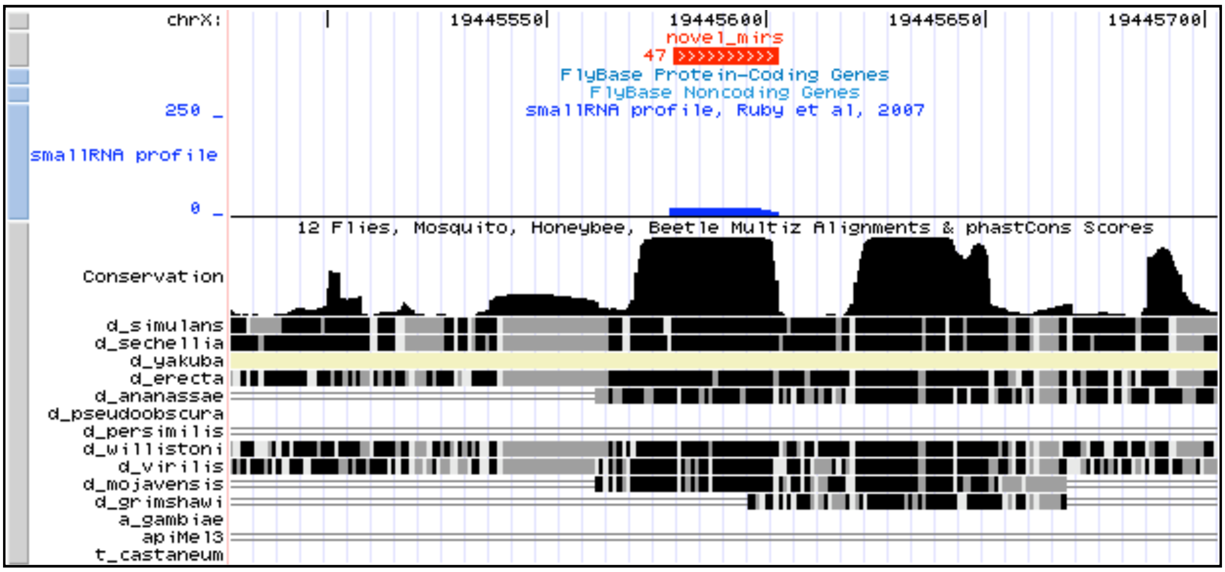

genomic context

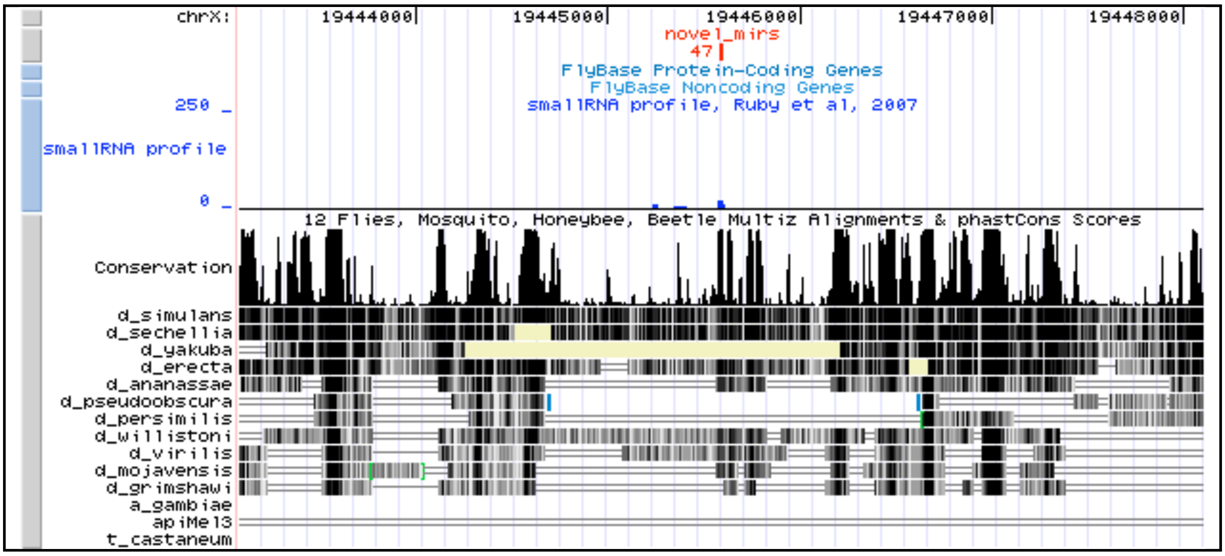

rnafold / alifold prediction

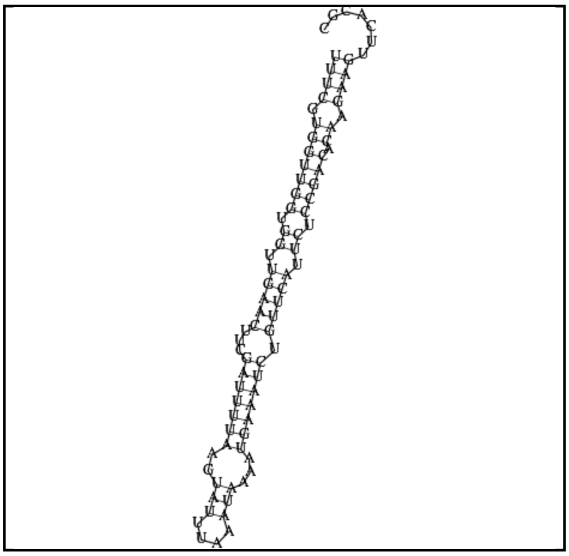

Hits in RNA libraries

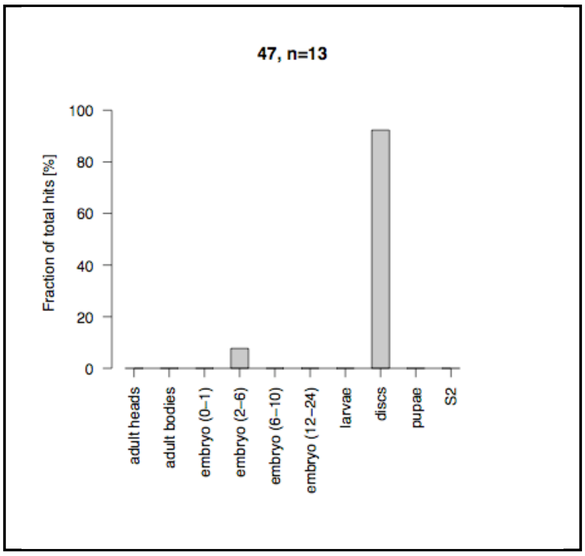

miRNA 48

chrX : 19452463 - 19452484 Transcribed strand: +

predicted as:

Validated: detected in small RNA sequencing data

Mature sequence: UGUUCAACAUAUUUAGCACUCA

conservation profile

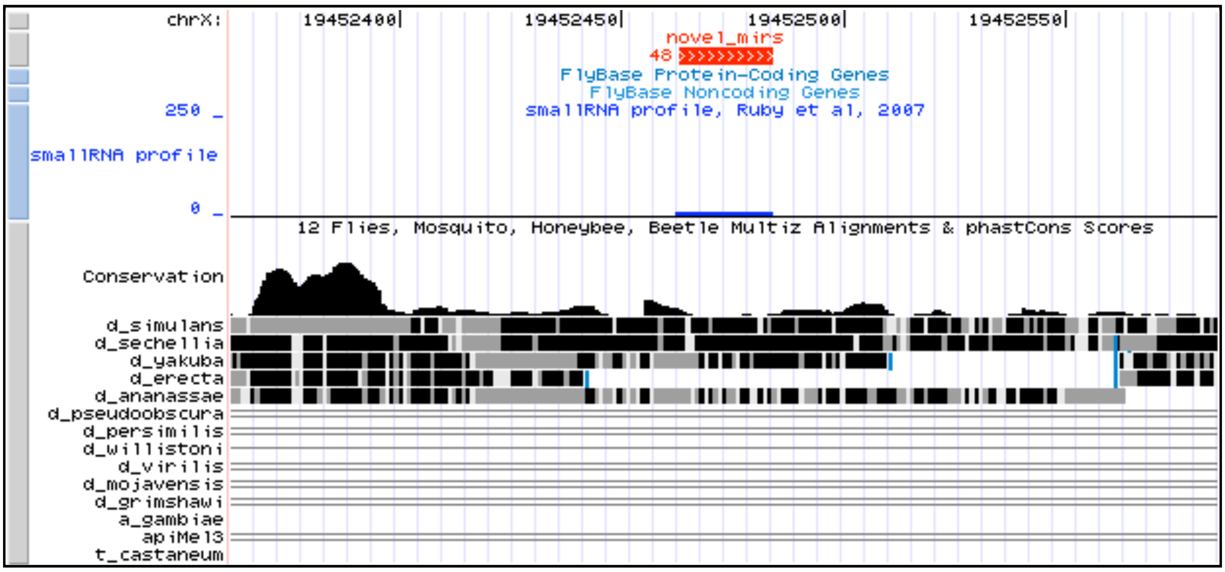

genomic context

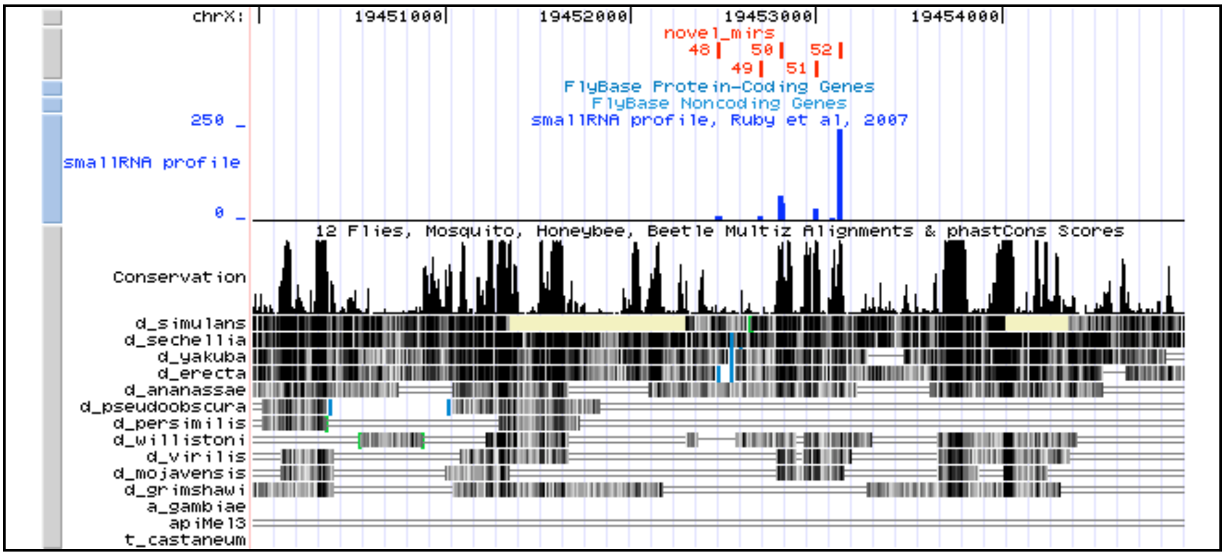

rnafold / alifold prediction

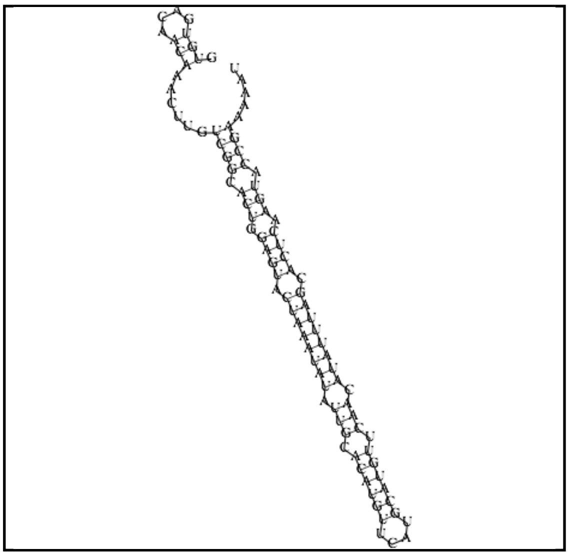

Hits in RNA libraries

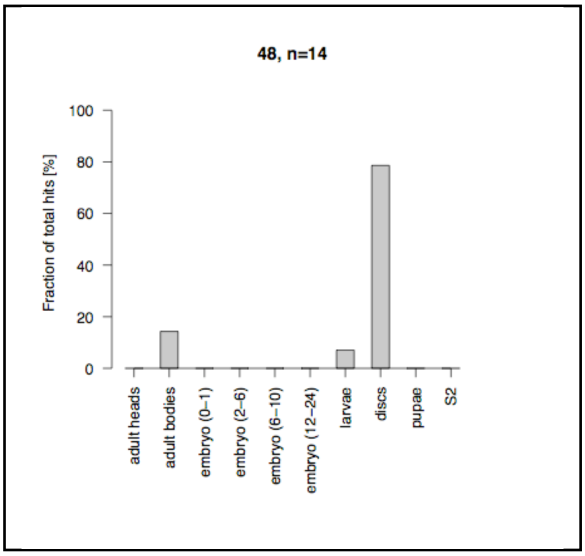

miRNA 49

chrX : 19452692 - 19452713 Transcribed strand: +

predicted as:

Validated: detected in small RNA sequencing data

Mature sequence: UGUUCAACAUAUUUAGCACUCA

conservation profile

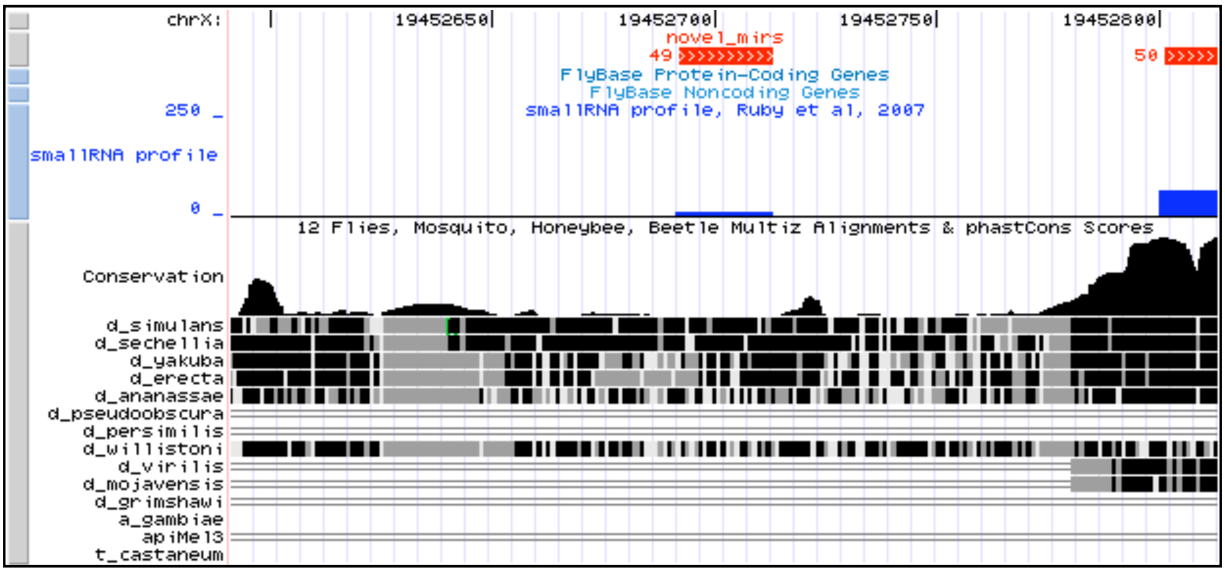

genomic context

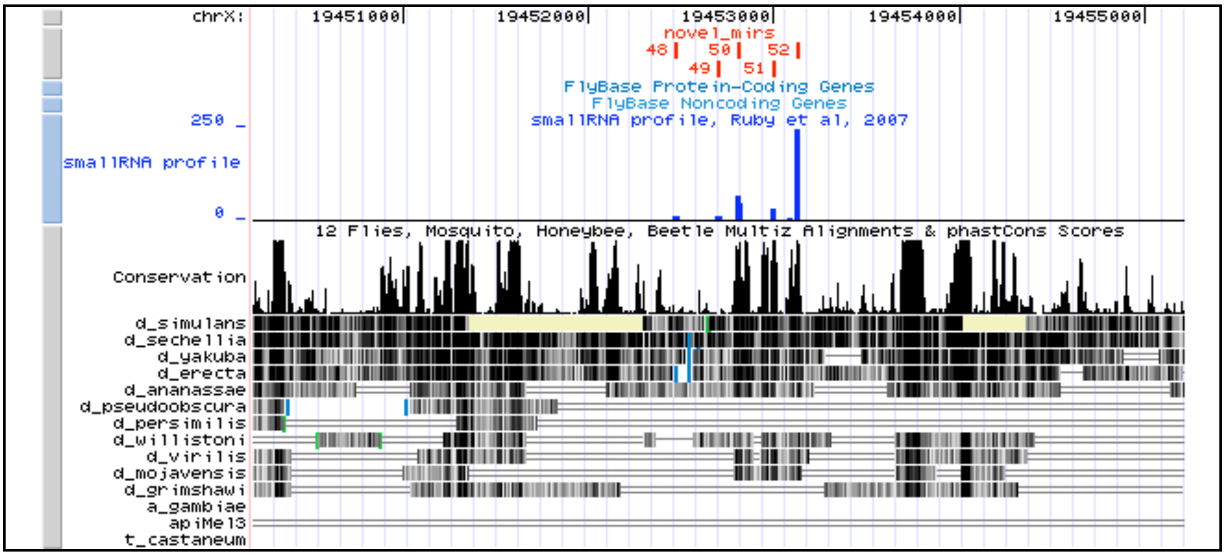

rnafold / alifold prediction

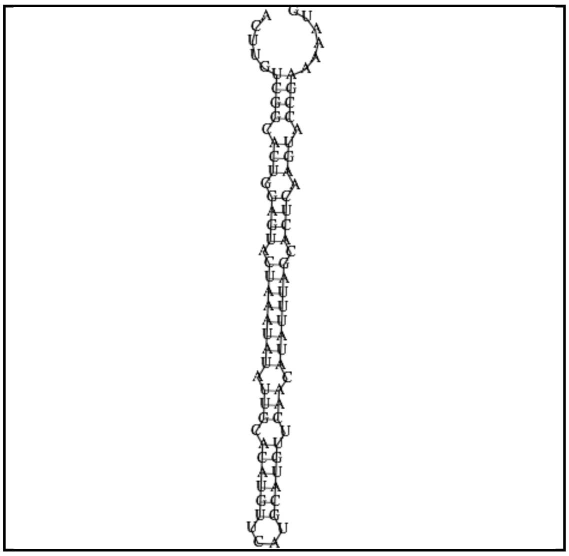

Hits in RNA libraries

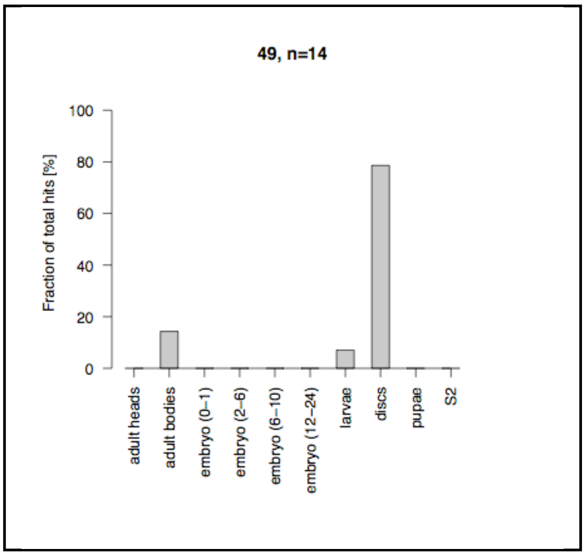

miRNA 50

chrX : 19452801 - 19452823 Transcribed strand: + cluster1

predicted as: manual8

Validated: detected in small RNA sequencing data

Mature sequence: UAAACACUUCCUACAUCCUGUAU

conservation profile

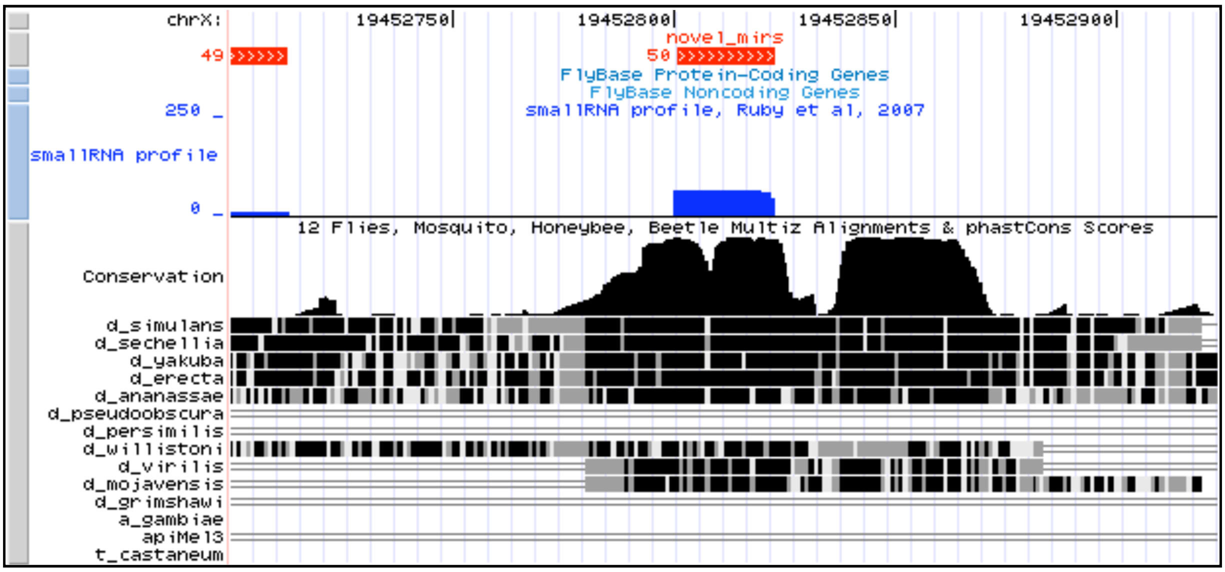

genomic context

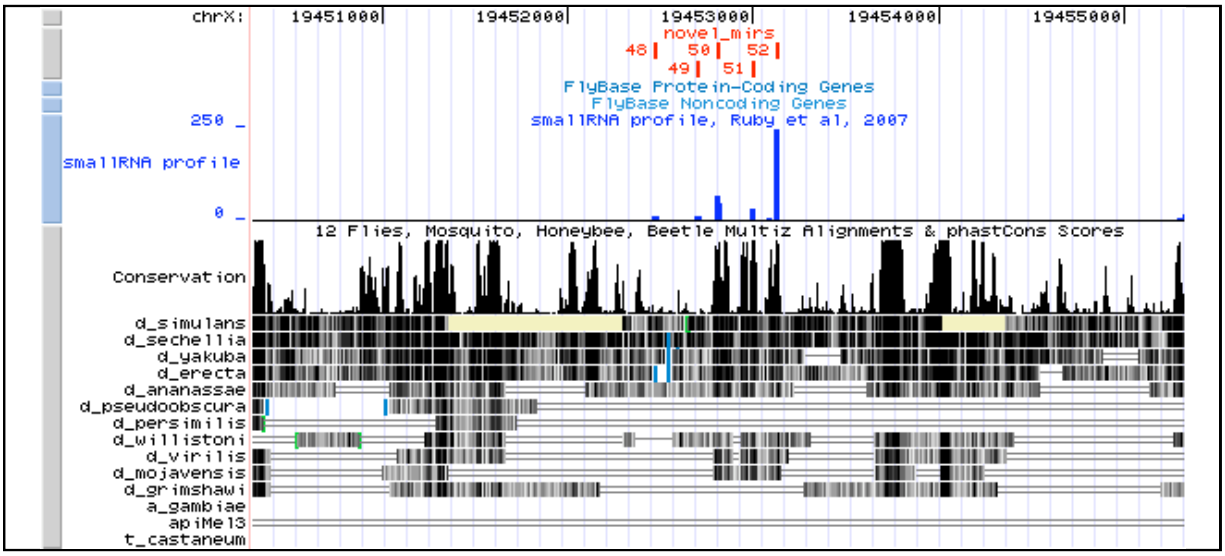

rnafold / alifold prediction

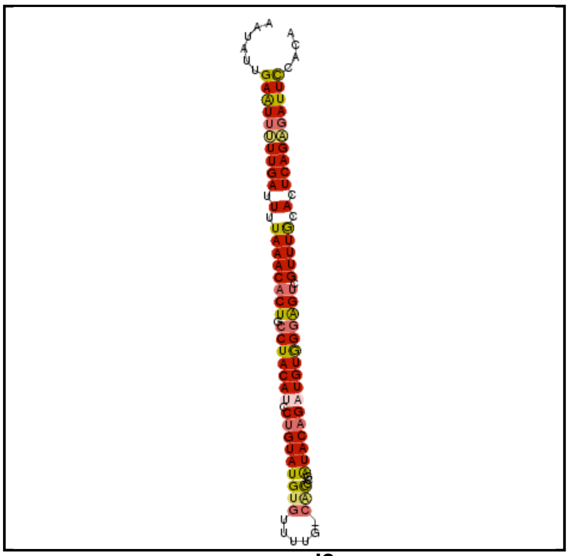

manual8+

Hits in RNA libraries

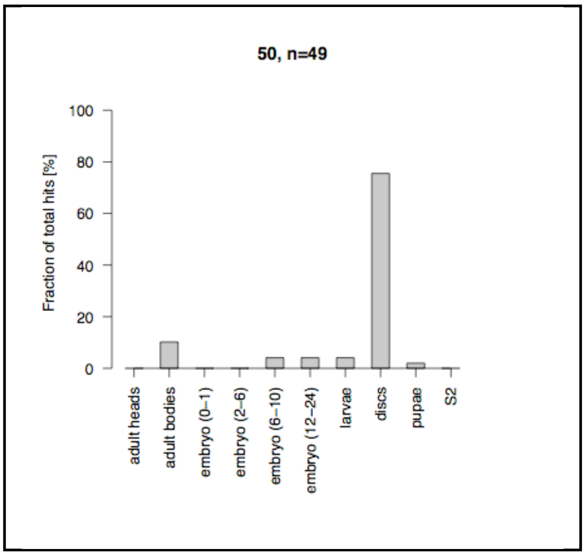

miRNA 51 chrX : 19452988 - 19453008 Transcribed strand: + **cluster1**

predicted as: locus1

Validated: detected in small RNA sequencing data, validated by Northern blot

Mature sequence: UUGGAUUAGUUAUCAUCAAUGC

conservation profile

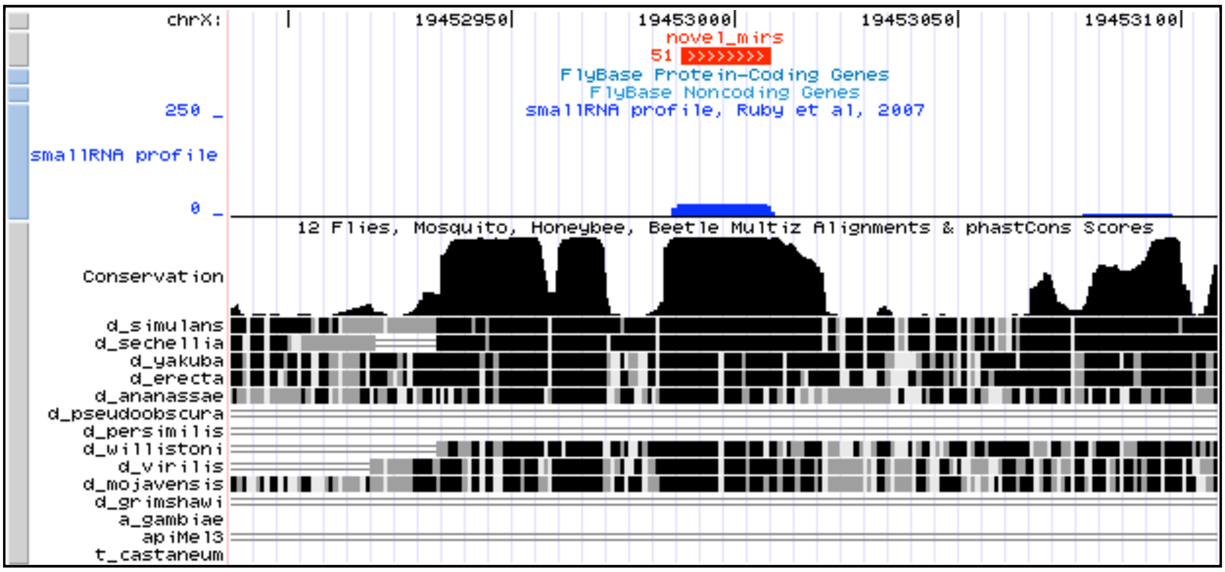

genomic context

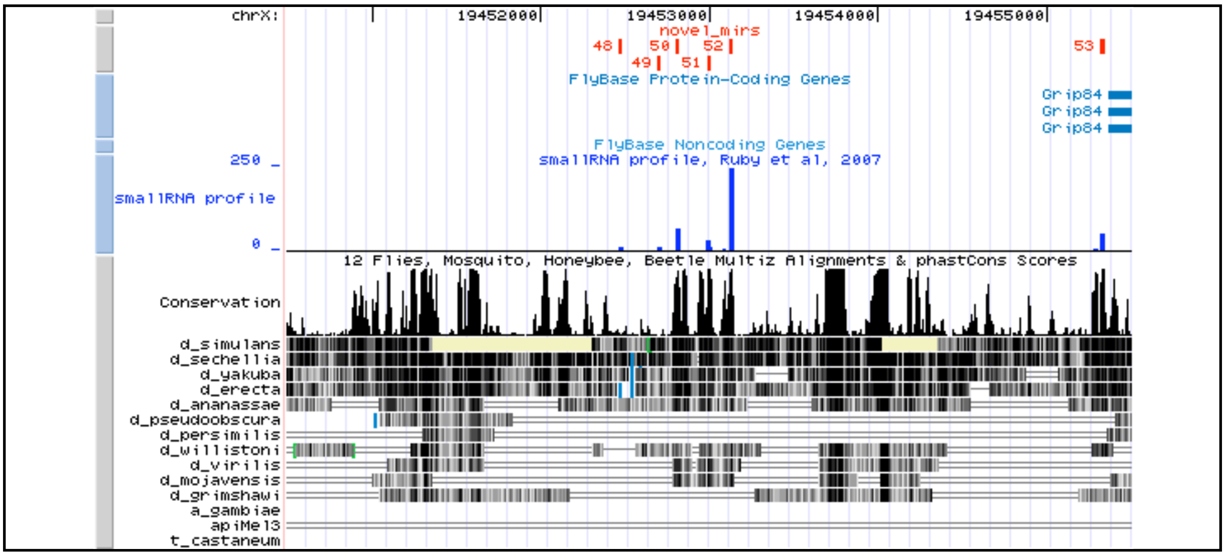

rnafold / alifold prediction

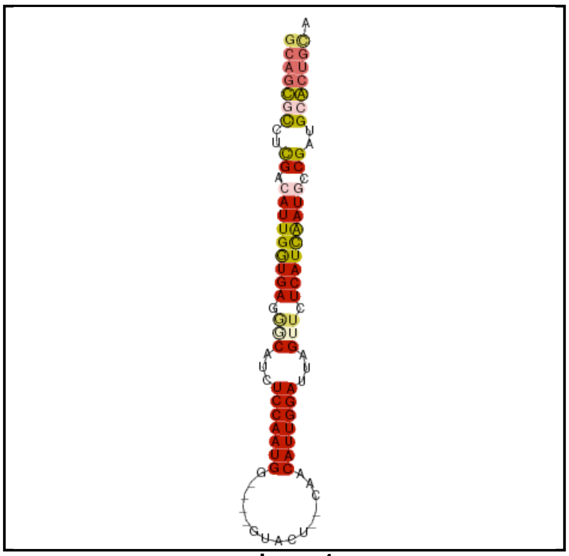

locus1+

Hits in RNA libraries

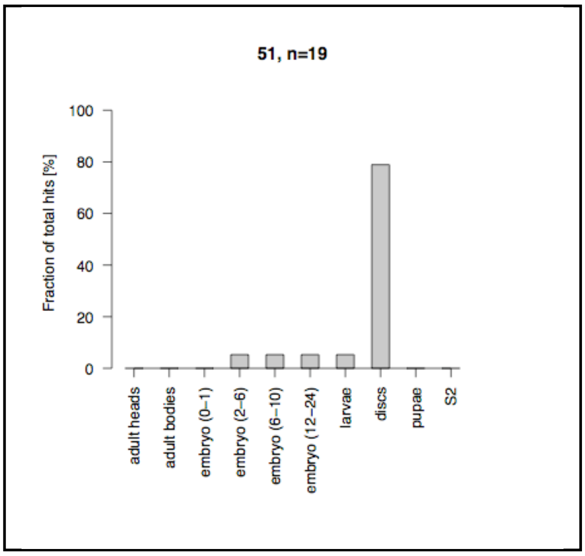

miRNA 52

chrX : 19453121 - 19453140   Transcribed strand: +   cluster1

predicted as: manual9

Validated: detected in small RNA sequencing data, validated by Northern blot

Mature sequence: UGAGAUAUUCACGUUGUCUAA

conservation profile

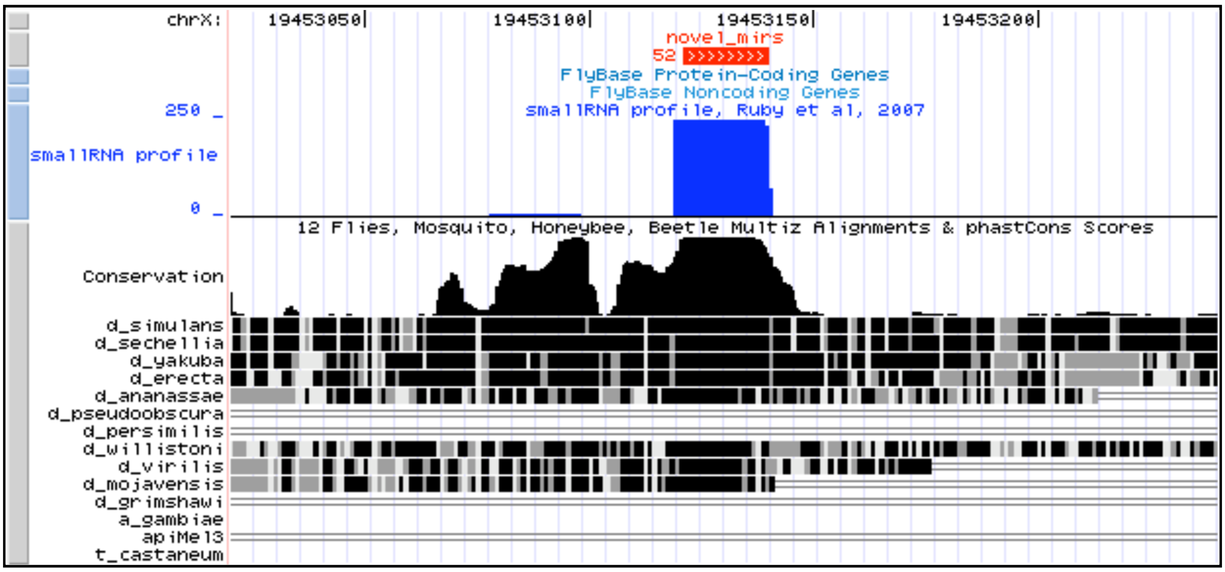

genomic context

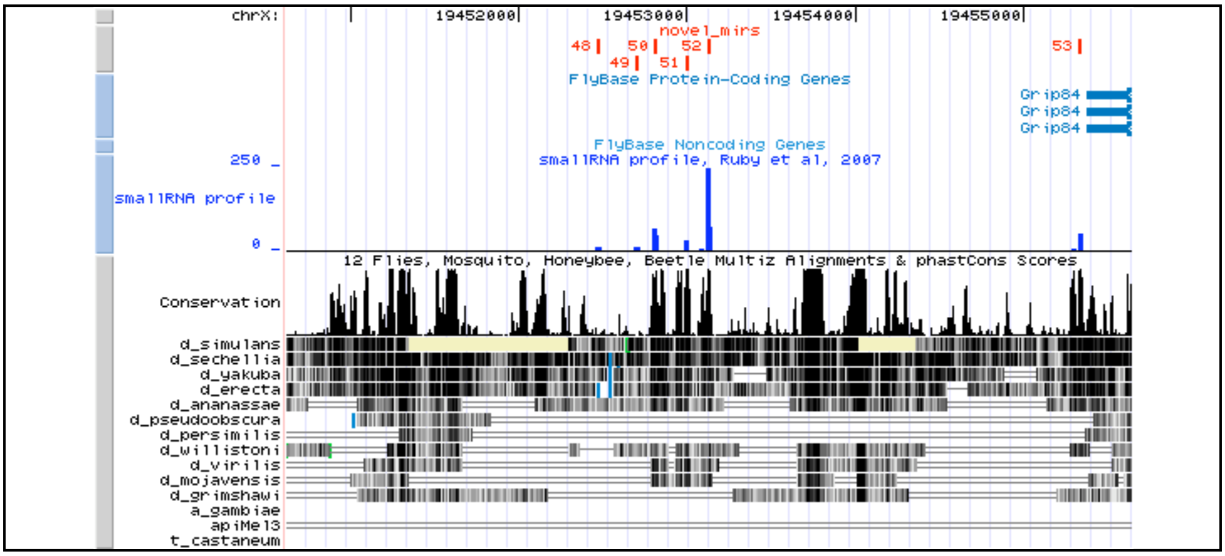

rnafold / alifold prediction

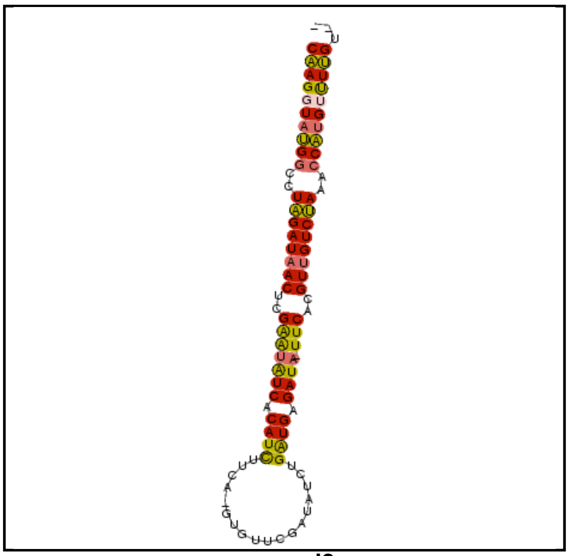

manual9+

Hits in RNA libraries

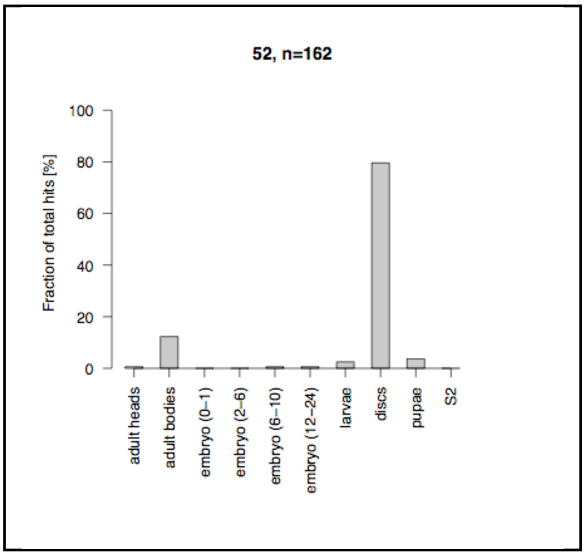

miRNA 53

chrX : 19455323 - 19455345 Transcribed strand: +

predicted as:

Validated: detected in small RNA sequencing data

Mature sequence: UGUCCAGUGCCGUAAAUUGCAG

conservation profile

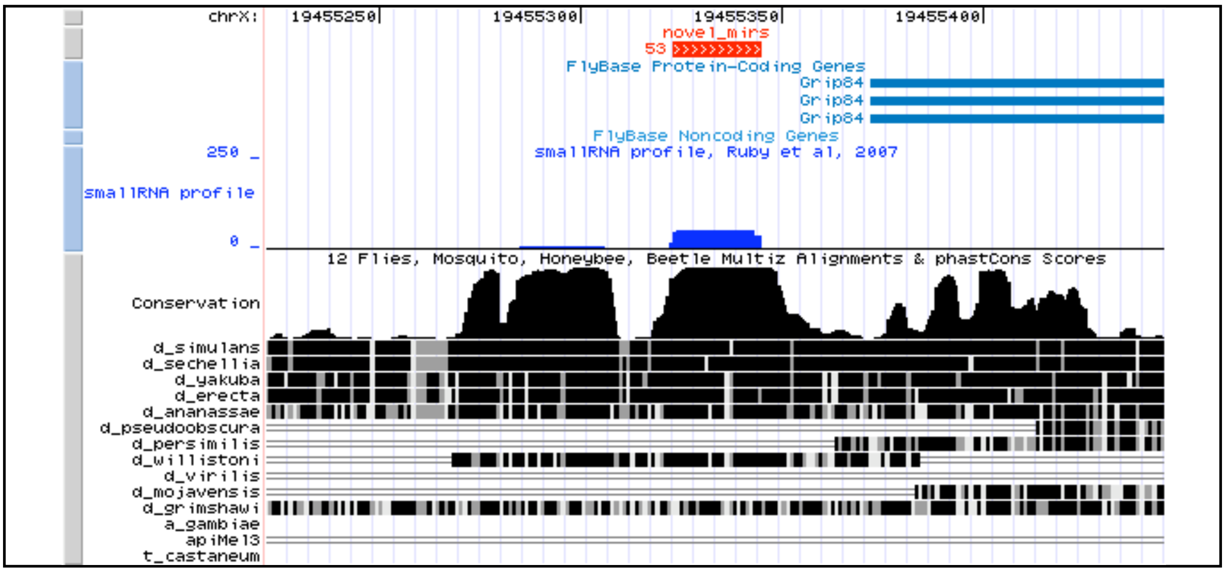

genomic context

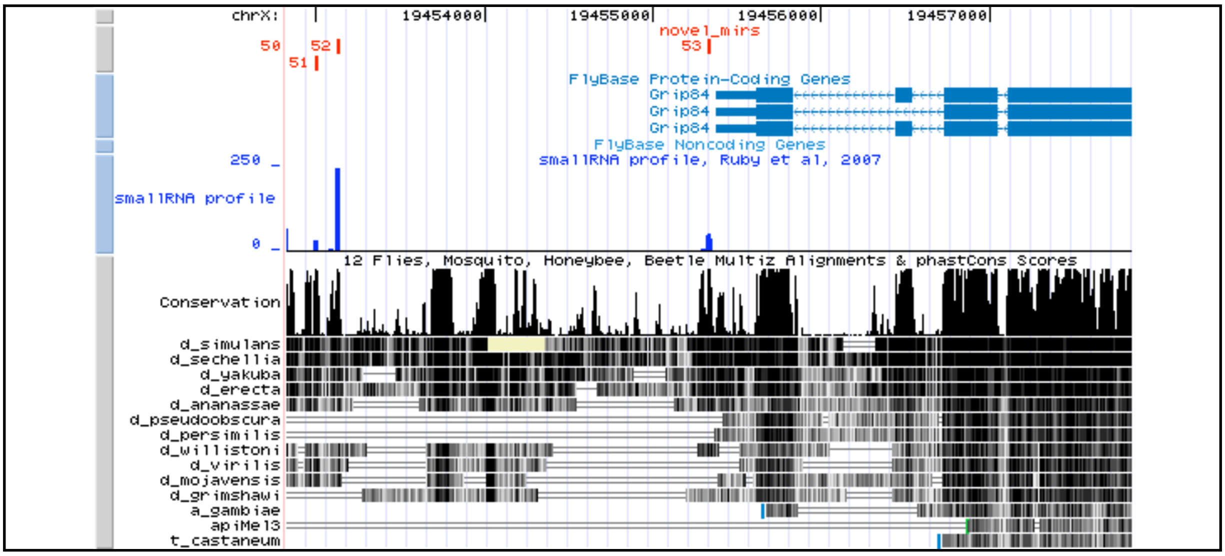

rnafold / alifold prediction

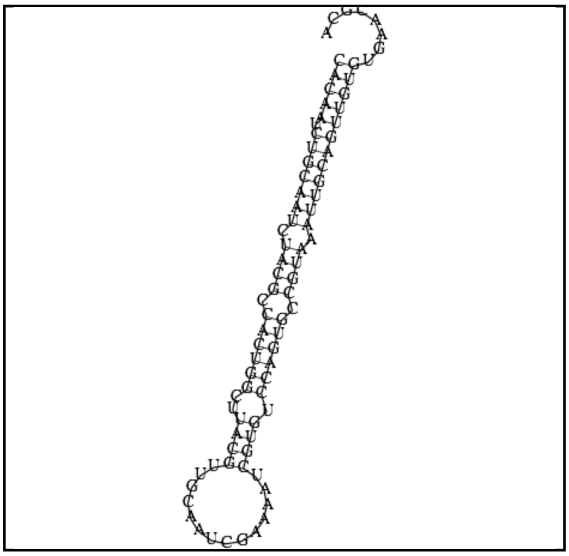

Hits in RNA libraries

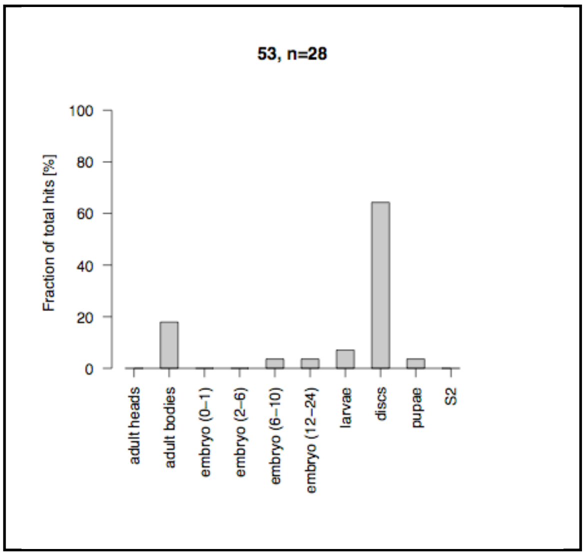

Supplement: Data S1 — Pdf document containing information about all validated novel miRNAs, including RNAz alignment and structure models. (2.17 MB PDF) [file pone.0001265.s001.pdf]
